# Supplementary material for: Naked d-orbital in a centrochiral Ni(II) complex as a catalyst for asymmetric [3+2] cycloaddition
Source: Nat Commun. 2017 Apr 6;8:14875. doi: 10.1038/ncomms14875 (PMC5384211; doi:10.1038/ncomms14875)
Supplement: Supplementary Information — Supplementary figures, supplementary tables, supplementary note, supplementary methods and supplementary references. [file ncomms14875-s1.pdf]

Supplementary Figures

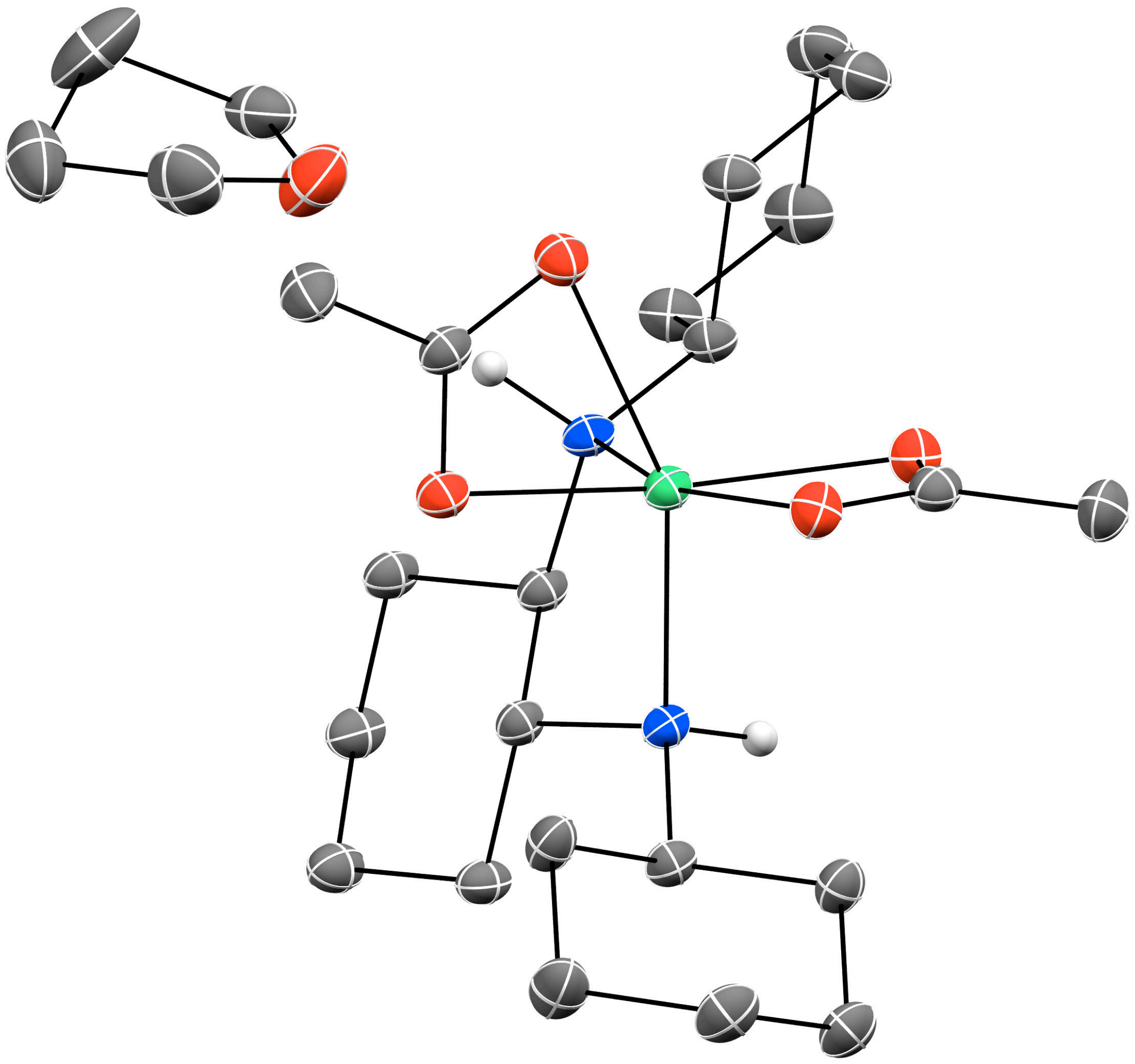

Supplementary Figure 1 | ORTEP drawing of Mononuclear Ni(II) complex I (CCDC 1482739). Thermal ellipsoids are shown at the with 50% probability level. Hydrogen atoms on carbons and the minor disorder component of THF are omitted for the sake of clarity.

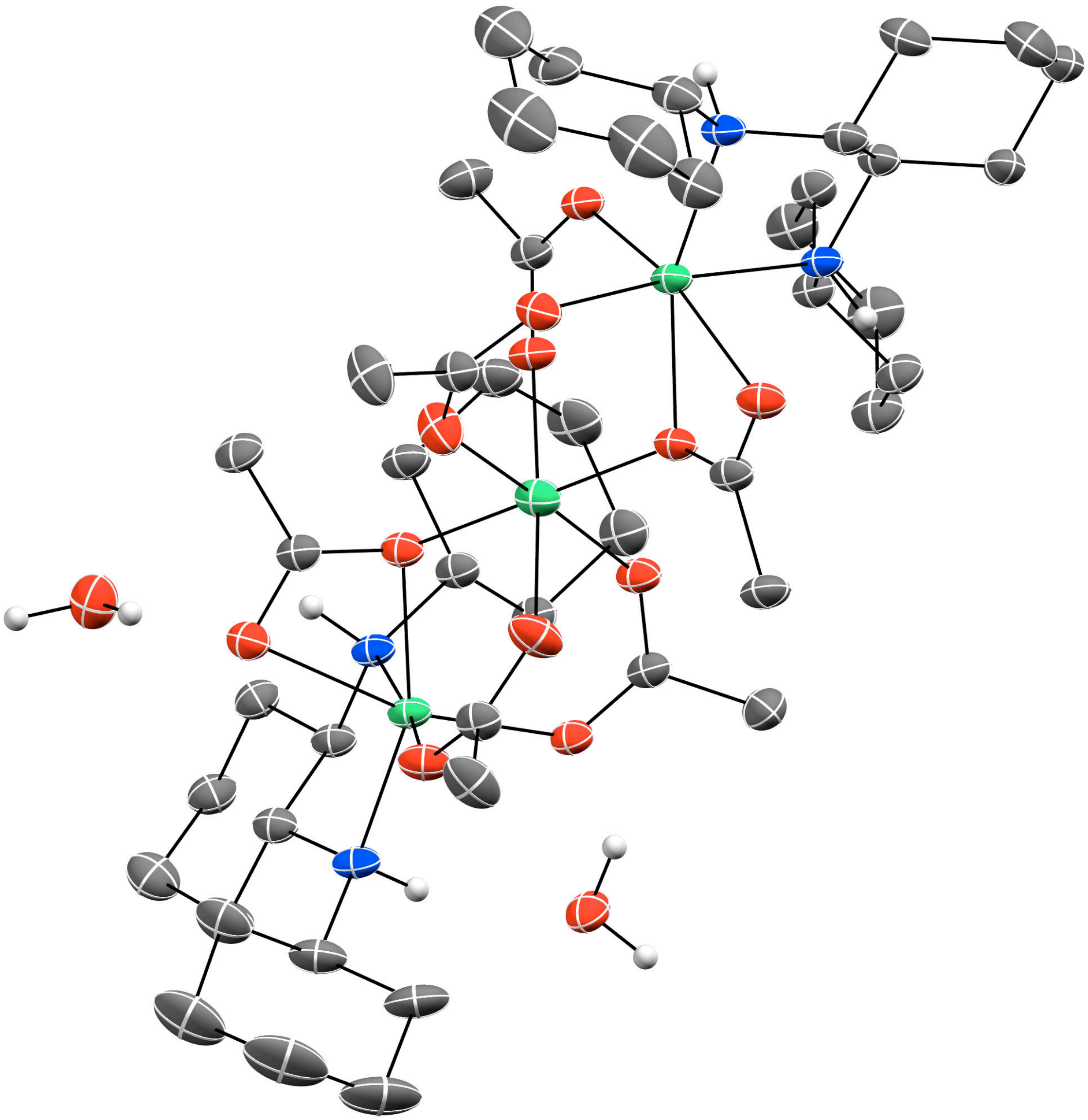

Supplementary Figure 2 | ORTEP drawing of Trinuclear Ni(II) complex II (CCDC 1482740). Thermal ellipsoids are shown at the with 50% probability level. Hydrogen atoms on carbons and the minor disorder component of THF are omitted for the sake of clarity.

One of the most striking features of the chiral nickel(II)–diamine(s)–acetates **I** and **II** (Fig. 2, main text) is the noncentrosymmetric octahedral Ni(II)–diamine(s)–acetates complexes that involve the chiral-at-metal center, in which C_2-symmetric diamine and the acetate anion are desymmetrized.

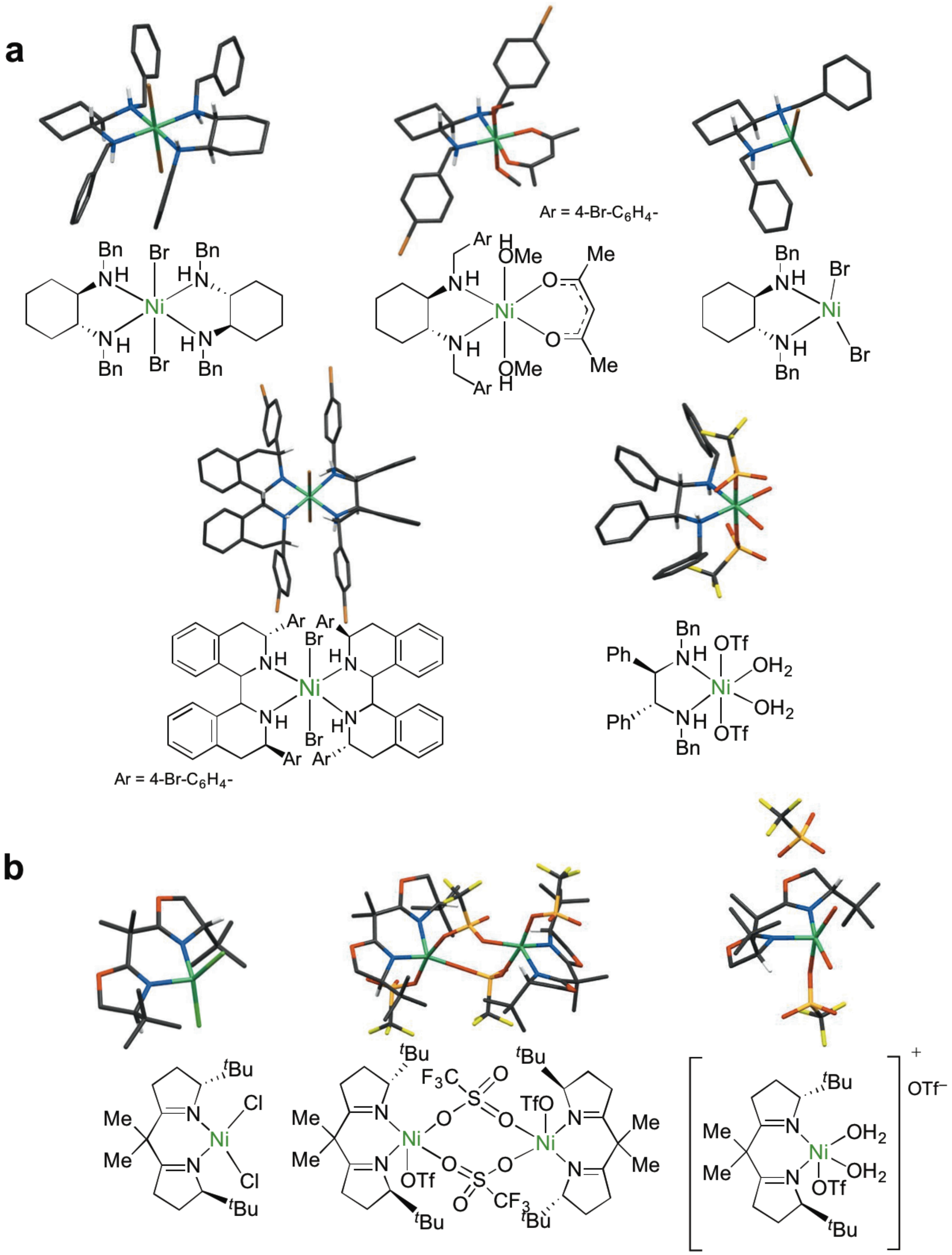

Supplementary Figure 3 | Selected examples of chiral Ni catalysts with a chiral bidentate ligand(s). (**a**) Ni(II)–di-*sec*-amine[1-4]. (**b**) Ni(II)–bis(oxazoline)[5]. In the stick illustrations, H atoms except for those on chiral carbons are omitted for the sake of clarity. Bn, benzyl; TfO, trifluoromethanesulfonate.

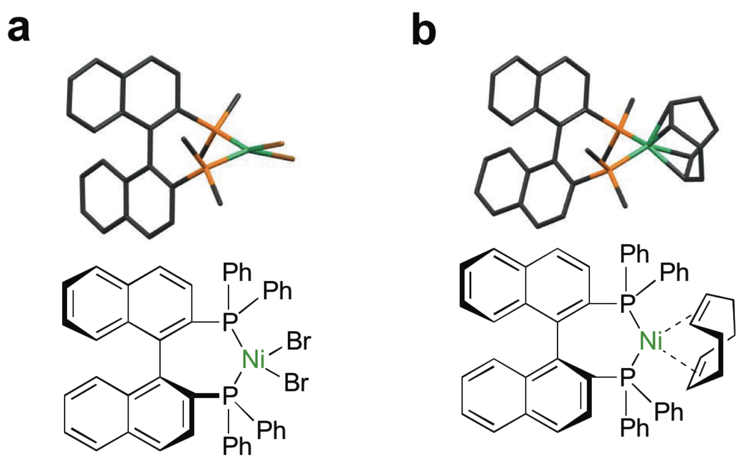

Supplementary Figure 4 | Selected examples of Ni catalysts with a chiral diphosphine ligand. (**a**) Ni(II)–BINAP[6]. (**b**) Ni(0)–BINAP[6]. In the stick illustrations, H atoms except for those on chiral carbons are omitted, and phenyl groups on phosphines were replaced with methyl groups for the sake of clarity.

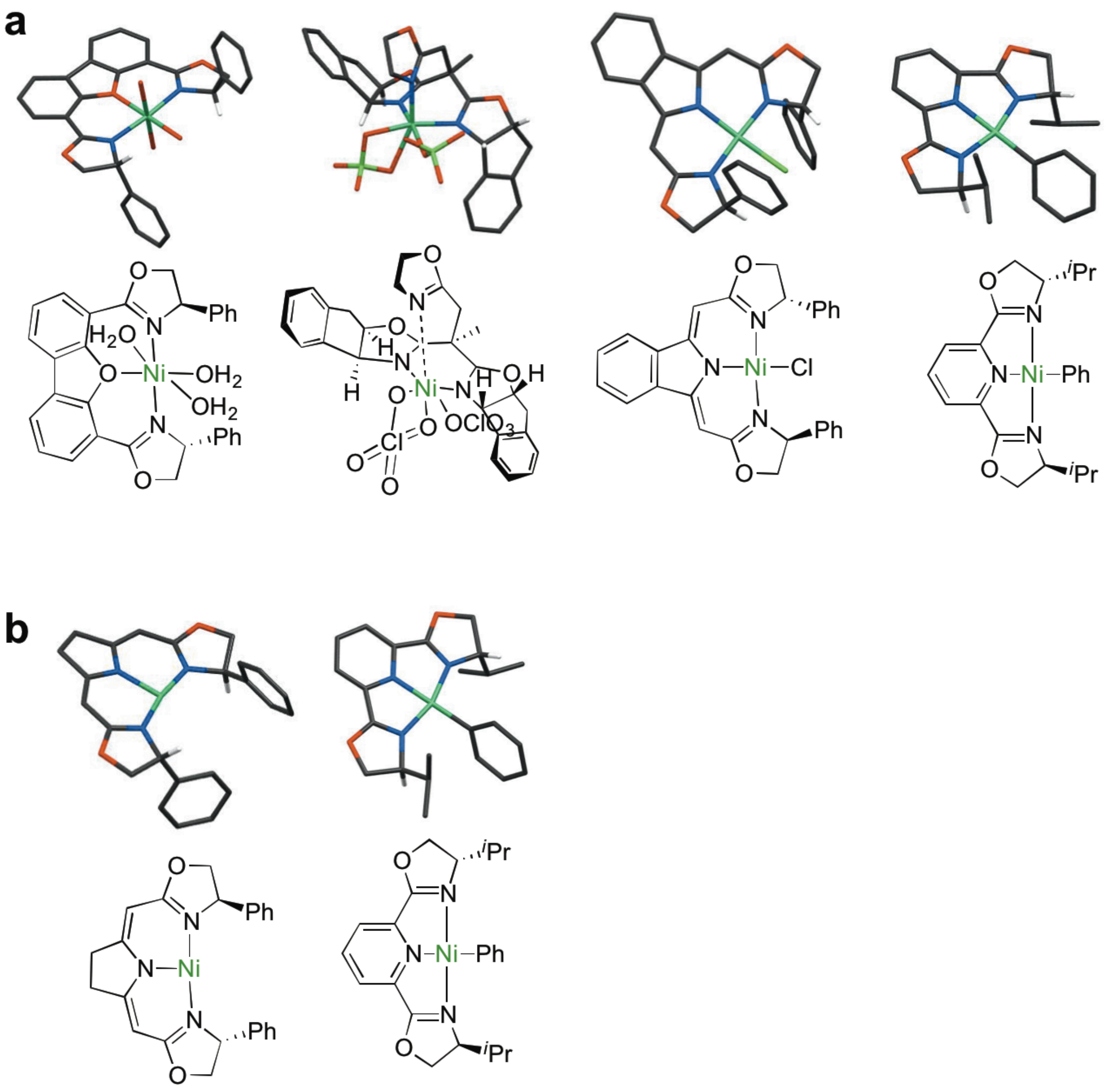

Supplementary Figure 5 | Selected examples of chiral Ni catalysts with a chiral tridentate ligand. (**a**) Ni(II)–tridentate ligand[7-10]. (**b**) Ni(I)-tridentate ligand[10,11]. Hydrogen atoms except for those on chiral carbons in the stick illustration are omitted for the sake of clarity.

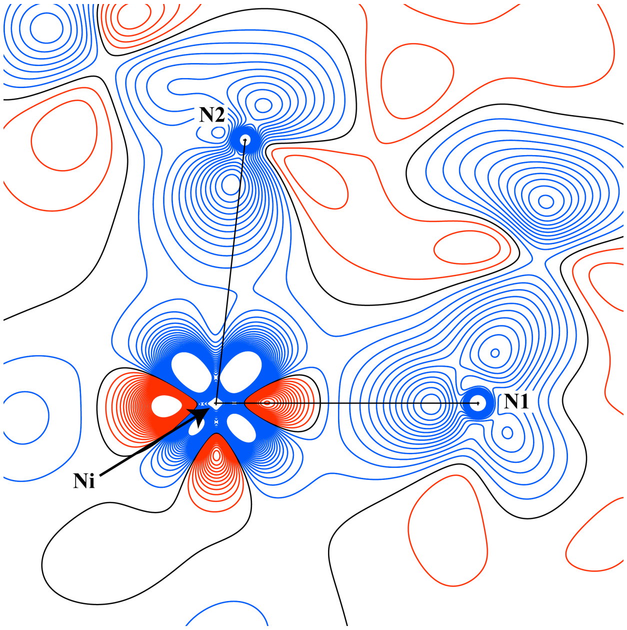

Supplementary Figure 6 | Static model map on the N(1)–Ni–N(2) plane; contours drawn at 0.05 e Å^{-3} interval in blue (positive), red (negative) and black (zero) lines.

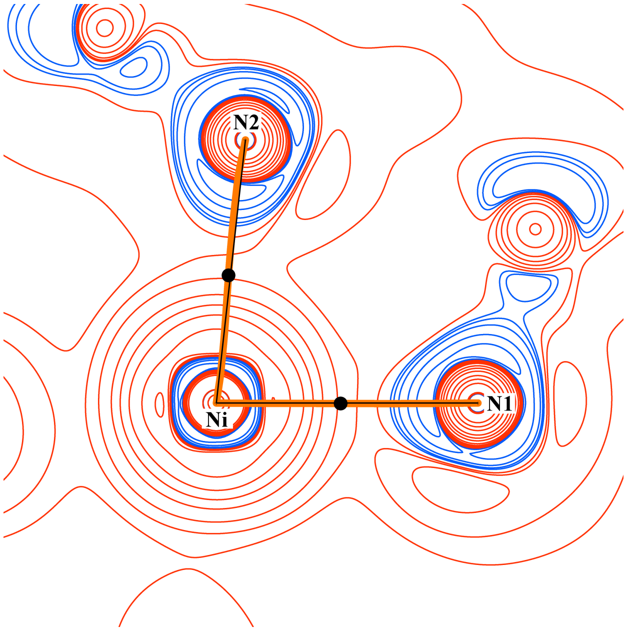

Supplementary Figure 7 | Laplacian distribution of total EDD on the N(1)–Ni–N(2) plane. The blue and red lines denote negative and positive Laplacian contours, respectively. The contours are drawn at ±2 × 10^n, ±4 × 10^n, ±8 × 10^n (where n = 0, 1, 2) e Å^{-5}. Bond path (BP) and bond critical points (BCPs) are depicted as orange lines and black dots, respectively.

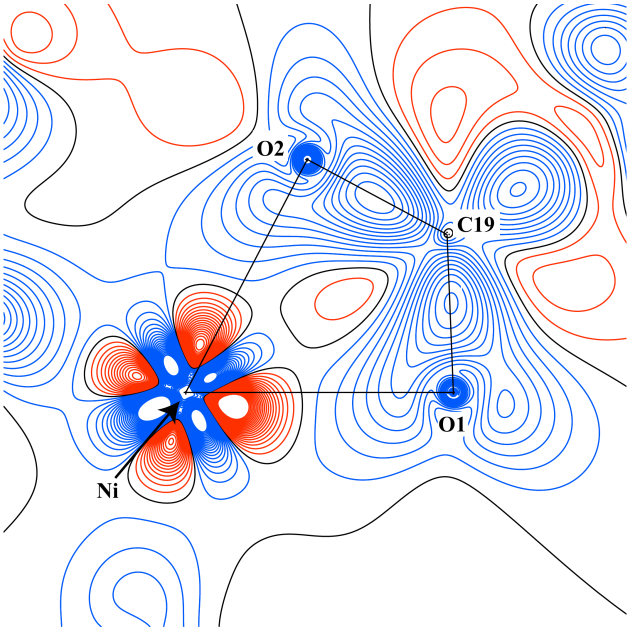

**Supplementary Figure 8 | **Static model map on the O(1)–Ni–O(2) plane; contours drawn at 0.05 e Å^{-3} interval in blue (positive), red (negative) and black (zero) lines.

To discuss the structure of Ni(II)–diamine–acetates complex **I** in the solution state, by comparison with the experimentally obtained IR and ECD spectra (Fig. 4, main text), we began the computational studies starting from the atomic coordinates obtained from the X-ray analysis of Ni(II)–diamine–acetates complex **I** (Supplementary Fig. 1). Considering the potential flexibility of the distorted Ni(II) center, the ModRedundant option was initially used, aiming at computationally reproducing the distorted pseudo-octahedral structure of Ni(II)–diamine–acetates complex **I**. The geometry optimizations with the ModRedundant option for fixing Ni, C, O and N gave **DFT-I** (Supplementary Fig. 9a) and **DFT-II** (Supplementary Fig. 9b) with a low single imaginary frequency (**DFT-I**: –57 cm^{-1} and **DFT-II**: –35 cm^{-1}). On the other hand, in geometry optimization without using the ModRedundant option, the bond length of Ni–O(4) in **DFT-III** (2.171 Å) was found to be shorter than the observed value [2.302(2) Å] in the mononuclear Ni–diamine–acetates complex **I** in the solid state (Supplementary Fig. 9c). **DFT-III** is found to be a local minimum on the potential energy surface (i.e., it has no imaginary frequencies). The unusually long distance between Ni(II) and O(4) in Ni(II)–diamine–acetates complex **I** determined by X-ray crystallography suggests a weak coordinating ability. Therefore, we also optimized a plausible coordination isomer structure, giving **DFT-IV** (Supplementary Fig. 9d), in which THF coordinates to Ni(II) at the pseudoapical position, and O(4) is dissociated from Ni(II). **DFT-IV** has no imaginary frequencies. The geometry optimization for a hypothetical symmetric structure gave **DFT-V** (Supplementary Fig. 9e), which possesses a large imaginary frequency (–418 cm^{-1}), likely due to symmetric restriction in the geometry optimization process.

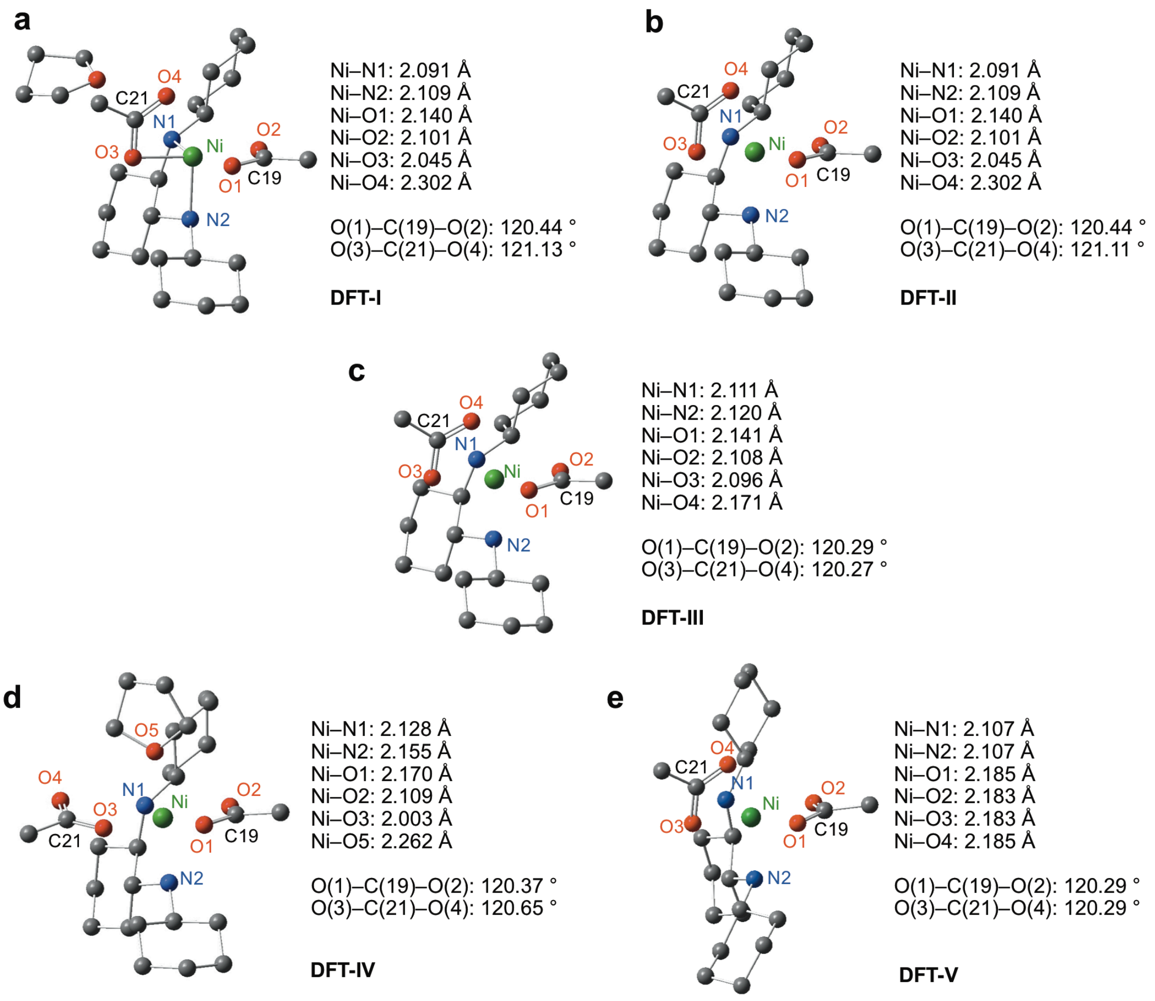

Supplementary Figure 9 | DFT-optimized structures. (**a**) The structure of **DFT-I** (with THF). (**b**) The structure of **DFT-II** (without THF). (**c**) The structure of **DFT-III** (without THF). (**d**) The structure of **DFT-IV**. (**f**) The structure of **DFT-V**. For **b**–**f**, all hydrogen atoms are omitted for the sake of clarity. For **DFT-II** and **DFT-III**, the longer Ni–N bond is depicted at the pseudoapical position. For **DFT-I** and **DFT-II**, the ModRedundant option was used for fixing Ni, C, O and N and only hydrogen atoms were optimized.

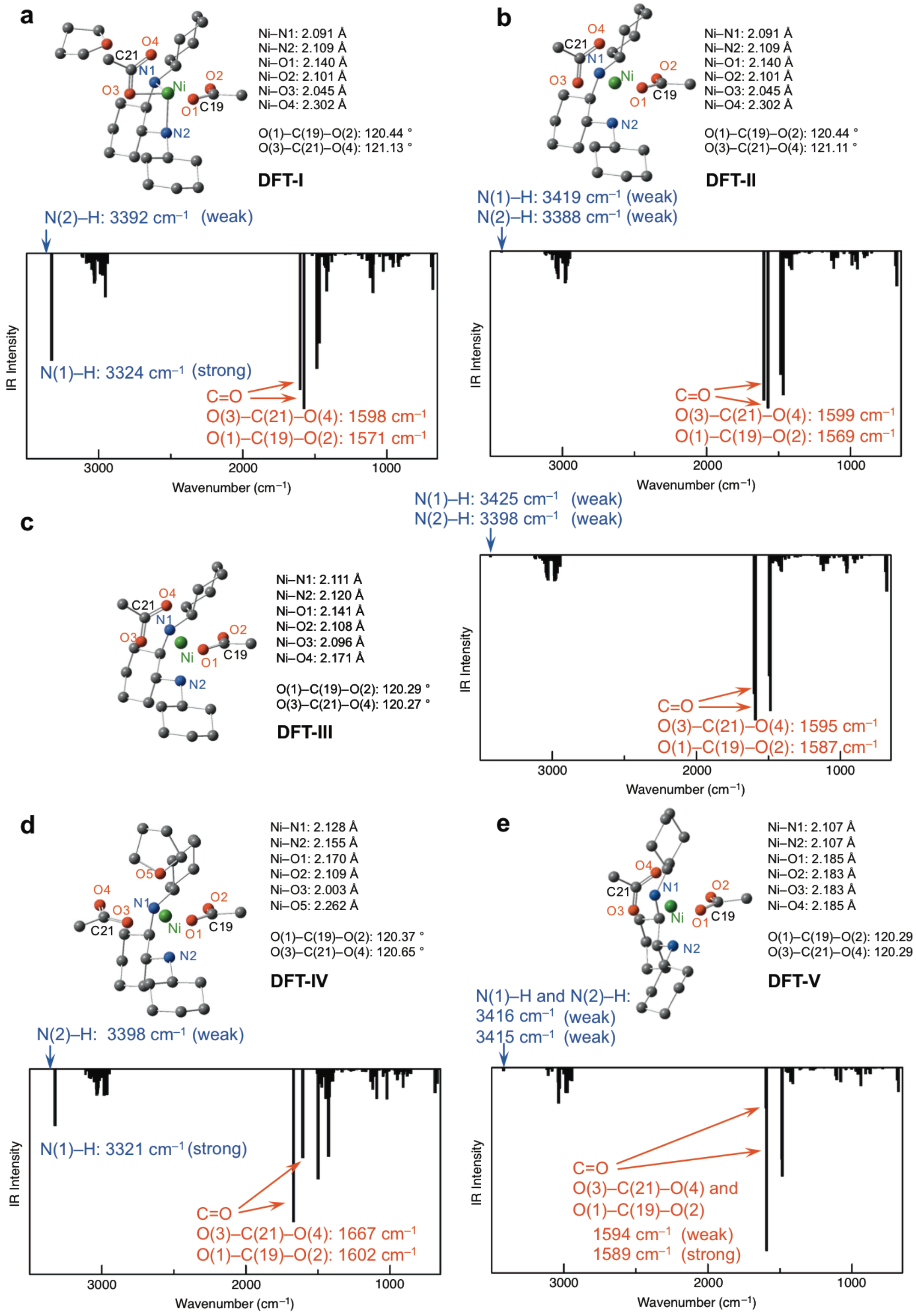

Supplementary Figure 10 | Simulated IR spectra computed at the UM06/6-311g(d,p) (SDD for Ni) level of theory. (**a**) for **DFT-I**. (**b**) for **DFT-II**. (**c**) for **DFT-III**. (**d**) for **DFT-IV**. (**e**) for **DFT-V**. For (**a**)–(**e**), all the calculated vibrational frequencies were scaled by the reported scaling factor (0.989)[12].

Characteristic features experimentally observed in the IR spectrum of complex **I** (Fig. 4a in the main text) are the N–H (3225 cm^{-1})[13] and the carboxylate (598 and 1558 cm^{-1}) band[14]. Among the structures we examined, the simulated IR spectrum of **DFT-I** with the coordinating THF, in which the ModRedundant option was used for geometry optimization, gave the best fit [Supplementary Fig. 10 (a), N(1)−H: 3324 cm^{-1}, O(3)–C(21)–O(4): 1598 and O(1)–C(19)–O(2): 1571 cm^{-1}] with the corresponding experimental IR spectrum of complex **I** (Fig. 4a, main text). In the case of **DFT-II** [Supplementary Fig. 10 (b)] without the coordinating THF, the peaks of the carboxylates [O(3)–C(21)–O(4): 1599 and O(1)–C(19)–O(2): 1569 cm^{-1}] were found to be in good agreement with the experimental results, but the N–H peak became significantly weak due to the lack of H-bonding. For **DFT-III** [Supplementary Fig. 10 (c)], in which the bond length of Ni–O(4) (2.171 Å) was shorter than the observed value [2.302(2) Å], the differences of the peaks of two carboxylates became smaller [O(3)–C(21)–O(4): 1595 and O(1)–C(19)–O(2): 1586 cm^{-1}]. In the case of a plausible coordination isomer **DFT-IV**, in which THF coordinates to Ni(II) at the pseudoapical position, and O(4) is dissociated from Ni(II), the simulated IR [Supplementary Fig. 10 (d)] gave analogous carboxylate peaks [O(3)–C(21)–O(4): 1666 cm^{-1} and O(1)–C(19)–O(2):1602 cm^{-1}]. These values are significantly higher than those in the observed IR spectrum of complex **I** in THF[14]. The simulated IR spectrum for symmetric **DFT-V** [Supplementary Fig. 10 (e)] gave peaks for both N–H and carboxylates that were different from the observed peaks.

These computational investigations focusing on IR spectra suggest that Ni(II)–diamine–acetates complex **I** in THF should have a pseudo-octahedral structure, in which two structurally distinct acetates coordinate to the Ni(II) center in a bidentate fashion. The N–H functionality in complex **I** should coordinate to THF.

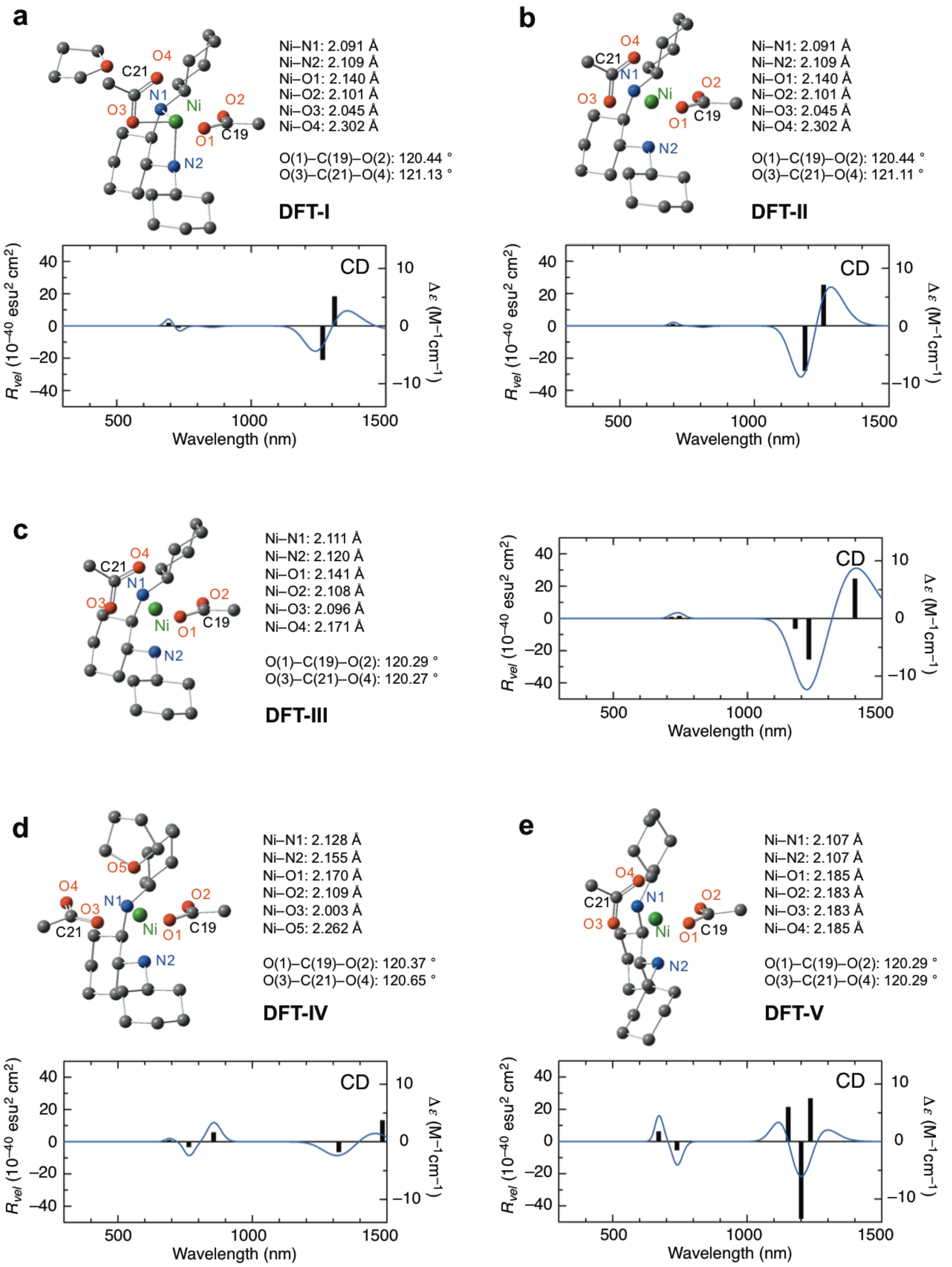

Supplementary Figure 11 | Simulated ECD spectra computed at the UM06/6-311g(d,p) (SDD for Ni) level of theory. The simulated ECD spectra (σ = 0.05 eV). (**a**) for **DFT-I**. (**b**) for **DFT-II**. (**c**) for **DFT-III**. (**d**) for **DFT-IV**. (**e**) for **DFT-V**.

The simulated ECD spectra for **DFT-I**, **DFT-II** and **DFT-III** (Supplementary Fig. 11 a, b and c) reasonably fit the features of the observed ECD spectrum of complex **I** measured in THF (Fig. 4b in the main text). The intensities of the near-IR bands are significantly stronger than that for visible absorption. In the case of the simulated ECD

spectra for **DFT-IV** (Supplementary Fig. 11d) and **DFT-V** (Supplementary Fig. 11e), the ECD intensity in the near-IR bands became weaker. The patterns of the spectra of **DFT-IV** (Supplementary Fig. 11d) and **DFT-V** (Supplementary Fig. 11e) were also significantly different from the observed ECD spectrum of complex **I** measured in THF (Fig. 4b in the main text).

These results support the idea that the (*Λ*)-chirality in Ni(II)–diamine–acetate **I** determined by X-ray is substitutionally and configurationally inert in THF.

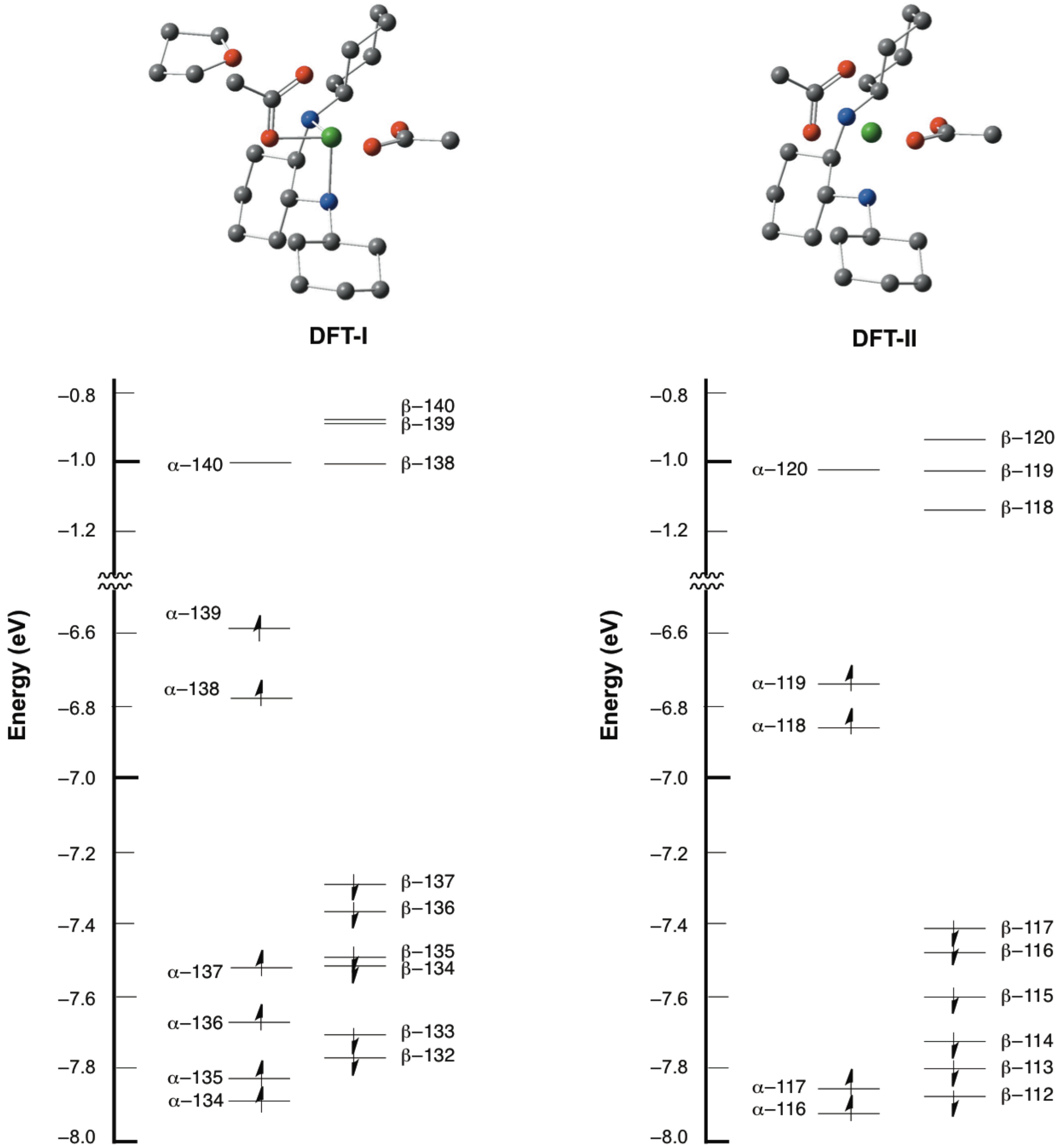

Supplementary Figure 12 | Energy diagrams of mononuclear DFT-I and DFT-II computed at the UM06/6-311g(d,p) (SDD for Ni) level of theory.

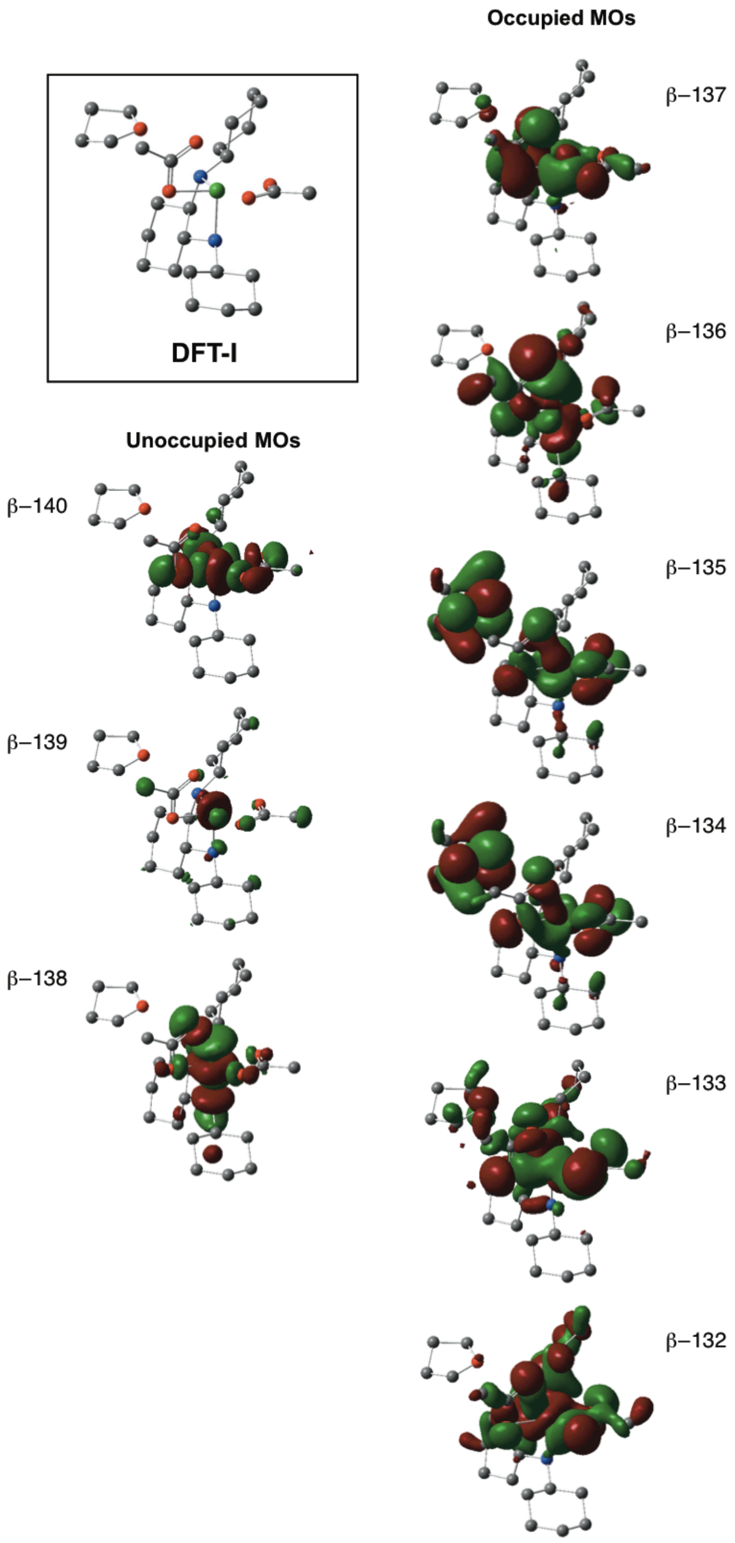

Supplementary Figure 13 | Kohn–Sham *beta* orbitals of DFT-I computed at the UM06/6-311g(d,p) (SDD for Ni) level of theory. The molecular orbitals were visualized using an isosurface value of 0.02 on the optimized structure.

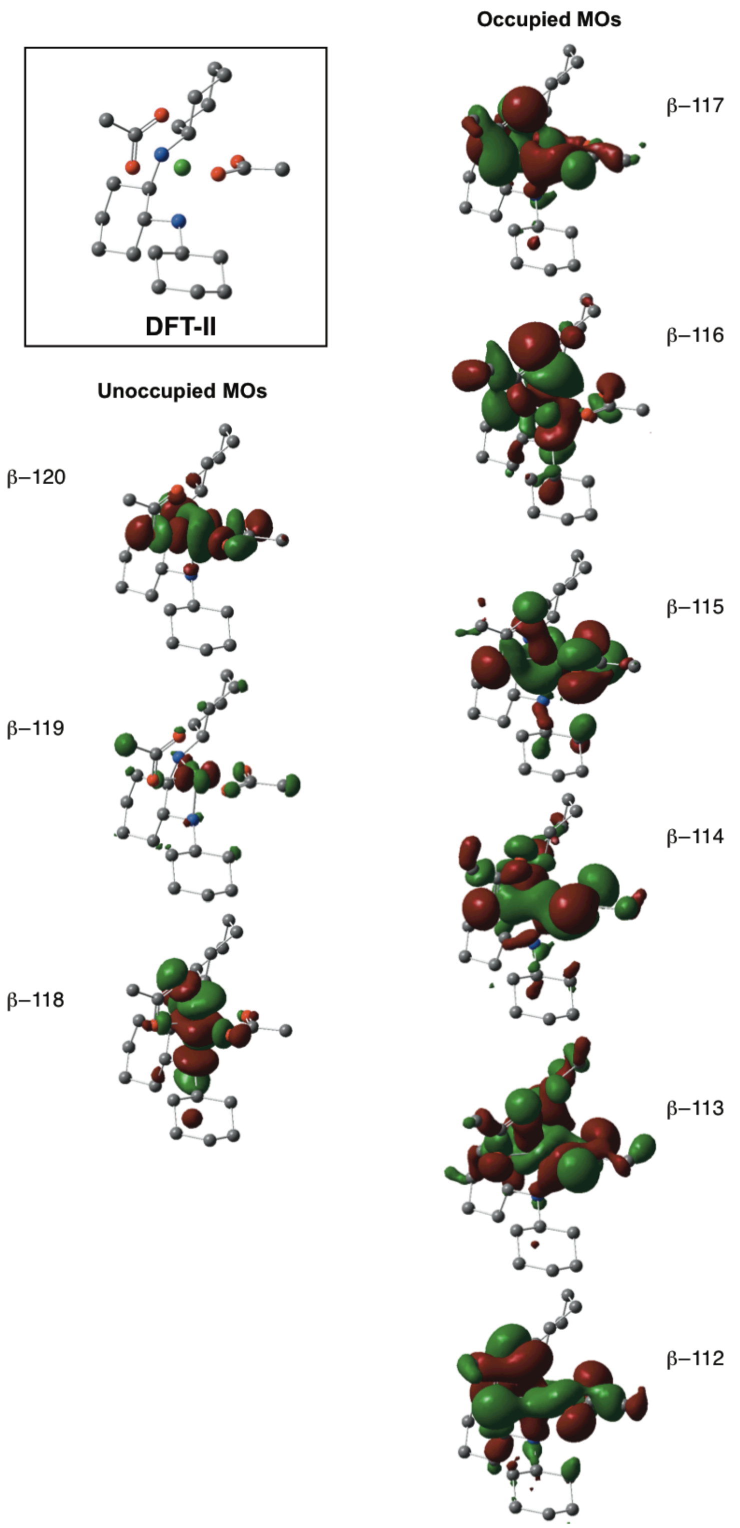

Supplementary Figure 14 | Kohn–Sham *beta* orbitals of DFT-IV computed at the UM06/6-311g(d,p) (SDD for Ni) level of theory. The molecular orbitals were visualized using an isosurface value of 0.02 on the optimized structure.

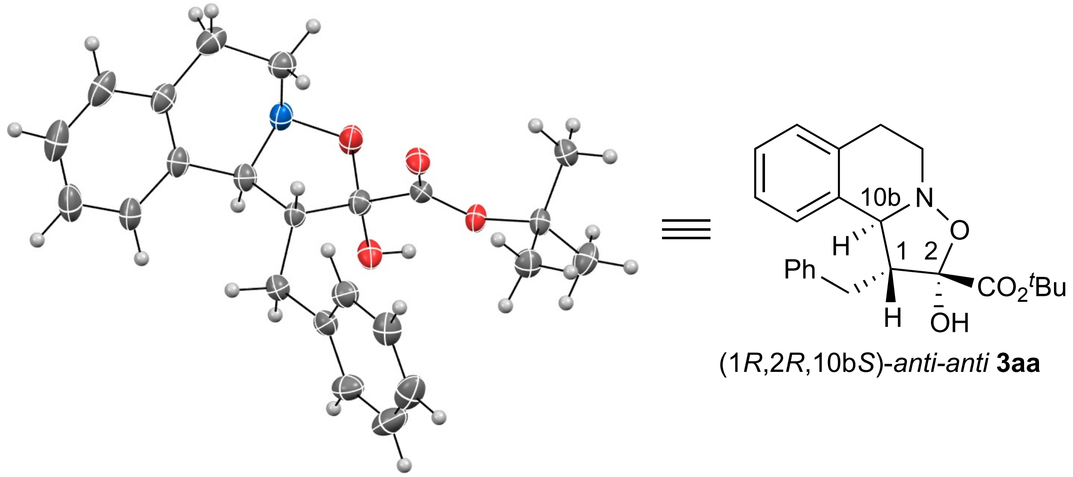

Supplementary Figure 15 | The structure of (1*R*,2*R*,10b*S*)-*anti-anti*-3aa (CCDC 1482737) determined by X-ray analysis.

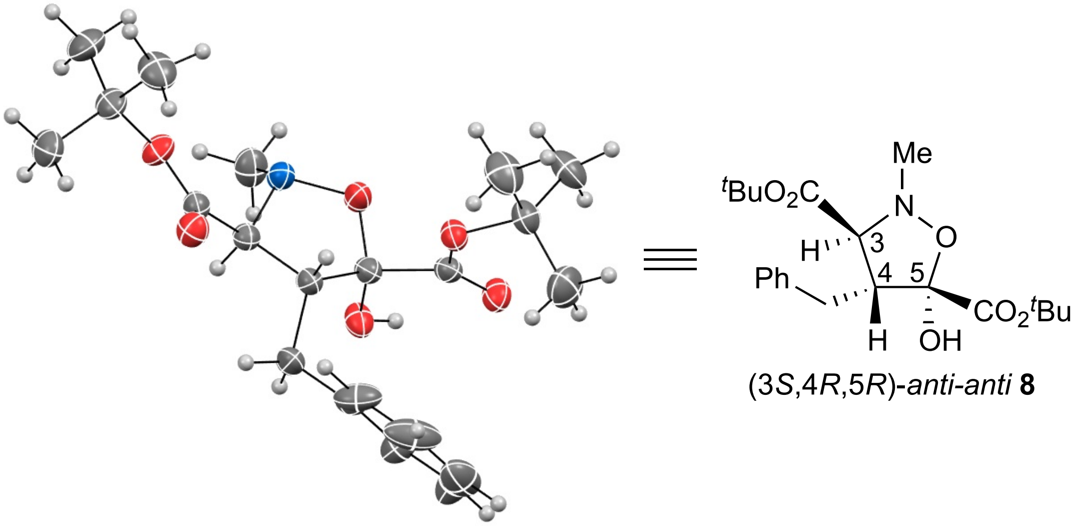

Supplementary Figure 16 | The structure of (3*S*,4*R*,5*R*)-*anti-anti*-8 (CCDC 1482738) determined by X-ray analysis. Disorder is neglected for the sake of clarity.

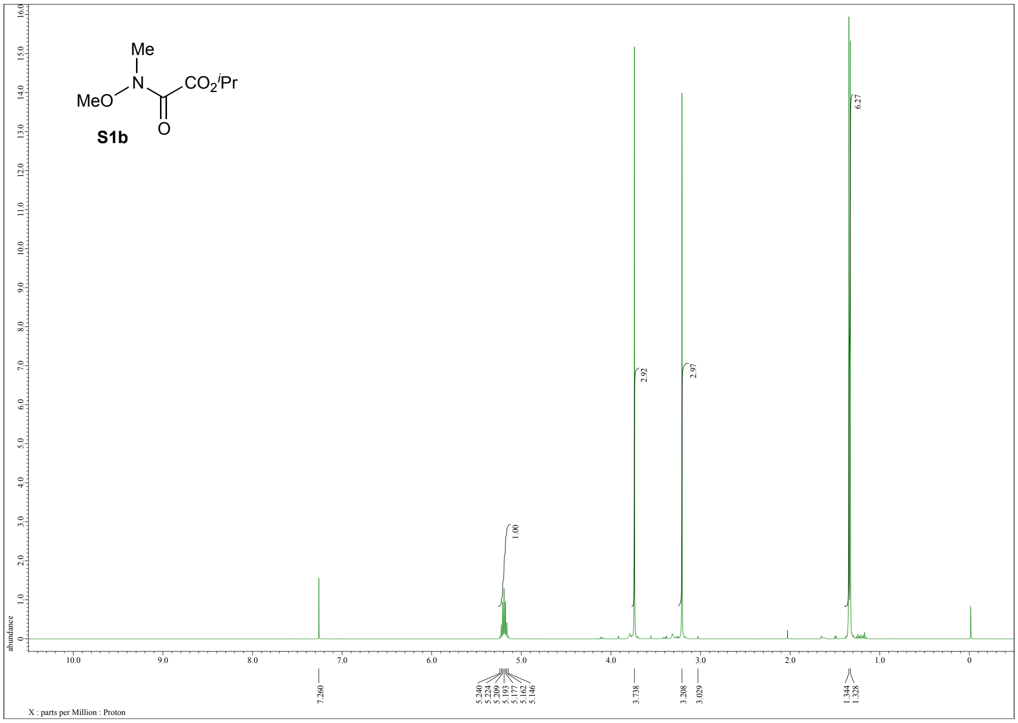

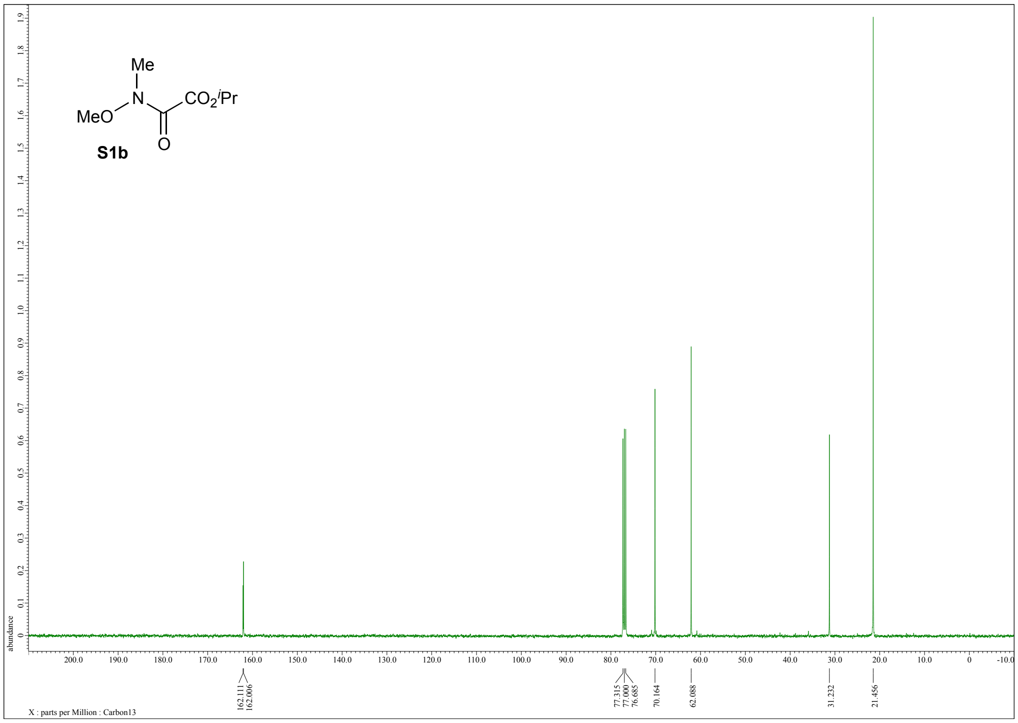

Supplementary Figure 17 | ^{1}H and ^{13}C NMR spectra of S1b.

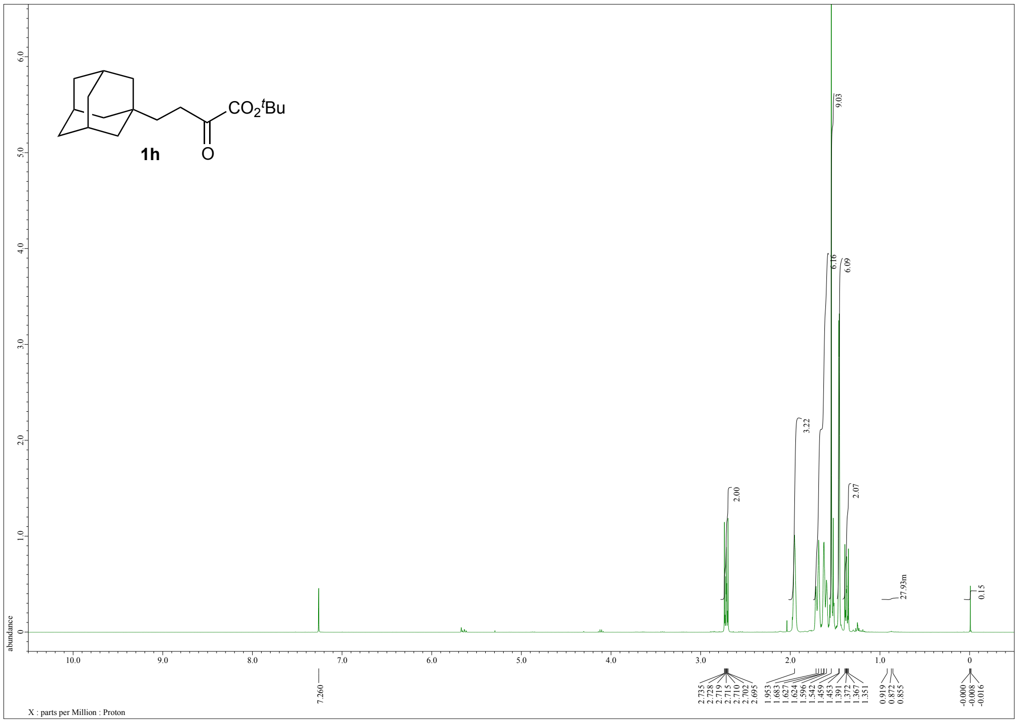

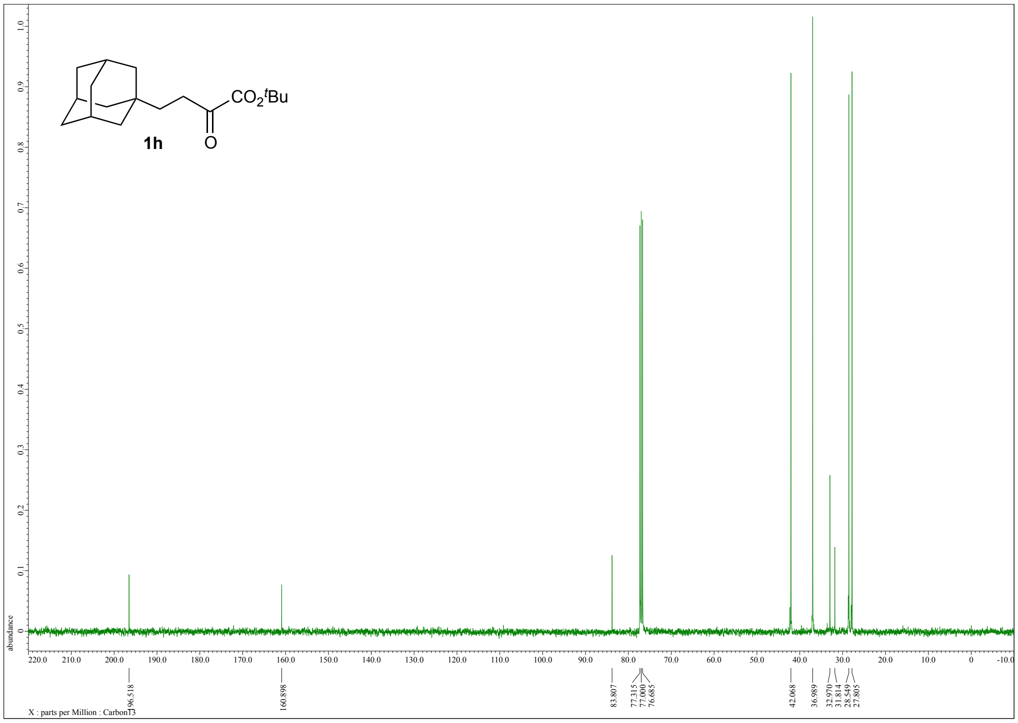

Supplementary Figure 18 | ^{1}H and ^{13}C NMR spectra of 1h.

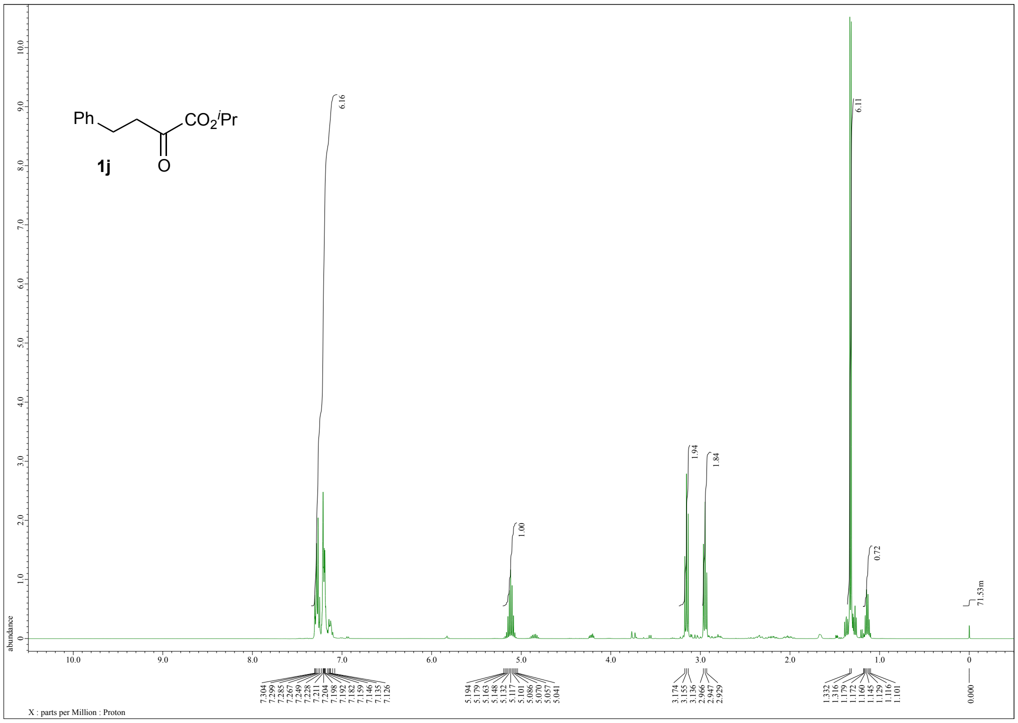

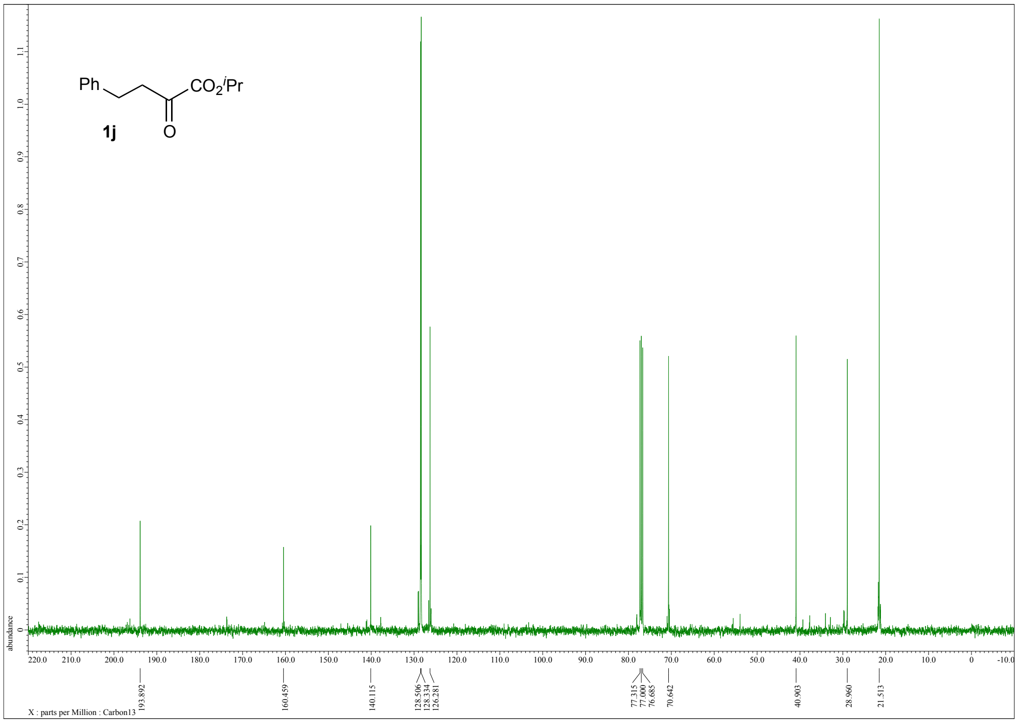

Supplementary Figure 19 | ^{1}H and ^{13}C NMR spectra of 1j.

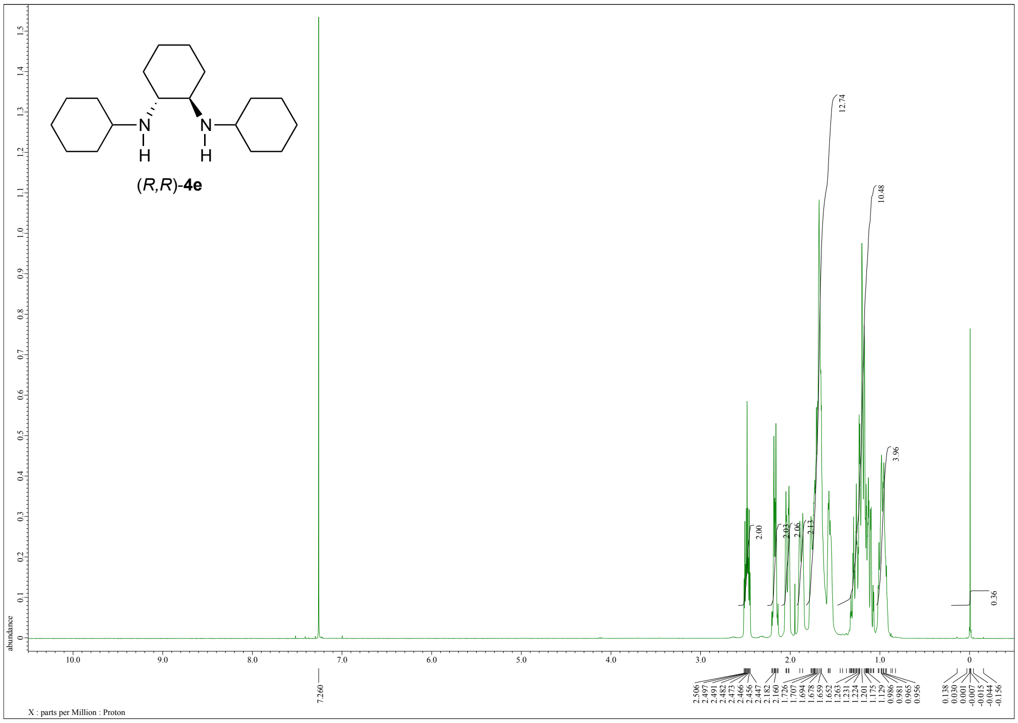

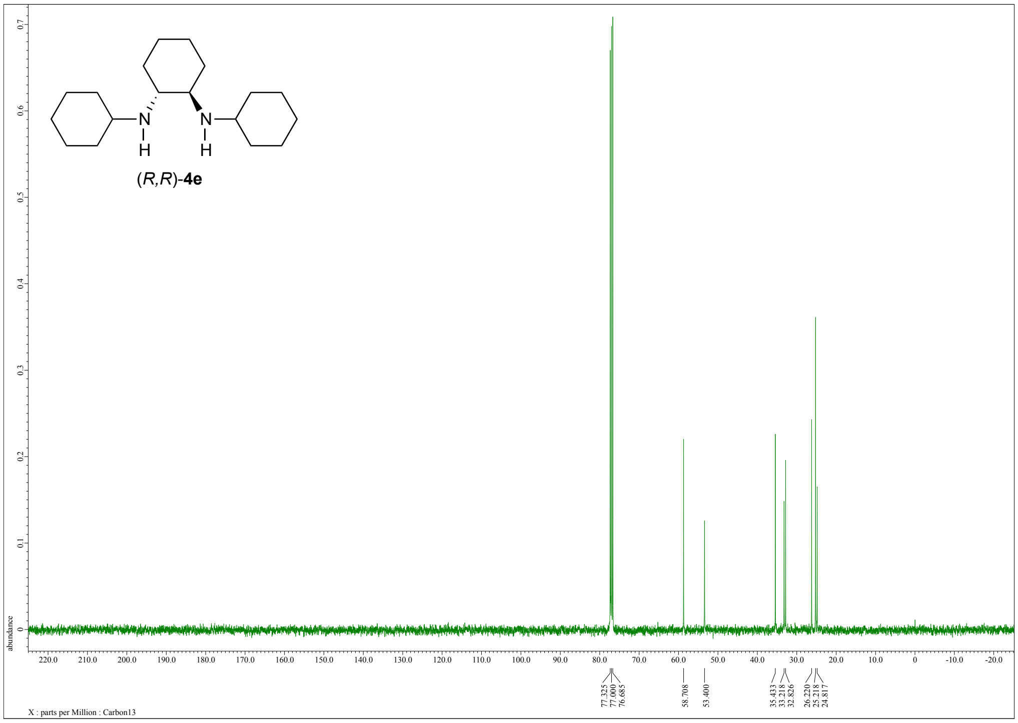

Supplementary Figure 20 | ^{1}H and ^{13}C NMR spectra of (*R*,*R*)-4e.

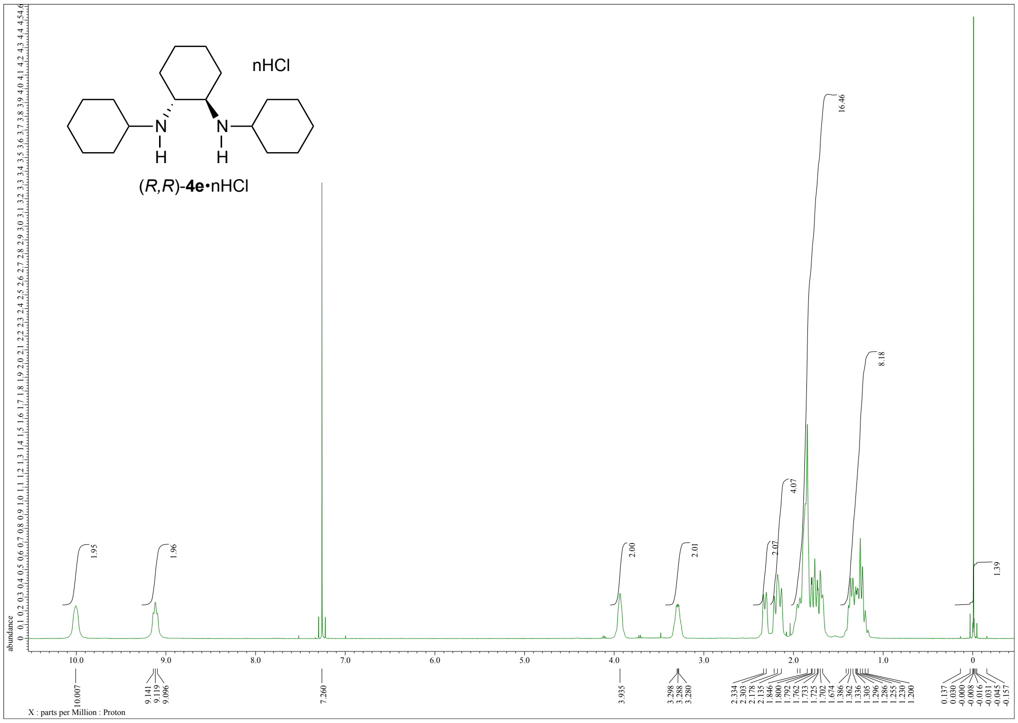

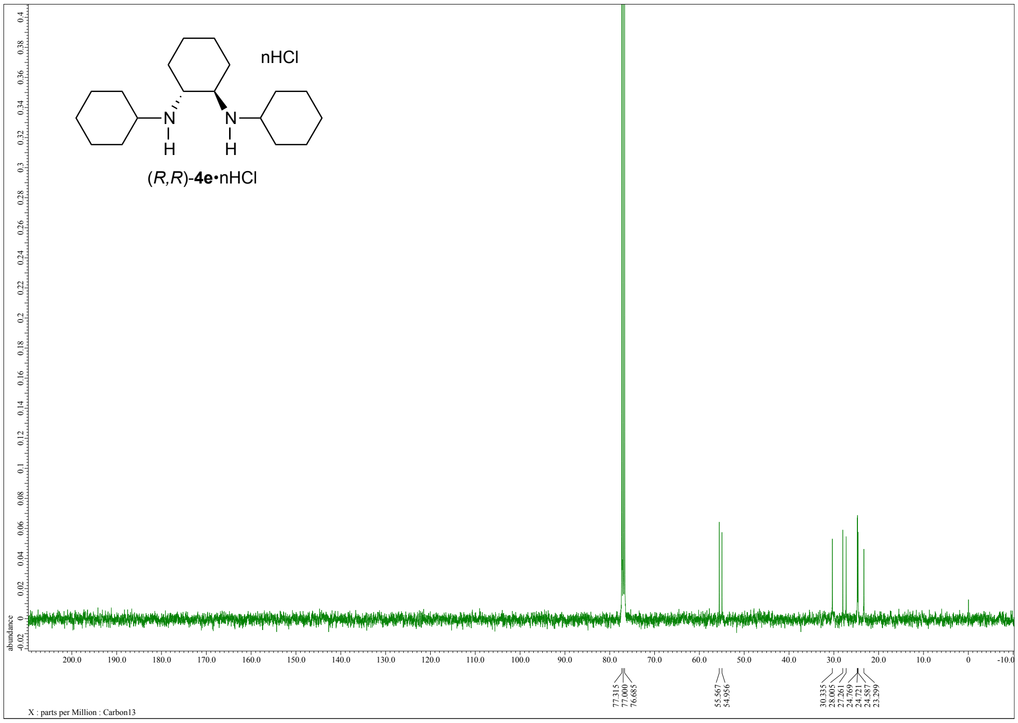

Supplementary Figure 21 | ^{1}H and ^{13}C NMR spectra of (*R*,*R*)-4e·nHCl.

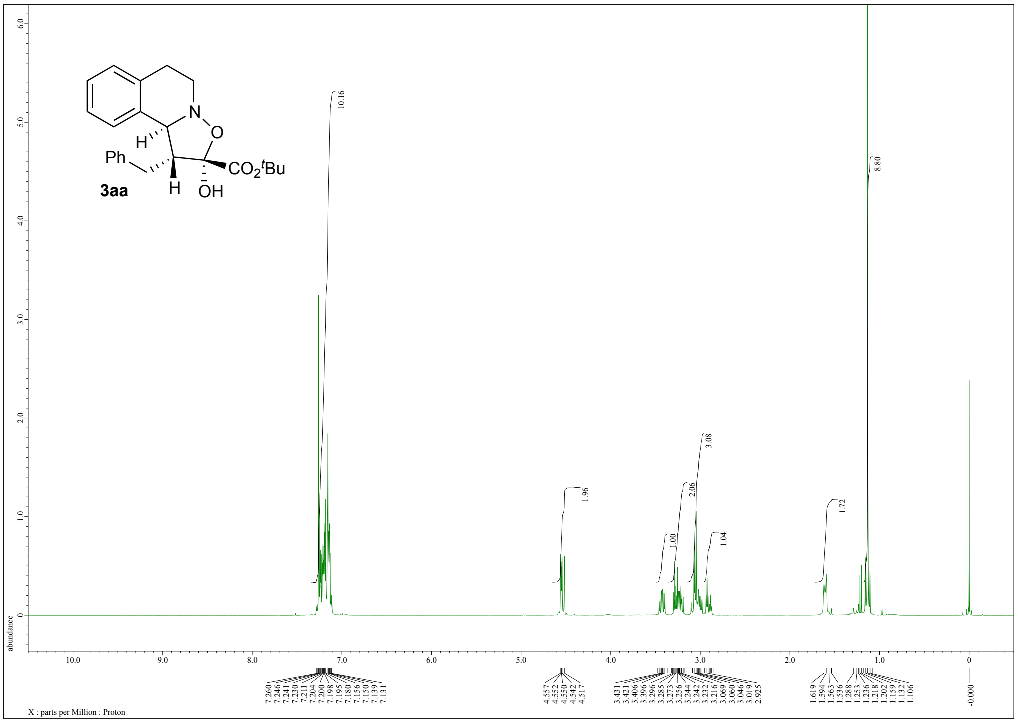

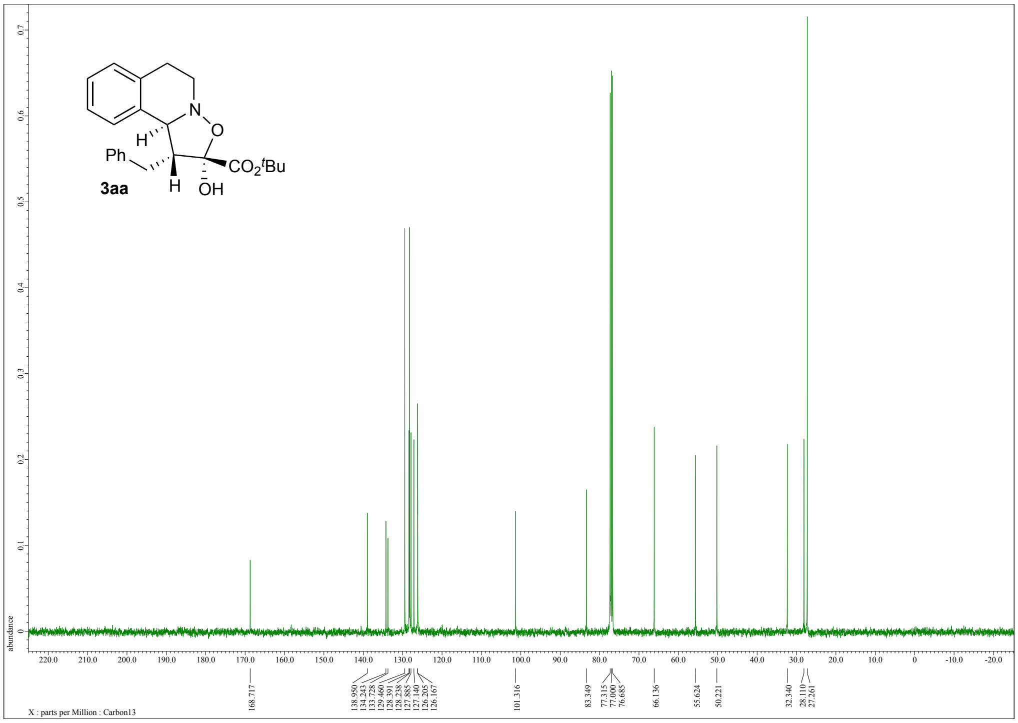

Supplementary Figure 22 | ^{1}H and ^{13}C NMR spectra of 3aa.

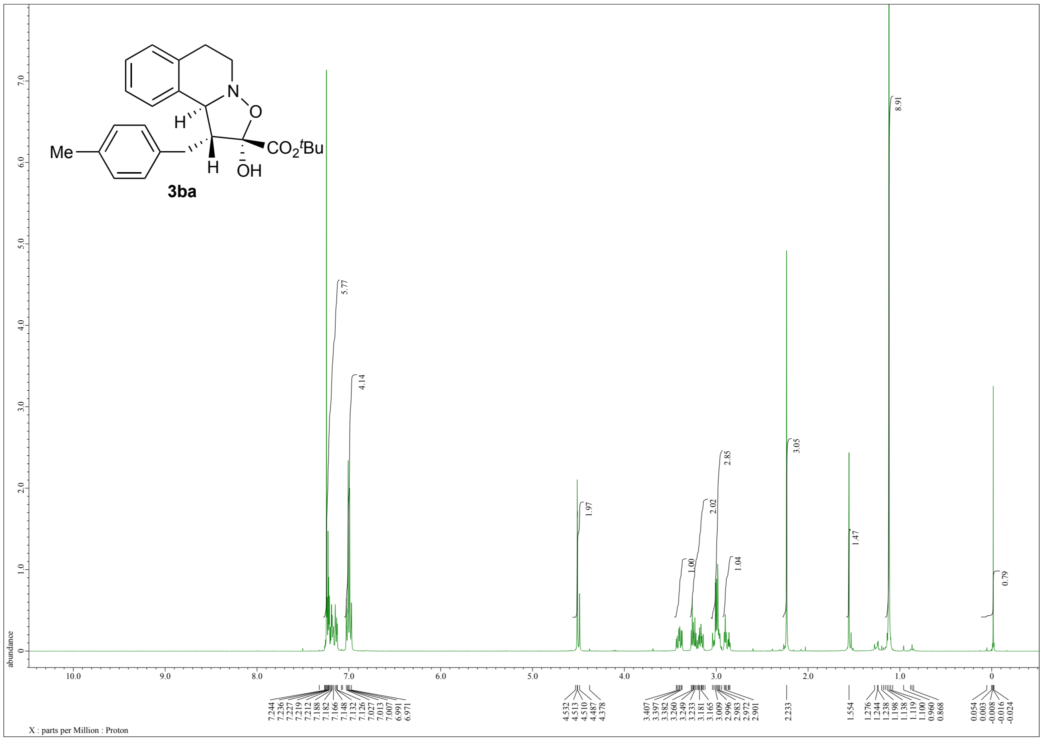

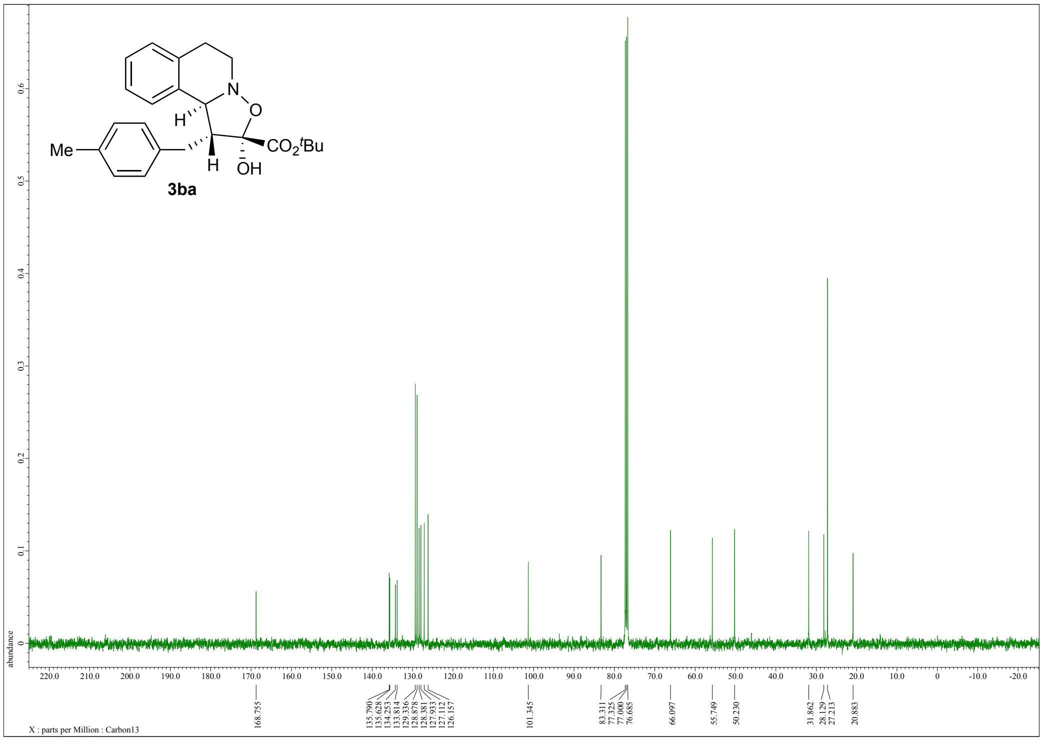

Supplementary Figure 23 | ^{1}H and ^{13}C NMR spectra of 3ba.

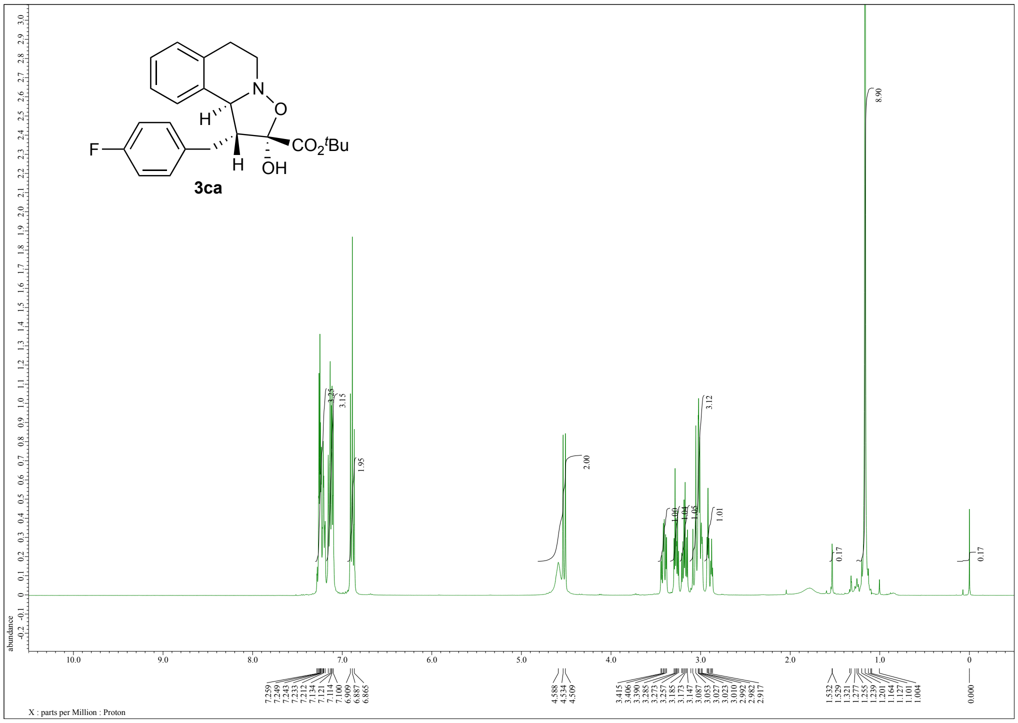

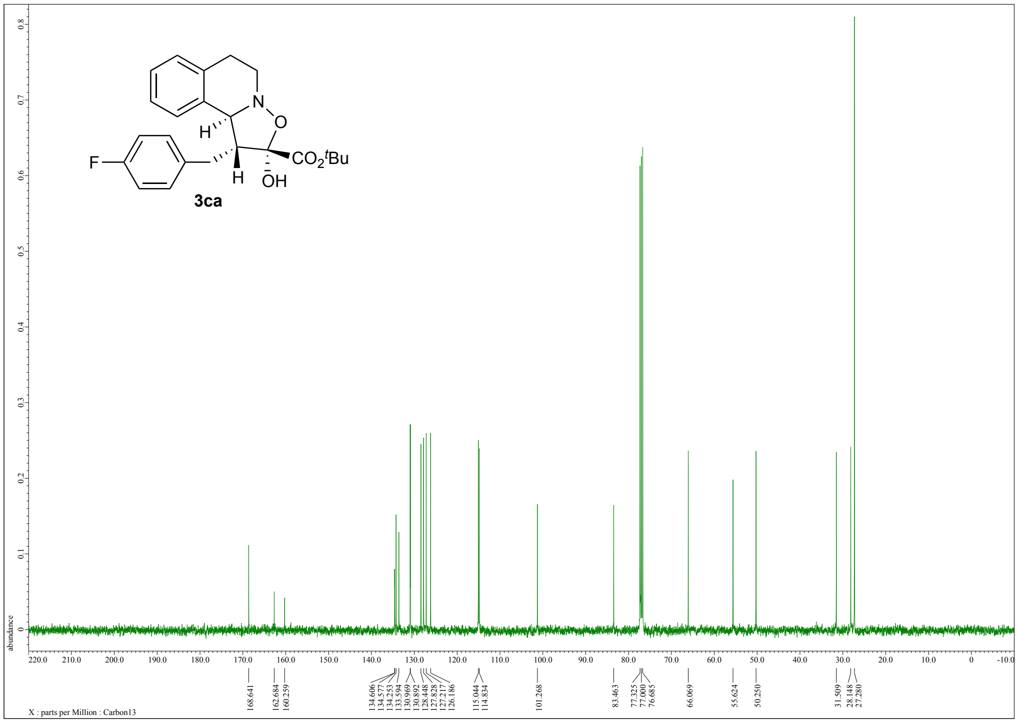

Supplementary Figure 24 | ^{1}H and ^{13}C NMR spectra of 3ca.

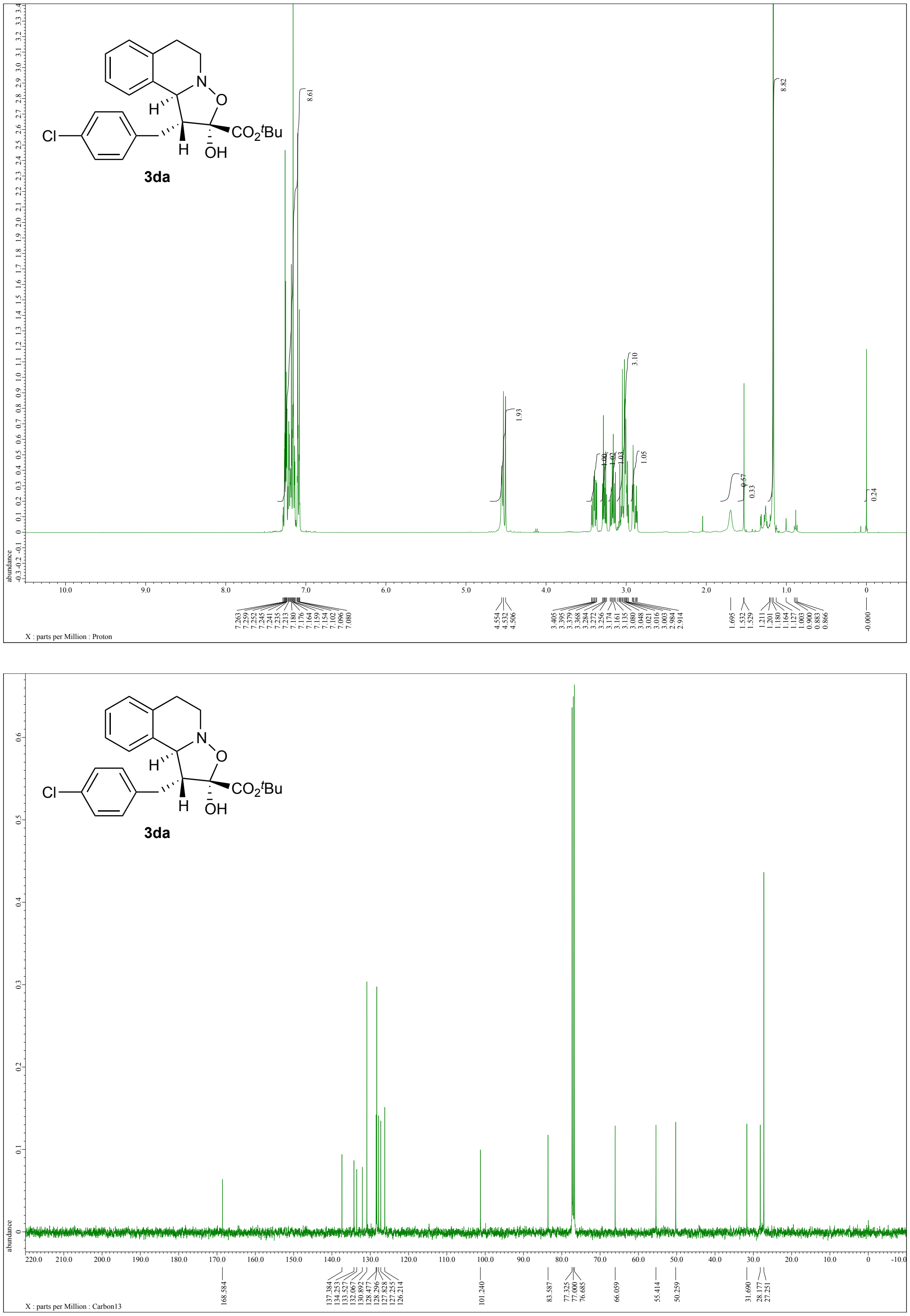

Supplementary Figure 25 | ^{1}H and ^{13}C NMR spectra of 3da.

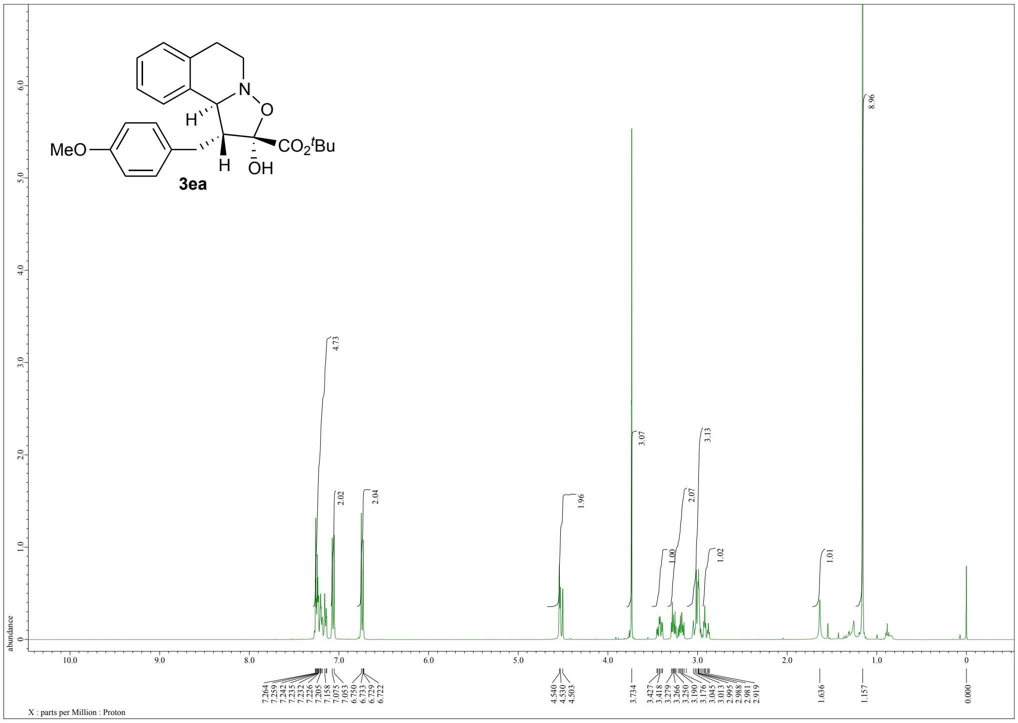

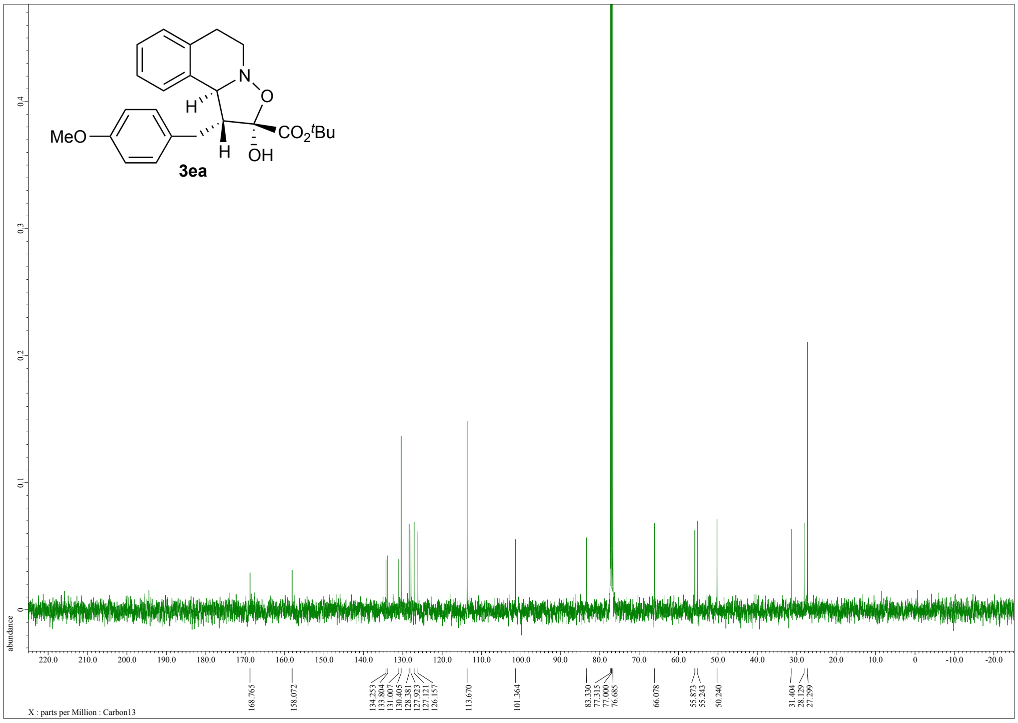

Supplementary Figure 26 | ^{1}H and ^{13}C NMR spectra of 3ea.

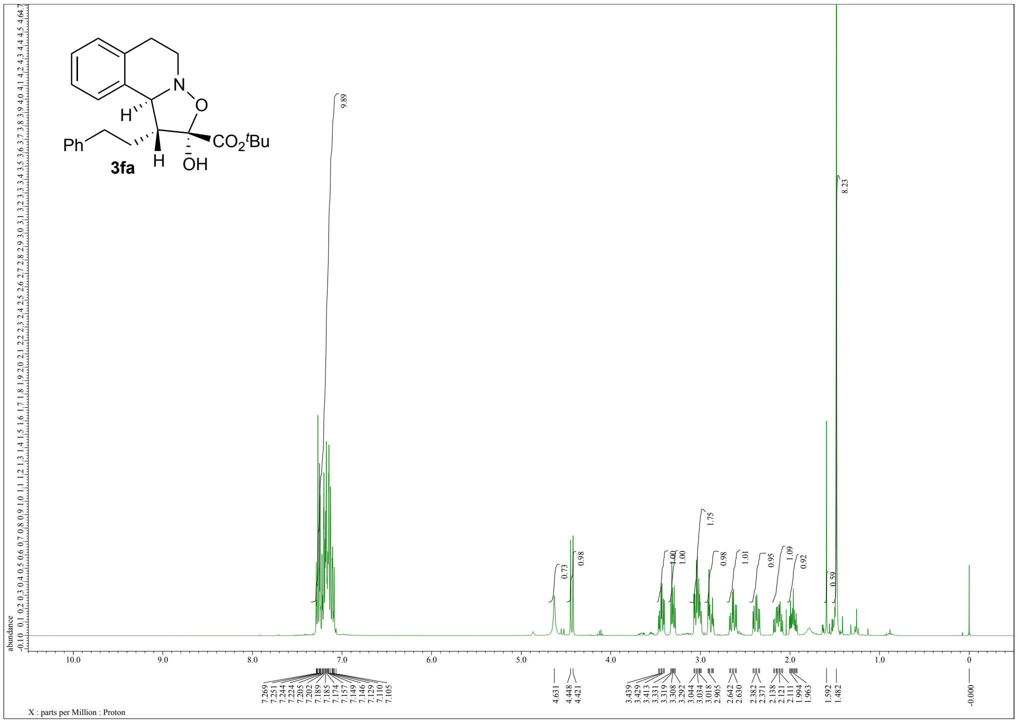

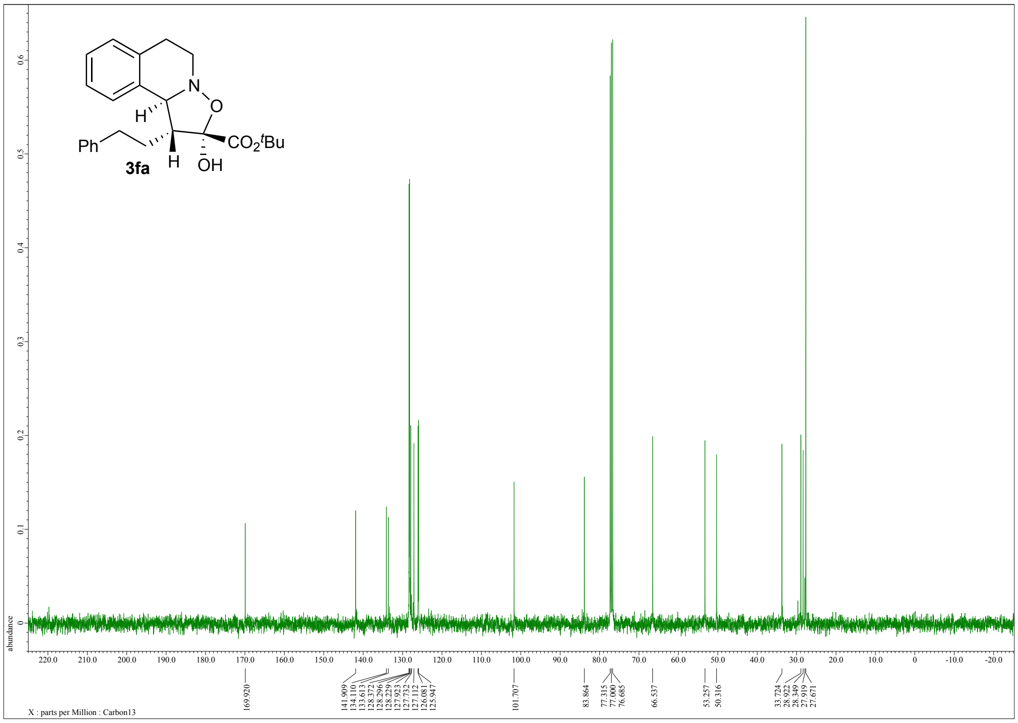

Supplementary Figure 27 | ^{1}H and ^{13}C NMR spectra of 3fa.

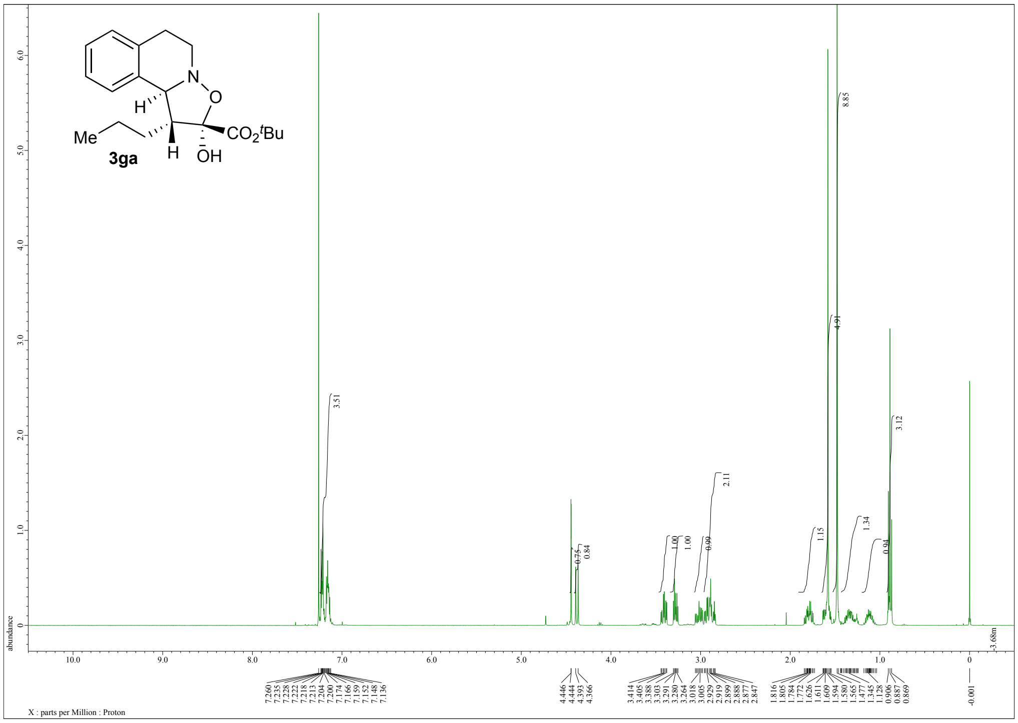

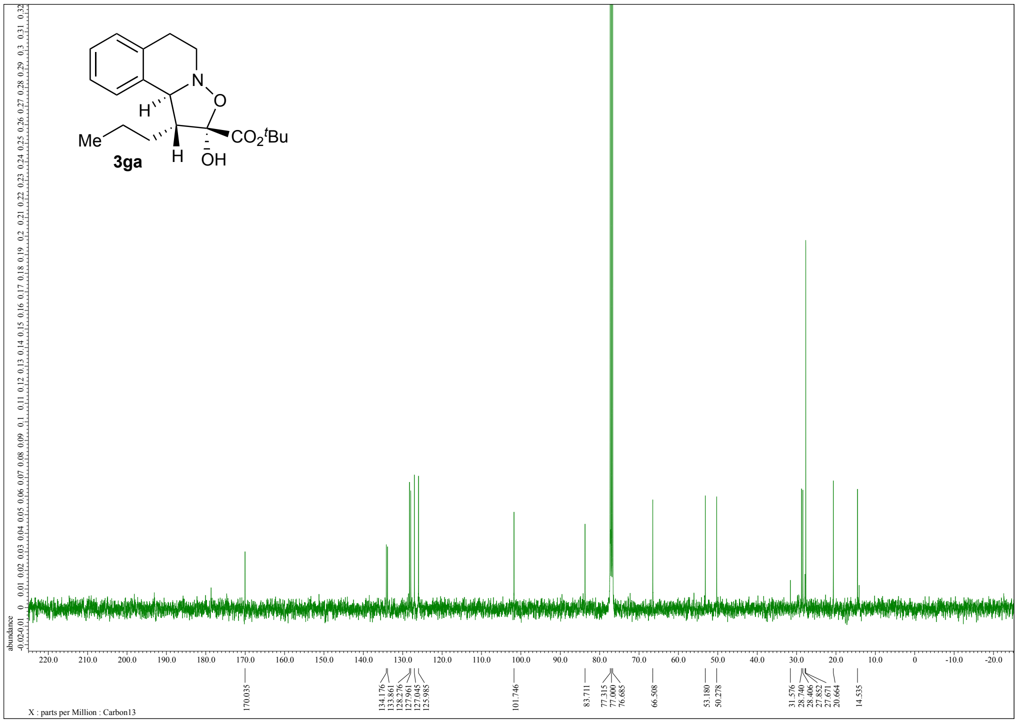

Supplementary Figure 28 | ^{1}H and ^{13}C NMR spectra of 3ga.

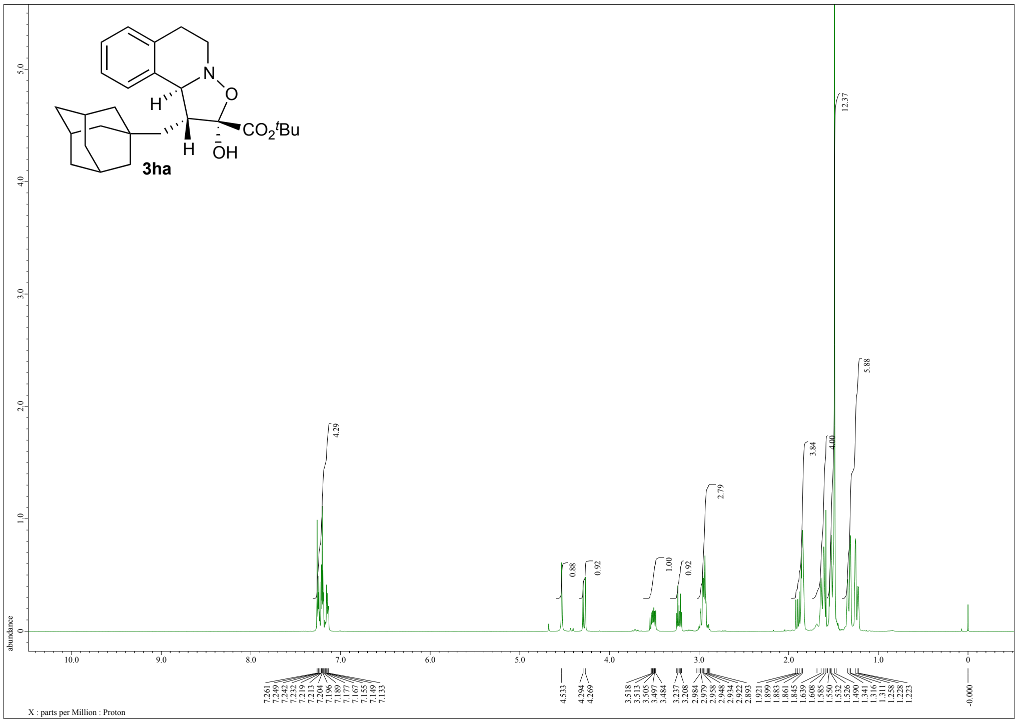

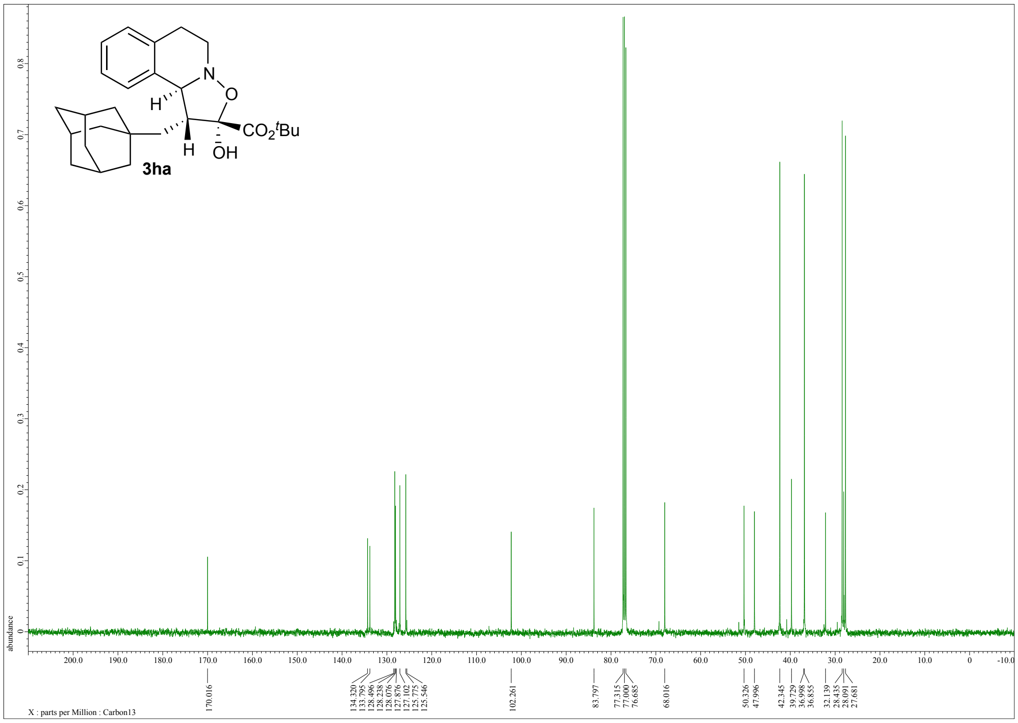

Supplementary Figure 29 | ^{1}H and ^{13}C NMR spectra of 3ha.

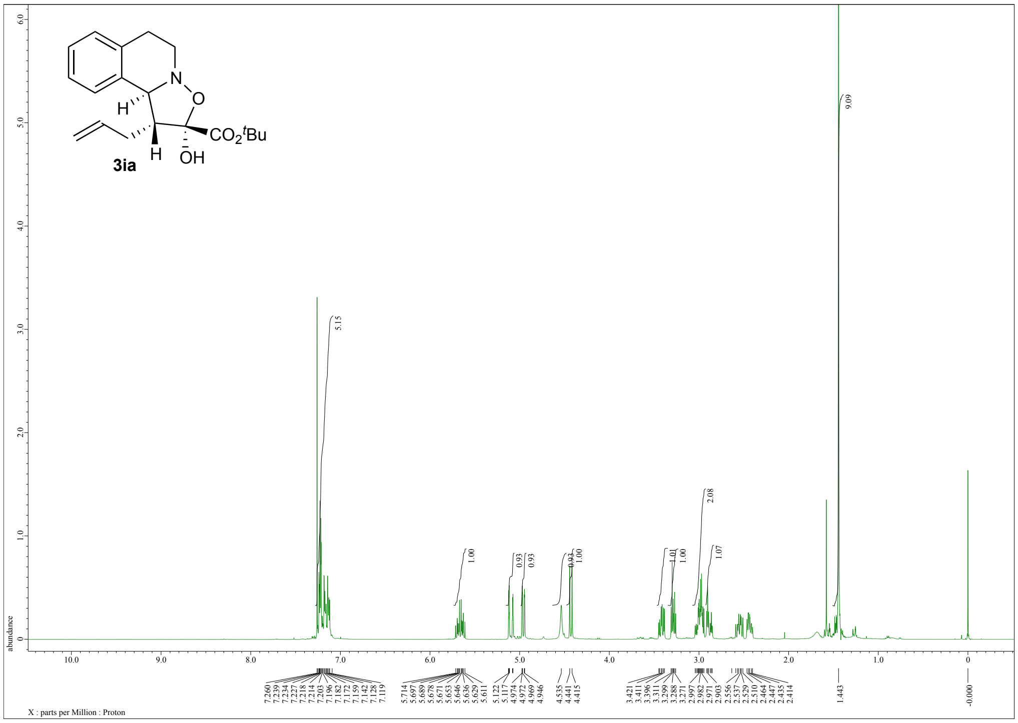

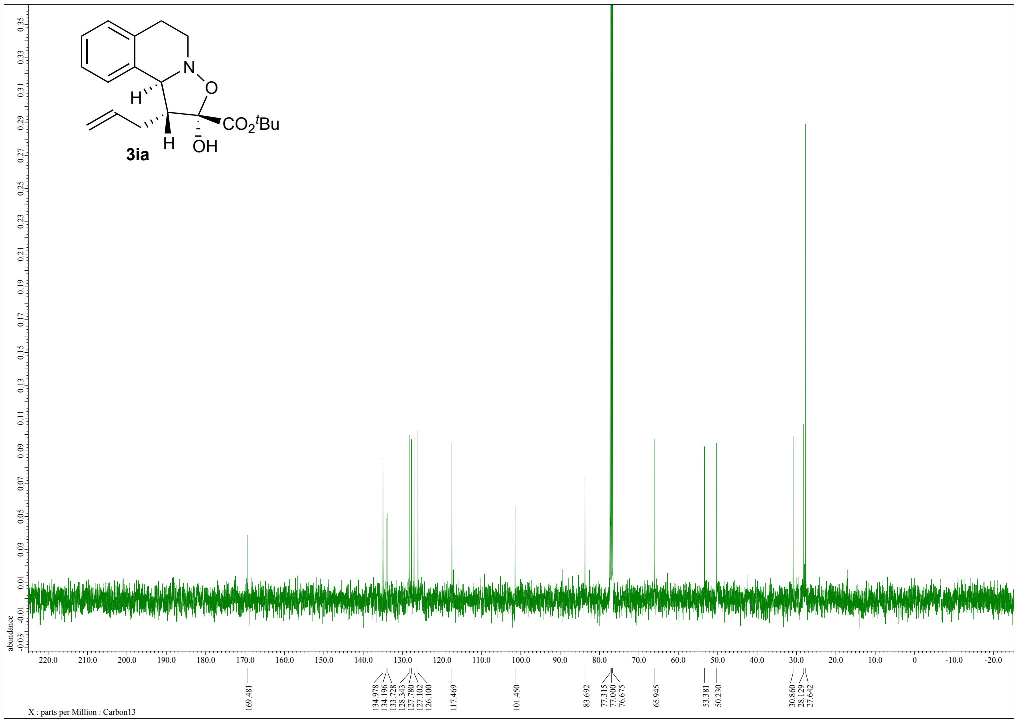

Supplementary Figure 30 | ^{1}H and ^{13}C NMR spectra of 3ia.

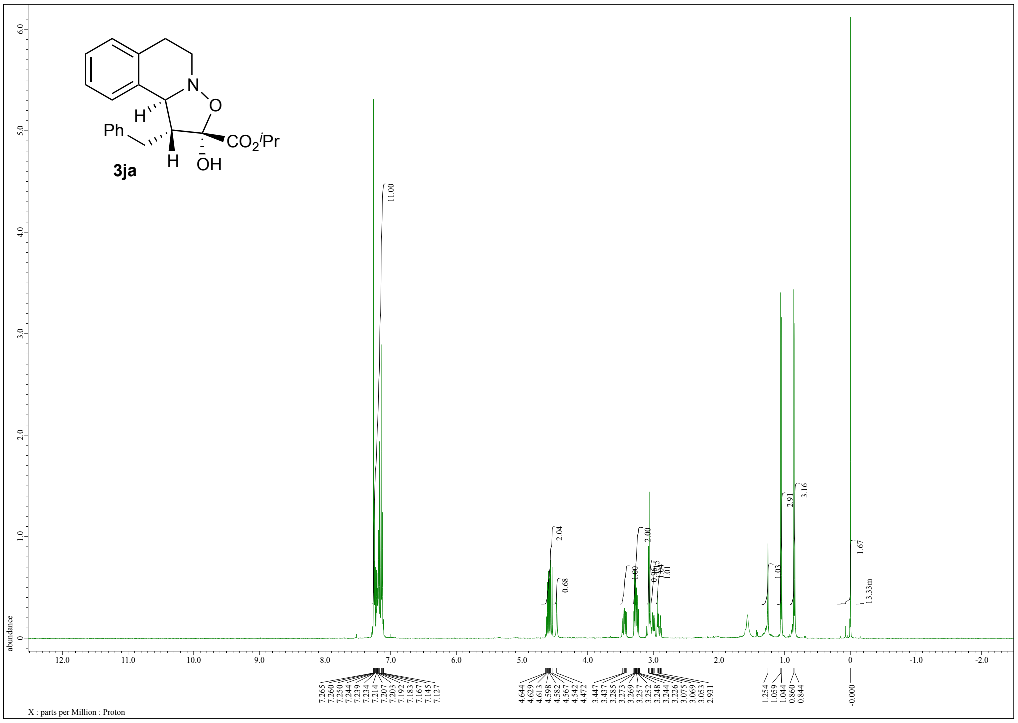

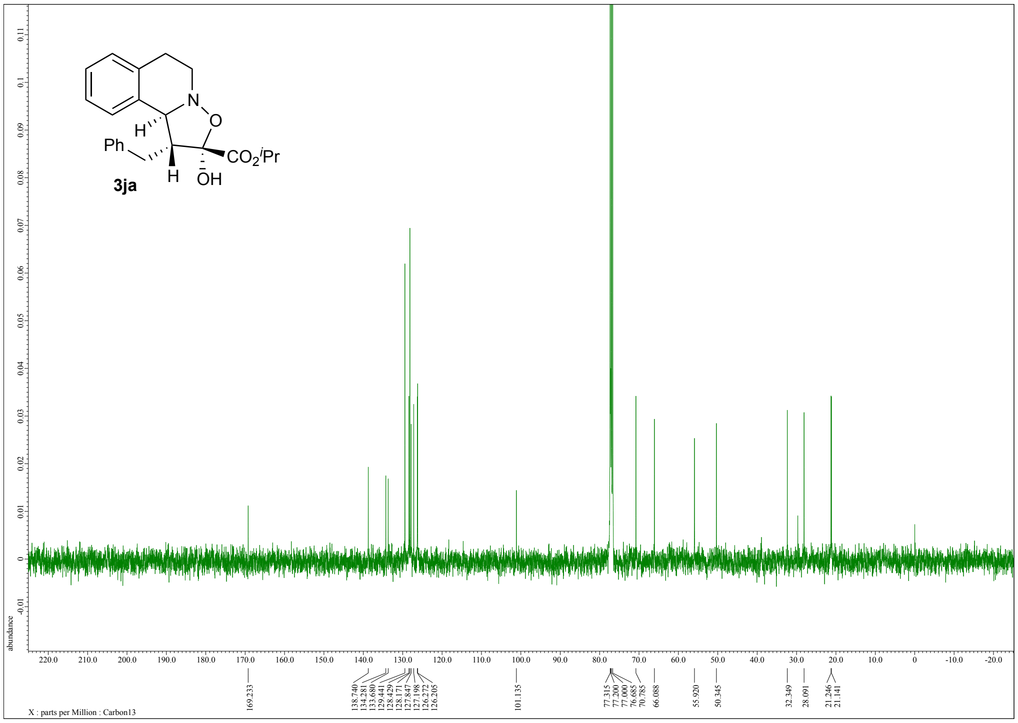

Supplementary Figure 31 | ^{1}H and ^{13}C NMR spectra of 3ja.

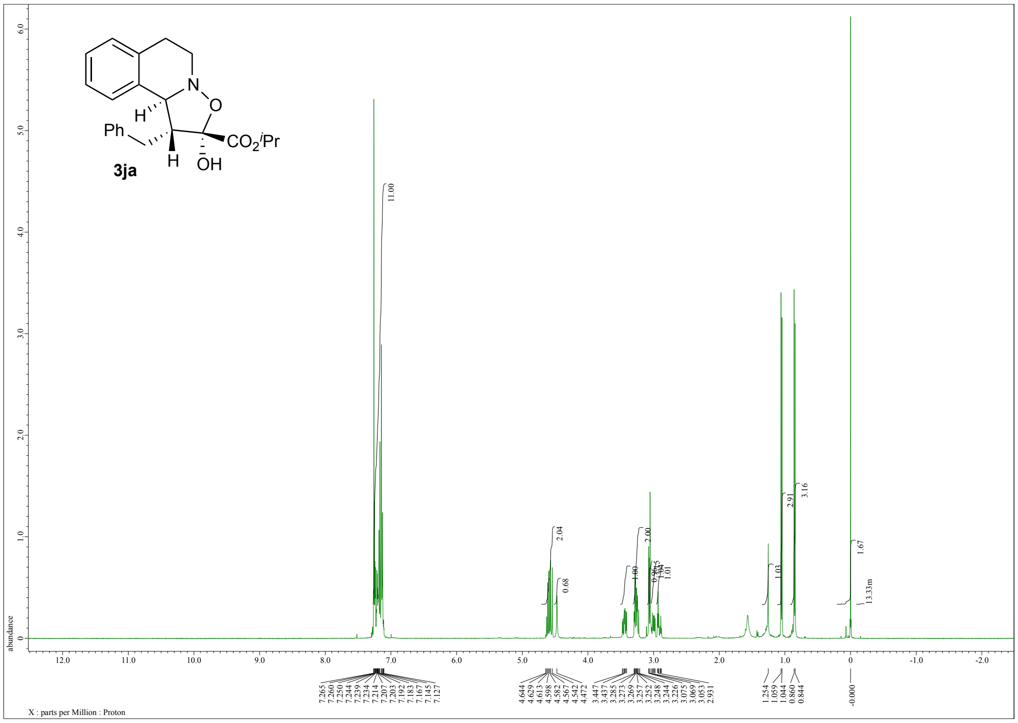

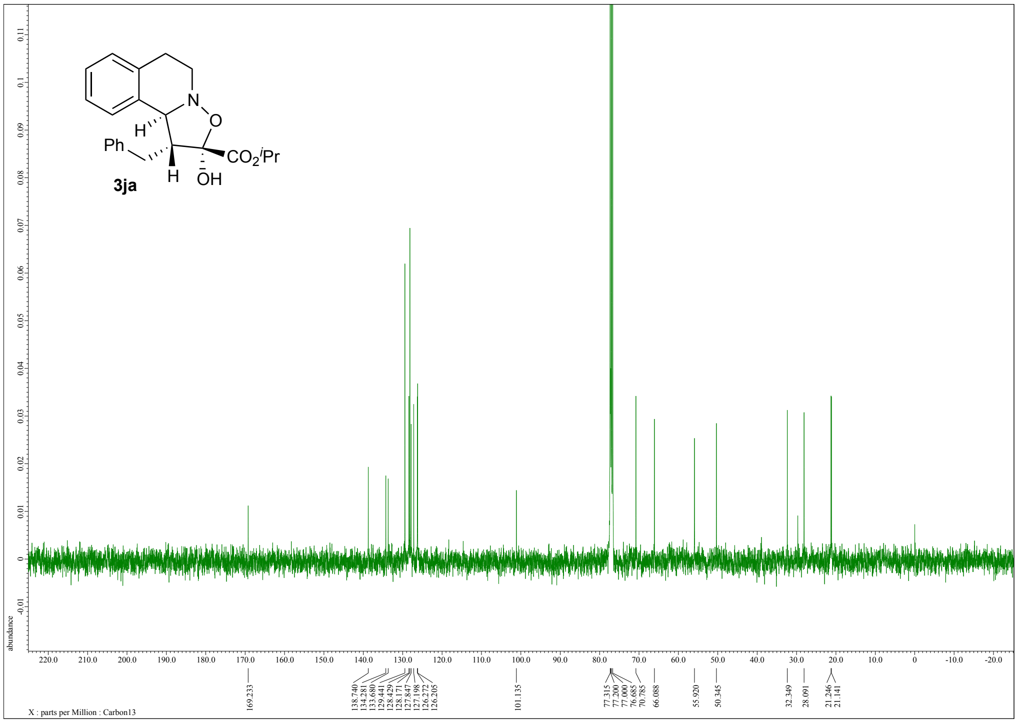

Supplementary Figure 32 | ^{1}H and ^{13}C NMR spectra of 3ja.

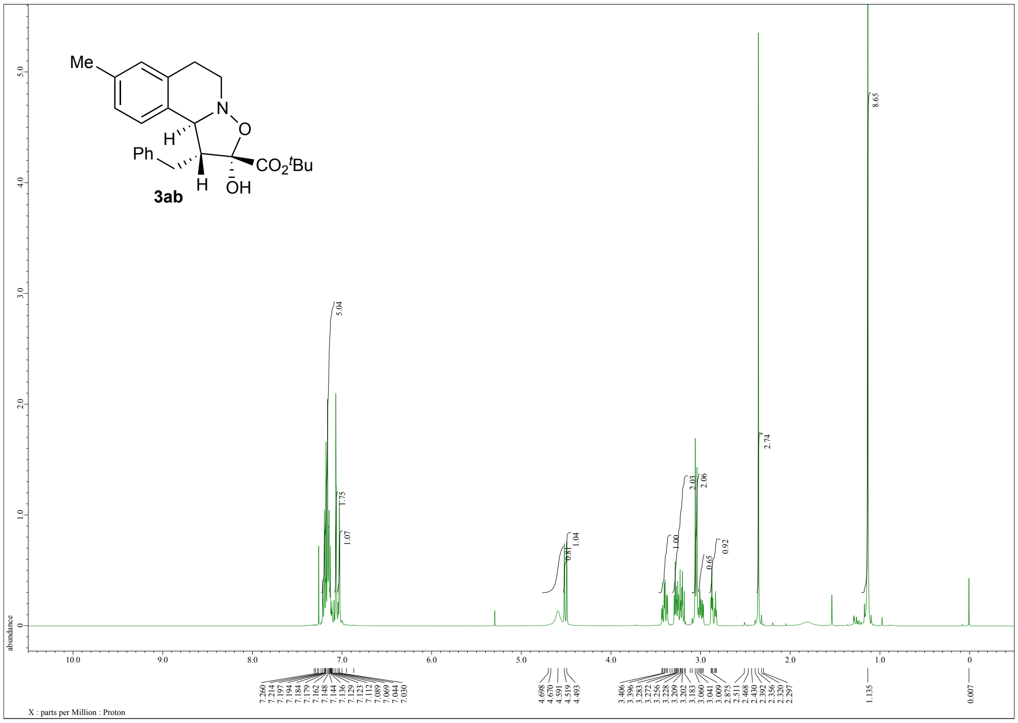

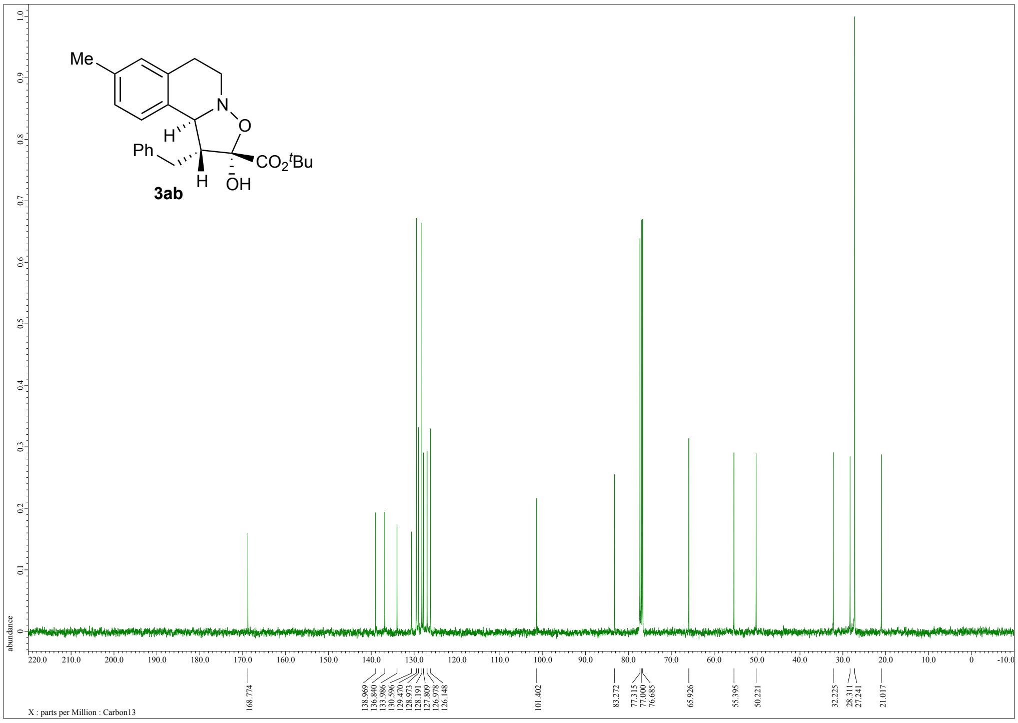

Supplementary Figure 33 | ^{1}H and ^{13}C NMR spectra of 3ab.

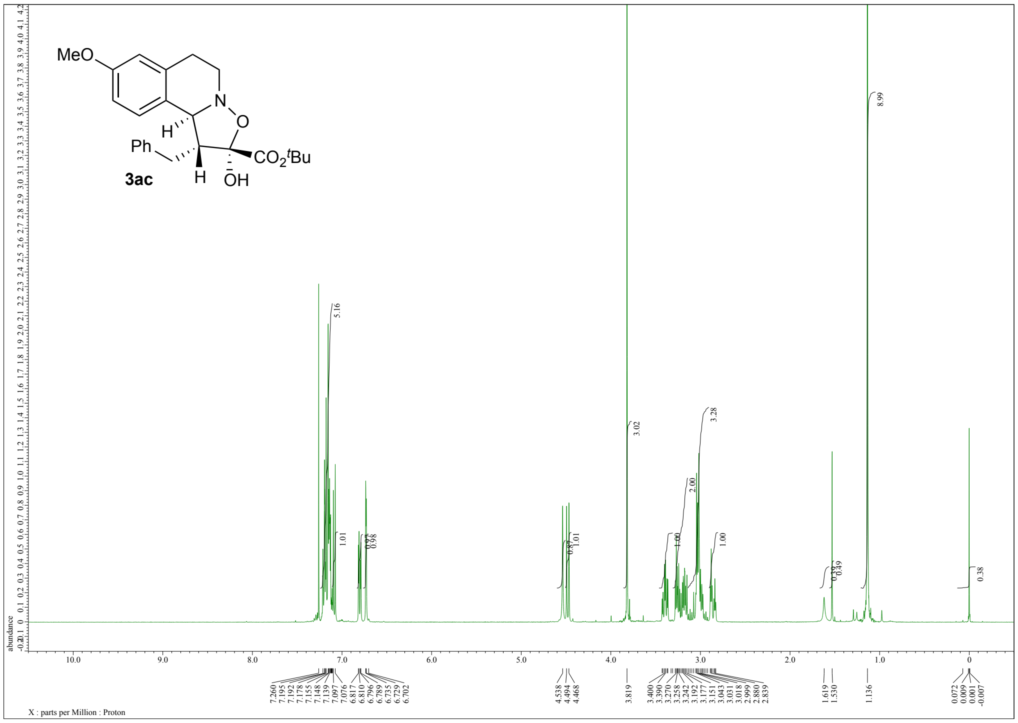

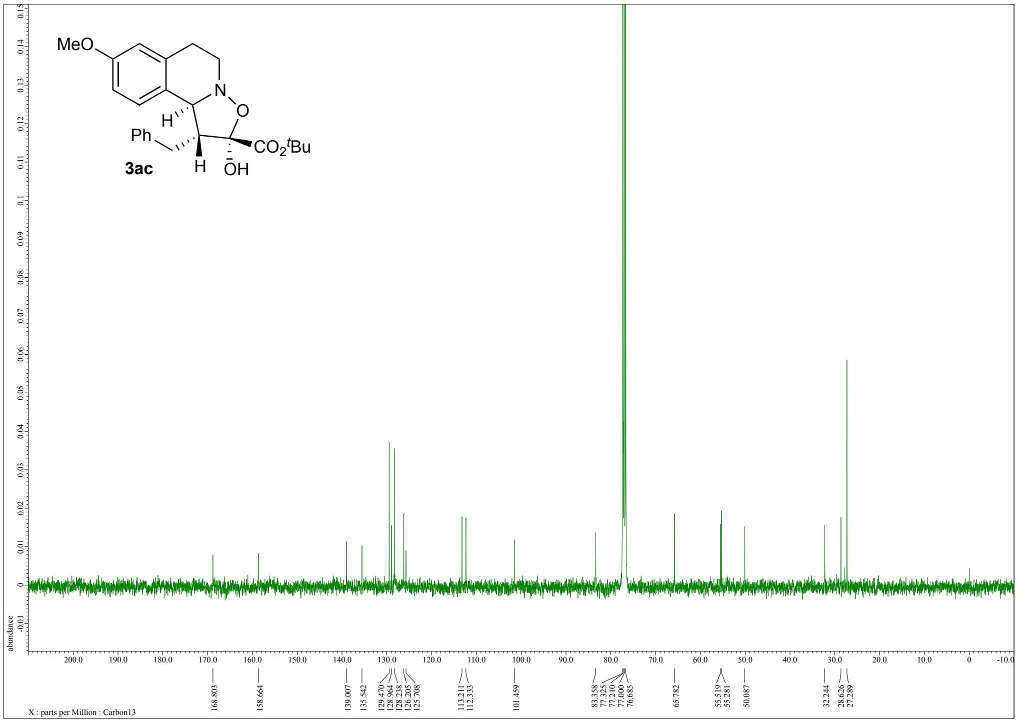

Supplementary Figure 34 | ^{1}H and ^{13}C NMR spectra of 3ac.

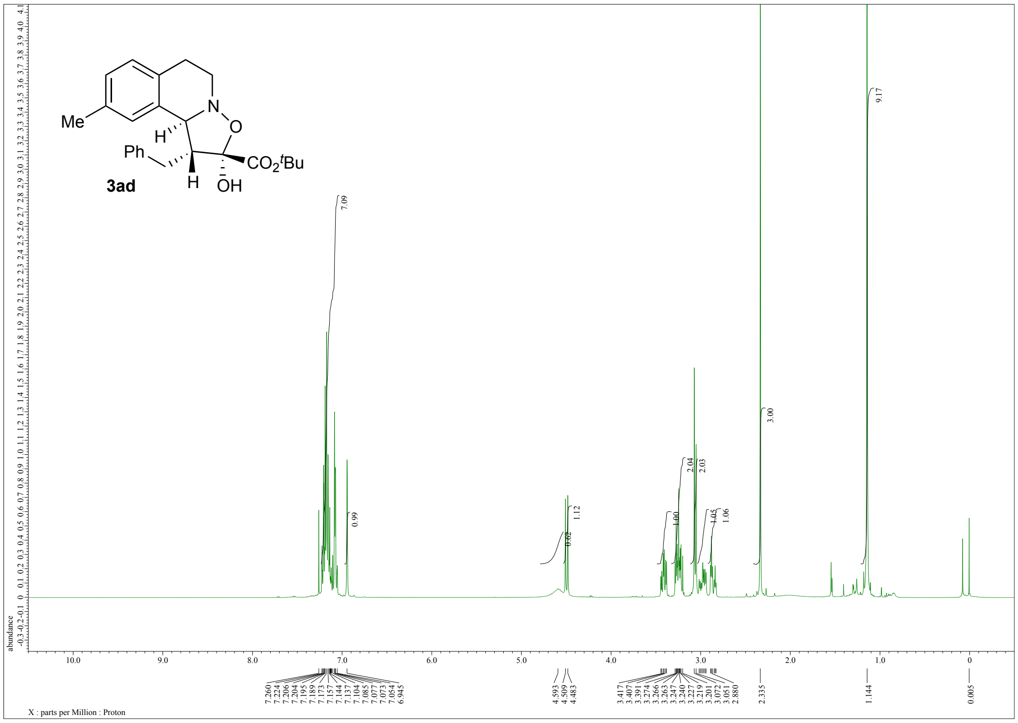

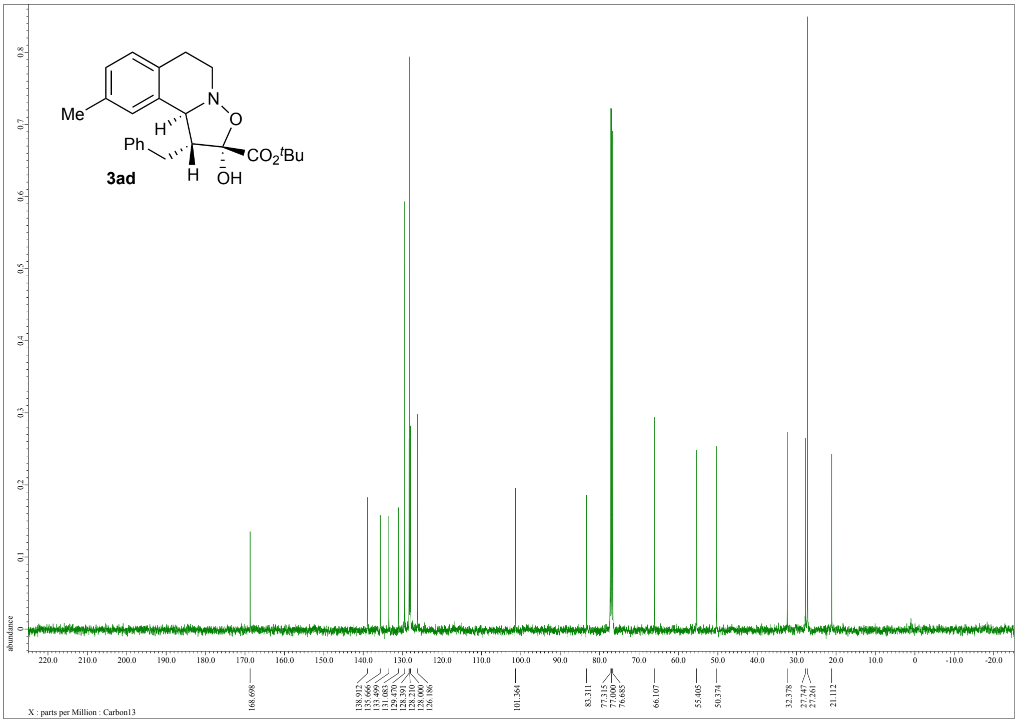

Supplementary Figure 35 | ^{1}H and ^{13}C NMR spectra of 3ad.

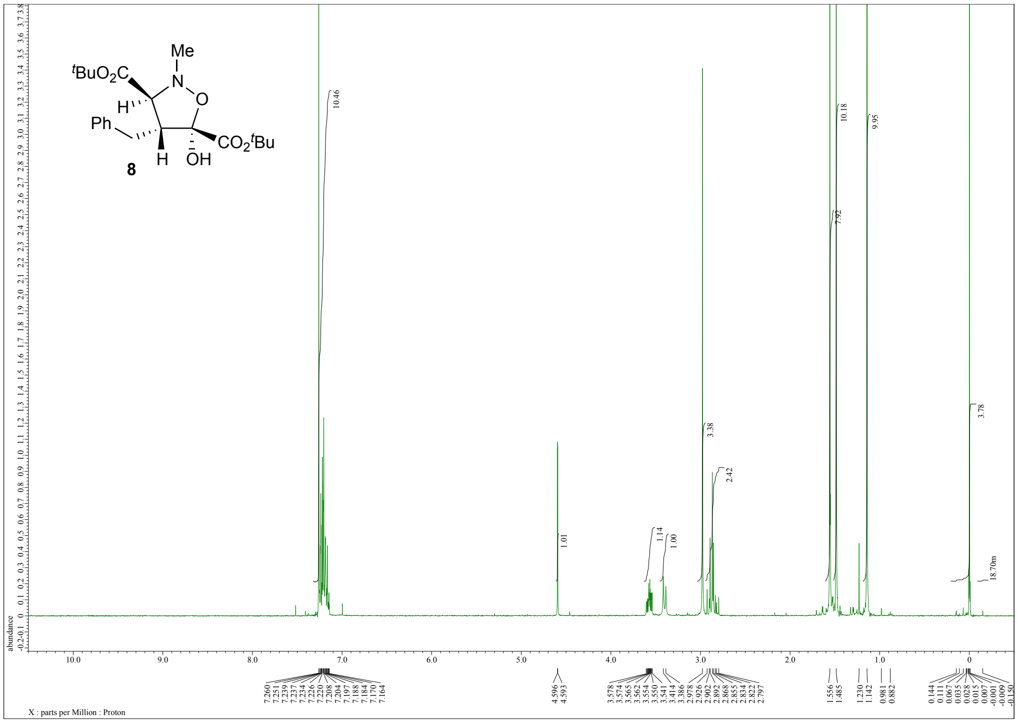

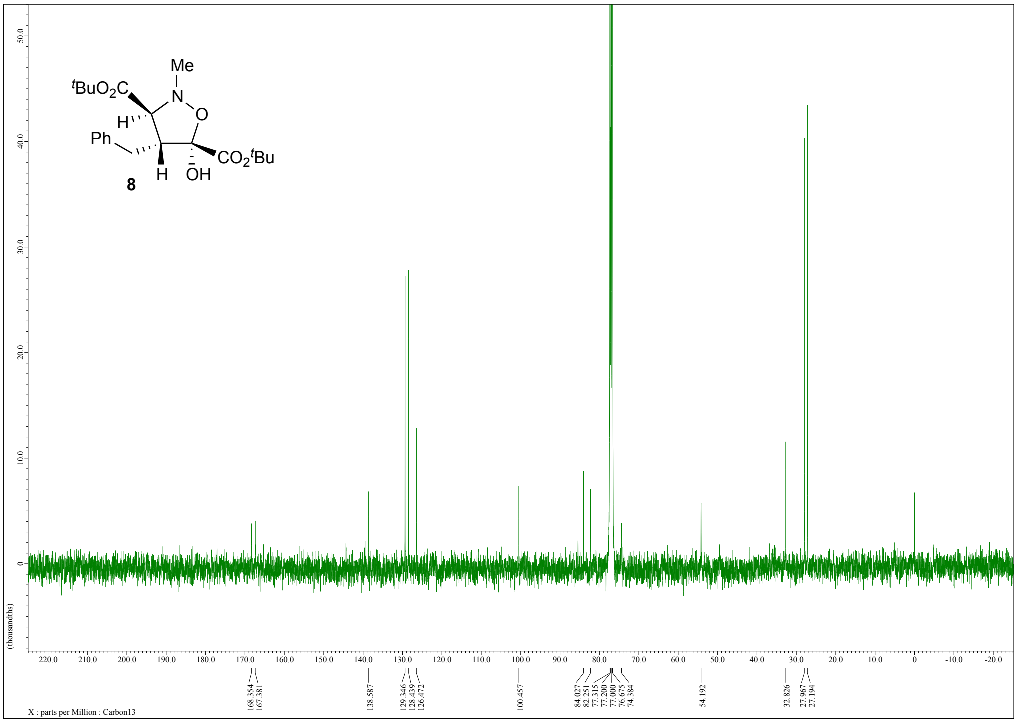

Supplementary Figure 36 | ^{1}H and ^{13}C NMR spectra of 8.

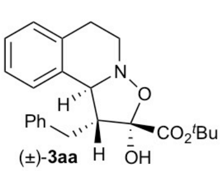

(±)-**3aa**

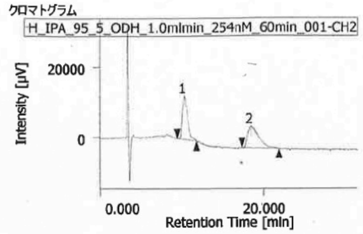

ピーク情報

#	ピーク名	CH	tR [min]	面積 [μV·sec]	高さ [μV]	面積%	高さ%
1	Unknown	2	10.276	564338	12323	49.844	67.298
2	Unknown	2	18.467	567865	5988	50.156	32.702

#	定量値	NTP	分離度	シンメトリー係数	警告
1	N/A	1155	4.375	1.809	
2	N/A	842	N/A	2.063	

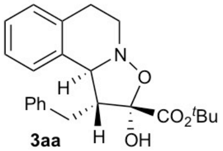

3aa
91% ee

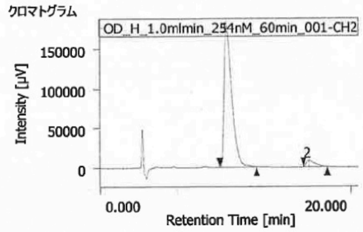

ピーク情報 (Area: 面積%)

#	ピーク名	CH	tR [min]	面積 [μV·sec]	高さ [μV]	面積%	高さ%
1	Unknown	2	10.042	7813810	17762	95.655	95.956
2	Unknown	2	16.667	354950	748	4.345	4.044

#	定量値	NTP	分離度	シンメトリー係数	警告
1	N/A	1248	5.575	1.912	
2	N/A	2862	N/A	1.859	

Supplementary Figure 37 | HPLC charts of 3aa.

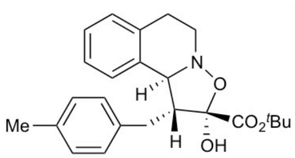

(±)-**3ba**

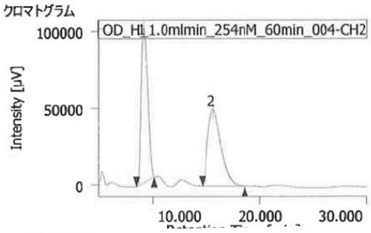

ピーク情報

#	ピーク名	CH	tR [min]	面積 [μV·sec]	高さ [μV]	面積%	高さ%
1	Unknown	2	9.167	4141240	100699	49.336	66.858
2	Unknown	2	15.625	4252717	49917	50.664	33.142

#	定量値	NTP	分離度	シンメトリー係数	警告
1	N/A	1039	3.773	1.402	
2	N/A	742	N/A	1.654	

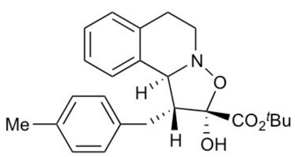

3ba
95% ee

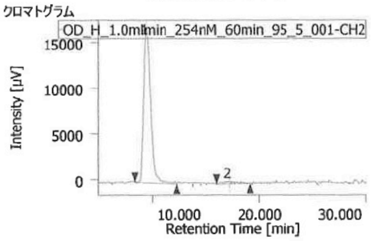

ピーク情報 (Area: 面積%)

#	ピーク名	CH	tR [min]	面積 [μV·sec]	高さ [μV]	面積%	高さ%
1	Unknown	2	9.467	751345	16861	97.357	98.623
2	Unknown	2	17.192	20401	235	2.643	1.377

#	定量値	NTP	分離度	シンメトリー係数	警告
1	N/A	1076	3.203	1.343	
2	N/A	349	N/A	1.322	

Supplementary Figure 38 | HPLC charts of 3ba.

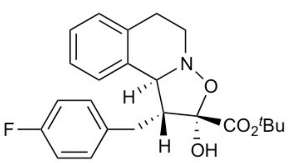

(±)-**3ca**

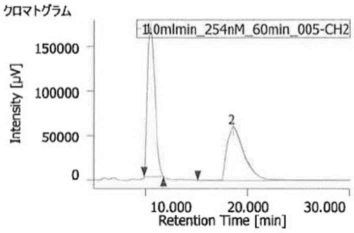

ピーク情報

#	ピーク名	CH	tR [min]	面積 [μV·sec]	高さ [μV]	面積%	高さ%
1	Unknown	2	10.450	8700179	167183	54.627	73.803
2	Unknown	2	18.608	7226261	59343	45.373	26.197

#	定量値	NTP	分離度	シンメトリー係数	警告
1	N/A	856	3.585	1.530	
2	N/A	564	N/A	1.783	

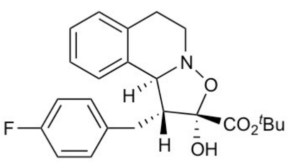

3ca
96% ee

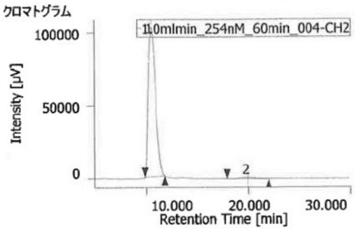

ピーク情報 (Area: 面積%)

#	ピーク名	CH	tR [min]	面積 [μV·sec]	高さ [μV]	面積%	高さ%
1	Unknown	2	10.492	5175871	10234	98.247	99.075
2	Unknown	2	19.942	92339	95	1.753	0.925

#	定量値	NTP	分離度	シンメトリー係数	警告
1	N/A	926	4.972	1.467	
2	N/A	1076	N/A	0.970	

Supplementary Figure 39 | HPLC charts of 3ca.

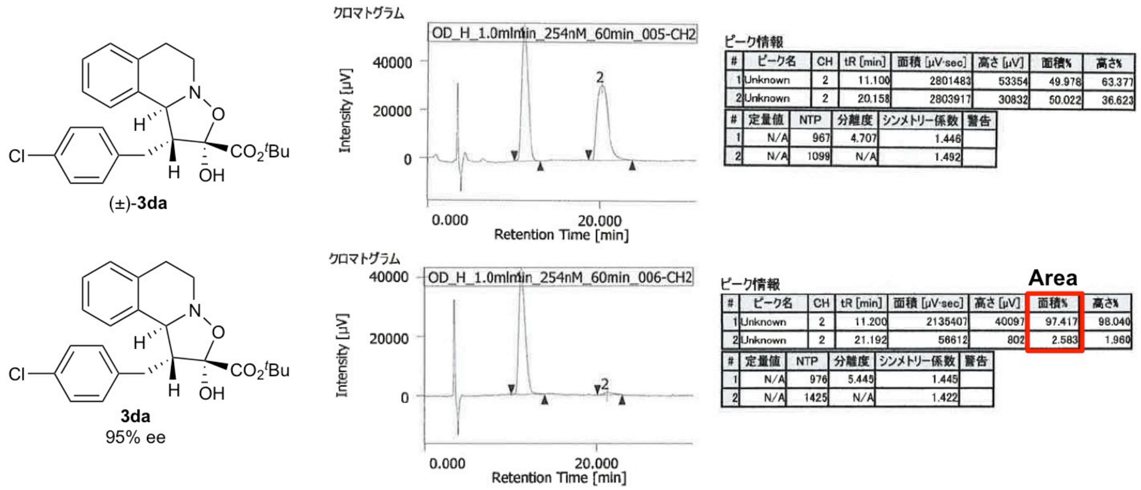

Supplementary Figure 40 | HPLC charts of 3da.

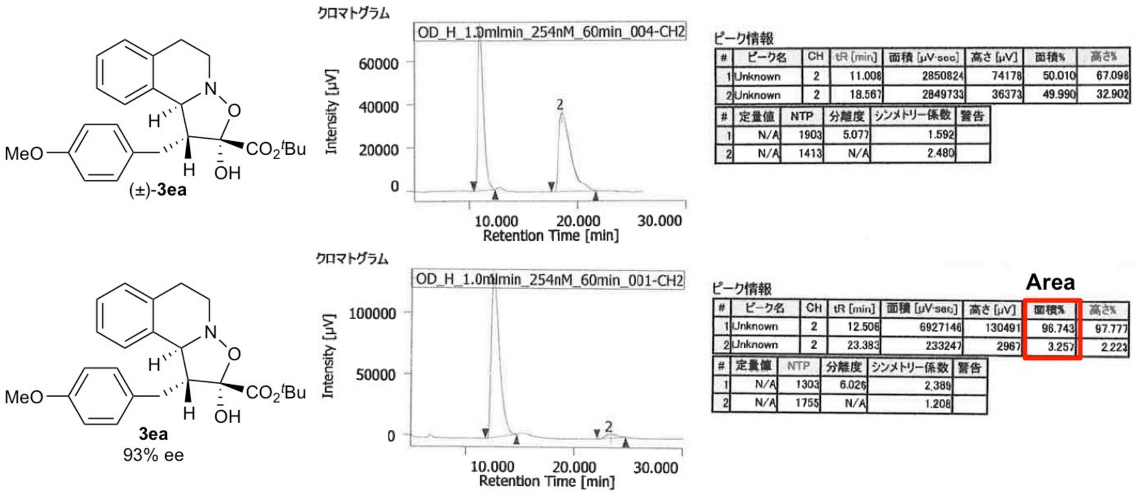

Supplementary Figure 41 | HPLC charts of 3ea.

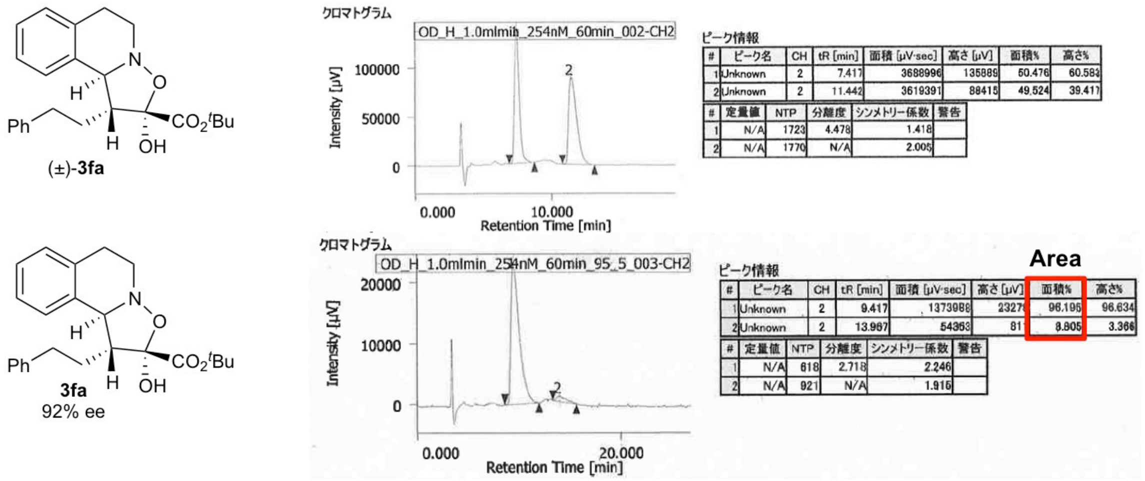

Supplementary Figure 42 | HPLC charts of 3fa.

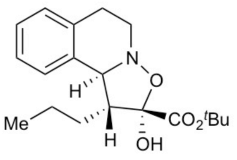

(±)-**3ga**

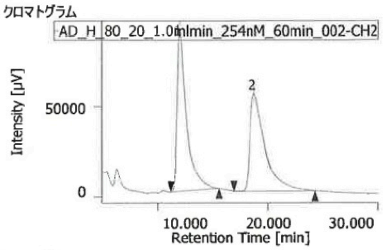

ピーク情報

#	ピーク名	CH	tR [min]	面積 [µV·sec]	高さ [µV]	面積%	高さ%
1	Unknown	2	12.033	5165145	88906	50.492	62.394
2	Unknown	2	18.733	5064495	53585	49.508	37.606

#	定量値	NTP	分離度	シンメトリー係数	警告
1	N/A	1321	3.778	2.476	
2	N/A	1127	N/A	2.636	

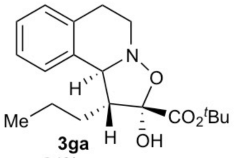

3ga
94% ee

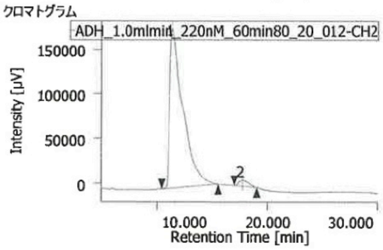

ピーク情報 **Area**

#	ピーク名	CH	tR [min]	面積 [µV·sec]	高さ [µV]	面積%	高さ%
1	Unknown	2	11.400	14797179	176706	97.391	96.390
2	Unknown	2	17.892	396430	6618	2.609	3.610

#	定量値	NTP	分離度	シンメトリー係数	警告
1	N/A	482	3.348	3.307	
2	N/A	1750	N/A	1.536	

Supplementary Figure 43 | HPLC charts of 3ga.

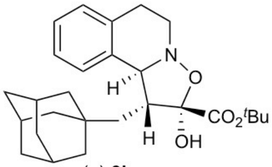

(±)-**3ha**

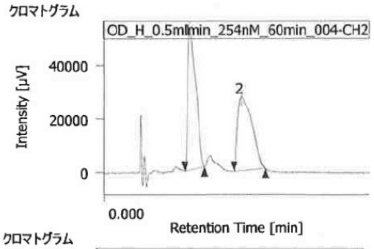

ピーク情報

#	ピーク名	CH	tR [min]	面積 [µV·sec]	高さ [µV]	面積%	高さ%
1	Unknown	2	15.683	5260195	52626	51.391	65.690
2	Unknown	2	25.025	4975534	27487	48.609	34.310

#	定量値	NTP	分離度	シンメトリー係数	警告
1	N/A	460	2.299	2.115	
2	N/A	367	N/A	2.259	

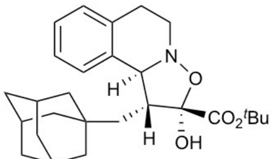

3ha
94% ee

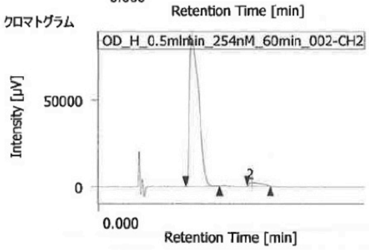

ピーク情報 **Area**

#	ピーク名	CH	tR [min]	面積 [µV·sec]	高さ [µV]	面積%	高さ%
1	Unknown	2	15.692	9073005	8497	96.813	97.503
2	Unknown	2	25.792	298629	217	3.187	2.497

#	定量値	NTP	分離度	シンメトリー係数	警告
1	N/A	419	2.770	2.178	
2	N/A	591	N/A	2.321	

Supplementary Figure 44 | HPLC charts of 3ha.

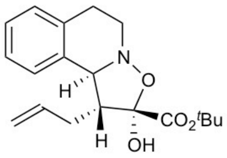

(±)-**3ia**

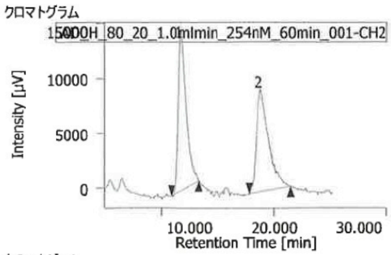

ピーク情報

#	ピーク名	CH	tR [min]	面積 [µV·sec]	高さ [µV]	面積%	高さ%
1	Unknown	2	11.742	694429	14548	49.892	61.279
2	Unknown	2	18.775	697444	9193	50.108	38.721

#	定量値	NTP	分離度	シンメトリー係数	警告
1	N/A	1526	4.518	1.950	
2	N/A	1531	N/A	1.902	

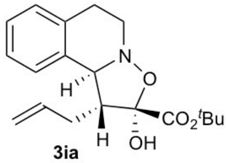

3ia
88% ee

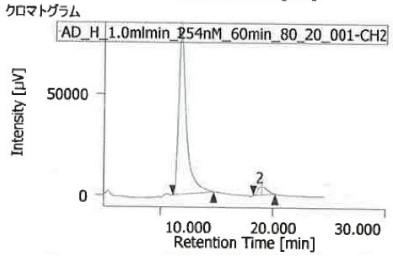

ピーク情報 **Area**

#	ピーク名	CH	tR [min]	面積 [µV·sec]	高さ [µV]	面積%	高さ%
1	Unknown	2	11.783	3805499	79990	94.096	94.877
2	Unknown	2	18.992	238771	4319	5.904	5.123

#	定量値	NTP	分離度	シンメトリー係数	警告
1	N/A	1875	5.556	2.307	
2	N/A	2519	N/A	1.359	

Supplementary Figure 45 | HPLC charts of 3ia.

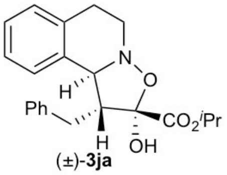

(±)-**3ja**

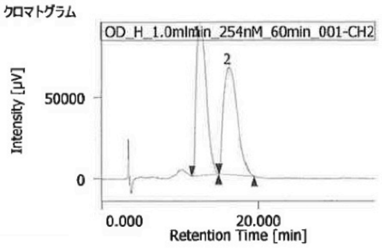

ピーク情報

#	ピーク名	CH	tR [min]	面積 [µV·sec]	高さ [µV]	面積%	高さ%
1	Unknown	2	12.456	8103648	88967	50.159	57.615
2	Unknown	2	16.283	8052271	65449	49.841	42.385

#	定量値	NTP	分離度	シンメトリー係数	警告
1	N/A	393	1.297	2.032	
2	N/A	367	N/A	1.549	

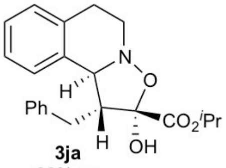

3ja
88% ee

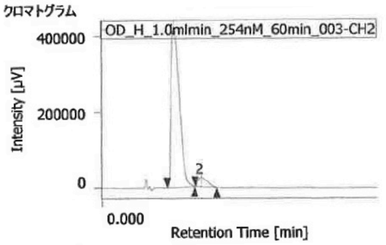

ピーク情報 (Area: 面積%)

#	ピーク名	CH	tR [min]	面積 [µV·sec]	高さ [µV]	面積%	高さ%
1	Unknown	2	11.200	37826738	419806	93.783	94.364
2	Unknown	2	15.442	2507545	25073	6.217	5.636

#	定量値	NTP	分離度	シンメトリー係数	警告
1	N/A	327	1.591	1.822	
2	N/A	464	N/A	1.675	

Supplementary Figure 46 | HPLC charts of 3ja.

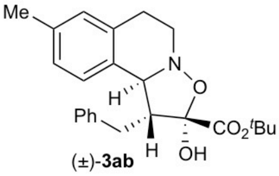

(±)-**3ab**

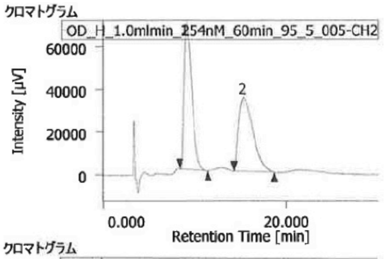

ピーク情報

#	ピーク名	CH	tR [min]	面積 [µV·sec]	高さ [µV]	面積%	高さ%
1	Unknown	2	9.142	3826293	65839	50.366	65.536
2	Unknown	2	15.350	3770627	34624	49.634	34.464

#	定量値	NTP	分離度	シンメトリー係数	警告
1	N/A	541	2.744	1.845	
2	N/A	429	N/A	1.826	

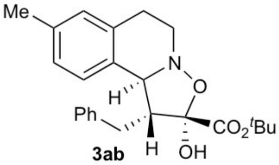

3ab
95% ee

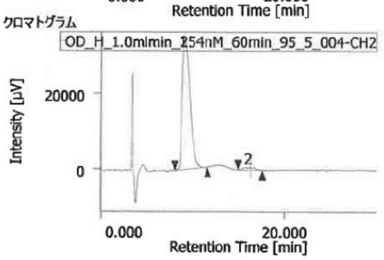

ピーク情報 (Area: 面積%)

#	ピーク名	CH	tR [min]	面積 [µV·sec]	高さ [µV]	面積%	高さ%
1	Unknown	2	9.125	1992007	34174	97.437	97.836
2	Unknown	2	16.242	52401	756	2.563	2.164

#	定量値	NTP	分離度	シンメトリー係数	警告
1	N/A	543	3.773	1.666	
2	N/A	859	N/A	1.028	

Supplementary Figure 47 | HPLC charts of 3ab.

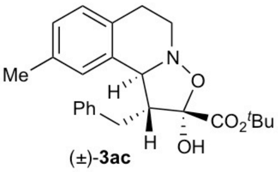

(±)-**3ac**

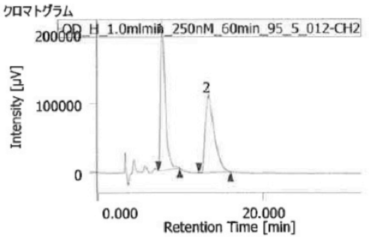

ピーク情報

#	ピーク名	CH	tR [min]	面積 [µV·sec]	高さ [µV]	面積%	高さ%
1	Unknown	2	7.817	8520492	206046	50.440	65.054
2	Unknown	2	13.392	8371960	110684	49.560	34.946

#	定量値	NTP	分離度	シンメトリー係数	警告
1	N/A	886	3.683	1.893	
2	N/A	728	N/A	1.888	

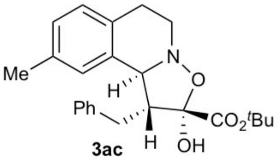

3ac
90% ee

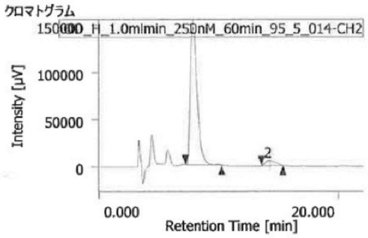

ピーク情報 (Area: 面積%)

#	ピーク名	CH	tR [min]	面積 [µV·sec]	高さ [µV]	面積%	高さ%
1	Unknown	2	7.833	5820202	14573	95.064	96.642
2	Unknown	2	14.258	302204	5064	4.936	3.358

#	定量値	NTP	分離度	シンメトリー係数	警告
1	N/A	891	4.640	1.633	
2	N/A	1090	N/A	1.260	

Supplementary Figure 48 | HPLC charts of 3ac.

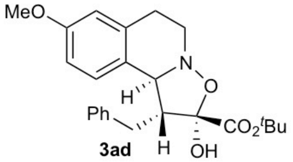

3ad

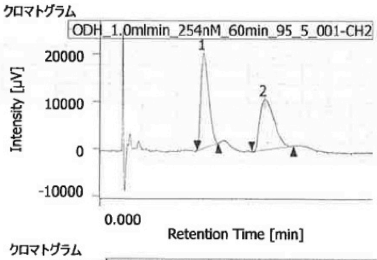

ピーク情報

#	ピーク名	CH	tR [min]	面積 [µV·sec]	高さ [µV]	面積%	高さ%
1	Unknown	2	15.492	1677123	20137	51.030	65.246
2	Unknown	2	24.758	1609452	10726	48.970	34.754

#	定量値	NTP	分離度	シンメトリー係数	警告
1	N/A	730	2.869	1.677	
2	N/A	560	N/A	1.728	

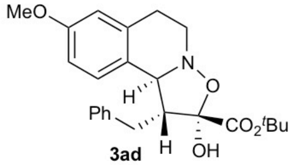

3ad
89% ee

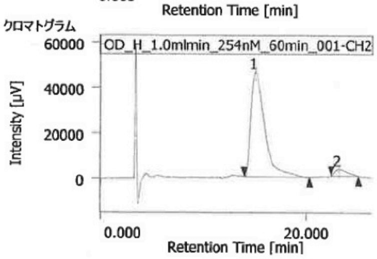

ピーク情報

Area

#	ピーク名	CH	tR [min]	面積 [µV·sec]	高さ [µV]	面積%	高さ%
1	Unknown	2	15.042	4259403	46199	94.531	93.927
2	Unknown	2	23.167	246420	2987	5.469	6.073

#	定量値	NTP	分離度	シンメトリー係数	警告
1	N/A	682	3.545	2.267	
2	N/A	1633	N/A	1.732	

Supplementary Figure 49 | HPLC charts of 3ad.

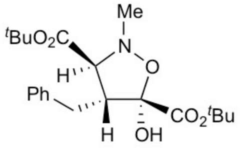

(±)-**8**

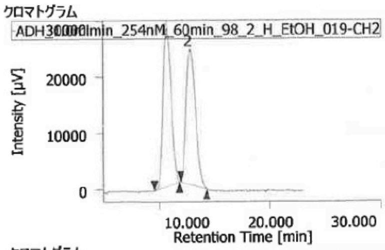

ピーク情報

#	ピーク名	CH	tR [min]	面積 [µV·sec]	高さ [µV]	面積%	高さ%
1	Unknown	2	10.692	1433951	28148	50.873	53.925
2	Unknown	2	12.825	1384712	24050	49.127	46.075

#	定量値	NTP	分離度	シンメトリー係数	警告
1	N/A	961	1.456	1.167	
2	N/A	1082	N/A	1.222	

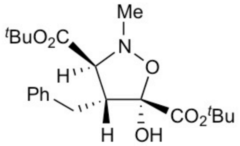

8
60% ee

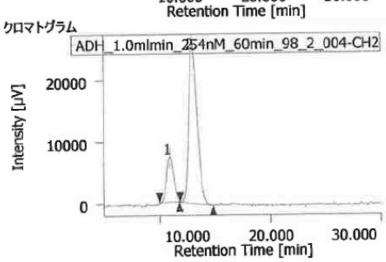

ピーク情報

Area

#	ピーク名	CH	tR [min]	面積 [µV·sec]	高さ [µV]	面積%	高さ%
1	Unknown	2	10.925	324267	7268	19.157	21.895
2	Unknown	2	13.008	1368379	25926	80.843	78.105

#	定量値	NTP	分離度	シンメトリー係数	警告
1	N/A	1313	1.605	1.092	
2	N/A	1388	N/A	1.180	

Supplementary Figure 50 | HPLC charts of 8.

Supplementary Tables

Supplementary Table 1 | Crystallographic data for mononuclear complex I (CCDC 1482739) and trinuclear complex II (CCDC 1482740).

	Mononuclear **I**	Trinuclear **II**
Molecular formula	$C_{22}H_{40}N_2NiO_5$, C_4H_8O	$C_{48}H_{86}N_4Ni_3O_{12}$, $2(H_2O)$
Formula weight	527.37	1123.37
T (K)	90	90
Wavelength (Å)	0.71073	0.71073
Color	Green	Pale green
Crystal system	Orthorhombic	Orthorhombic
Space group	$P2_12_12_1$	$C222_1$
a (Å)	11.1623(2)	12.5530(2)
b (Å)	13.7407(2)	19.8321(3)
c (Å)	17.7987(3)	22.2191(3)
V (Å^3)	2729.93(8)	5531.50(14)
Z	4	4
Density (Mg/m^3)	1.283	1.349
Absorption coefficient (mm^{-1})	0.747	1.074
F (000)	1144	2408
Crystal size (mm^3)	0.190 x 0.130 x 0.100	0.250 x 0.220 x 0.090
Theta range for data collection	1.872 to 30.986	0.92 to 30.09
(°)	75788	43987
Reflections collected	8684	8110
Independent reflections	SHELXL-2013	SHELXL-97
Software for refinements	1.056	1.080
Goodness of fit on F^2	0.0260, 0.0612	0.0300, 0.0819
R_1, wR_2 [$I>2\sigma(I)$]	0.0291, 0.0625	0.0327, 0.0836
R_1, wR_2 (all data)		

Supplementary Table 2 | Crystal data of 3aa (CCDC 1482737).

Molecular formula	$C_{23}H_{27}NO_4$
Formula weight	381.46
T (K)	170
Wavelength (Å)	1.54187
Color	Colorless
Crystal system	Orthorhombic
Space group	$P2_12_12_1$
a (Å)	5.71946(10)
b (Å)	13.0704(2)
c (Å)	26.3640(5)
β (°)	–
V (Å^3)	1970.86(6)
Z	4
Density (Mg/m^3)	1.286
Absorption coefficient (mm^{-1})	0.705
F(000)	816
Crystal size (mm^3)	0.192 x 0.087 x 0.073
Theta range for data collection (°)	3.35 to 68.24
Reflections collected	35331
Independent reflections	3596
Software for refinements	SHELXL-97
Goodness of fit on F^2	1.232
R_1, wR_2 [$I>2\sigma(I)$]	0.0314, 0.0663
R_1, wR_2 (all data)	0.0373, 0.0736

Supplementary Table 3 | Crystallographic data of 8 (CCDC 1482738).

Molecular formula	$C_{21}H_{31}NO_6$
Formula weight	381.46
T (K)	180
Wavelength (Å)	1.54184
Color	Colorless
Crystal system	Orthorhombic
Space group	$P2_12_12_1$
a (Å)	10.72181(19)
b (Å)	12.7059(2)
c (Å)	16.2362(3)
V (Å^3)	2211.87(7)
Z	4
Density (Mg/m^3)	1.182
Absorption coefficient (mm^{-1})	0.706
F (000)	848
Crystal size (mm^3)	0.338 x 0.320 x 0.302
Theta range for data collection (°)	3.480 to 68.185
Reflections collected	38455
Independent reflections	4036
Software for refinements	SHELXL-2013
Goodness of fit on F^2	1.079
R_1, wR_2 [$I>2\sigma(I)$]	0.0295, 0.0720
R_1, wR_2 (all data)	0.0306, 0.0729

Supplementary Table 4 | Calculated ECD spectrum of DFT-I.

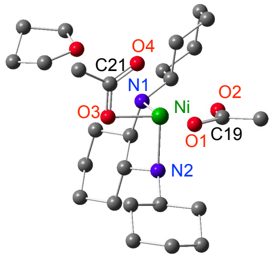

DFT-I

Ni–N1: 2.091 Å
Ni–N2: 2.109 Å
Ni–O1: 2.140 Å
Ni–O2: 2.101 Å
Ni–O3: 2.045 Å
Ni–O4: 2.302 Å

O(1)–C(19)–O(2): 120.44 °
O(3)–C(21)–O(4): 121.13 °

Excitation energies		<S**2>	Transition	W (%)
(eV)	(nm)			
0.8019	1546	2.006	MO β–128 –> MO β–138	12
			MO β–134 –> MO β–138	14
			MO β–136 –> MO β–138	26
0.9457	1311	2.006	MO β–132 –> MO β–138	14
			MO β–133 –> MO β–138	17
			MO β–135 –> MO β–138	10
			MO β–137 –> MO β–140	18
0.9786	1267	2.006	MO β–127 –> MO β–140	10
			MO β–133 –> MO β–138	11
			MO β–137 –> MO β–140	38
1.4514	854	2.006	MO β–127 –> MO β–138	15
			MO β–137 –> MO β–138	60
1.6958	731	2.006	MO β–132 –> MO β–140	13
			MO β–134 –> MO β–140	11
			MO β–136 –> MO β–140	26
1.7839	695	2.006	MO β–132 –> MO β–140	17
			MO β–133 –> MO β–140	25
			MO β–135 –> MO β–140	19

Transitions with a weight of less than 10% are not listed.

Supplementary Table 5 | Calculated ECD spectrum of DFT-II.

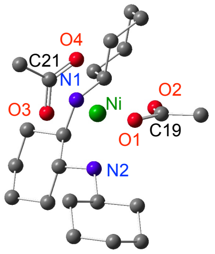

DFT-II

Ni–N1: 2.091 Å
Ni–N2: 2.109 Å
Ni–O1: 2.140 Å
Ni–O2: 2.101 Å
Ni–O3: 2.045 Å
Ni–O4: 2.302 Å

O(1)–C(19)–O(2): 120.44 °
O(3)–C(21)–O(4): 121.11 °

Excitation energies		<S**2>	Transition	W (%)
(eV)	(nm)			
0.9854	1258	2.006	MO β–113 –> MO β–118	27
			MO β–114 –> MO β–118	26
1.0421	1190	2.006	MO β–108 –> MO β–120	11
			MO β–116 –> MO β–120	11
			MO β–117 –> MO β–118	11
			MO β–117 –> MO β–120	33
1.0541	1176	2.007	MO β–109 –> MO β–118	11
			MO β–115 –> MO β–118	36
			MO β–116 –> MO β–119	13
1.5329	748	2.006	MO β–108 –> MO β–118	13
			MO β–116 –> MO β–118	11
			MO β–117 –> MO β–118	49
1.7732	699	2.006	MO β–109 –> MO β–120	10
			MO β–115 –> MO β–118	10
			MO β–115 –> MO β–120	22
			MO β–116 –> MO β–120	16
1.7253	719	2.006	MO β–113 –> MO β–120	20
			MO β–114 –> MO β–120	27
			MO β–115 –> MO β–120	16

Transitions with a weight of less than 10% are not listed.

The dominant contributions in the calculated ECD spectra both for **DFT-I** (Supplementary Figs 11, 12 and Supplementary Table 4) and **DFT-II** (Supplementary Figs 11, 12 and Supplementary Table 4) were found to be associated with d$\rightarrow$d transitions of the Ni(II).

Supplementary Note 1

For full author information for ref 55 in the main text, see ref 32 in Supplementary References.

Supplementary Methods

^{1}H and ^{13}C NMR spectra were recorded at room temperature on a JEOL JNM-ECS-400 NMR spectrometer at 400 and 100 MHz, respectively. The proton chemical shift values are reported in parts per million (ppm) downfield from tetramethylsilane and referenced to the proton resonance of $CHCl_3$ (δ 7.26). The carbon chemical shift values are reported in parts per million (ppm) downfield from tetramethylsilane and referenced to the carbon resonance of $CDCl_3$ (δ 77.0). Chemical shifts are reported in ppm and *J* values in Hz. The data are presented in the following order: chemical shift, signal area integration in natural numbers, multiplicity (s = singlet, d = doublet, t = triplet, q = quartet, quint = quintet, m = multiplet and/or multiple resonances, and br = broad) and coupling constant. ESI-MS spectra were measured on a Bruker micrOTOF-QII-RSL. Optical rotations were measured on a JASCO P-2200 digital polarimeter using 5 cm glass cells with a sodium 589 nm filter. Chiral HPLC analysis was performed on a JASCO HPLC system with the following components: pump, PU-2080 plus; detector, CD-2095 plus. Electronic absorption spectra were recorded with a JASCO V-670 spectrophotometer. CD spectra were recorded in the UV-Vis-NIR region (250-700 nm) with a JASCO J-820 spectropolarimeter, and in the NIR region (700-2000 nm) with a JASCO J-730 spectropolarimeter.

All reactions were performed under an argon atmosphere in flame-dried glassware with magnetic stirring. Anhydrous methanol (MeOH), dichloromethane (CH_2Cl_2) and tetrahydrofuran (THF) were purchased from Kanto. Other solvents used were purchased from Wako Pure Chemical Industries, Ltd. (Wako), and Tokyo Chemical Industry Co. Ltd. (TCI) and were used as received. Reactions conducted below room temperature were cooled using a PSL-1400 (Tokyo Rikakikai Co., Ltd.) or a PSL-1810 (EYELA). Analytical thin-layer chromatography (TLC) was performed on Silica gel 60 F254-coated glass plates (Merck) or 0.25 mm NH-coated silica gel (Fuji Silysia Chemical, Ltd. CHROMATOREX® NH) or Aluminium oxide 60 F_{254}; visualization of the developed chromatogram was performed by exposure to ultraviolet light (254 nm) and/or staining with cerium molybdate stain (Hanessian's stain). Flash column chromatography was performed using silica gel 60N (40-50 μm, Kanto Chemical Co., Inc.) and CHROMATOREX® NH (NH–DM1020, 100–200 mesh, Fuji Silysia Chemical, Ltd.).

Single crystal X-ray diffraction data were collected with a Rigaku RAXIS-RAPID imaging-plate diffractometer (Cu*K*α radiation) and a Rigaku AFC-8 diffractometer equipped with a Saturn70 CCD detector (Mo*K*α radiation).

Preparation of α-ketoesters 1

The known α-ketoesters **1** were synthesized according to the previously reported procedures (Fig. S1)[15]. α-Ketoesters **1h** and **1i** were newly synthesized using similar procedures, which were not optimized.

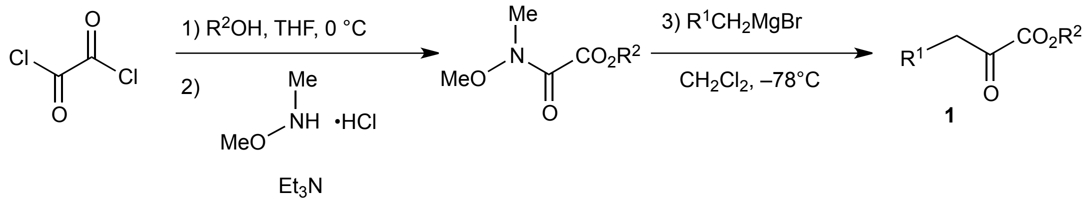

Typical procedure for preparation of S1

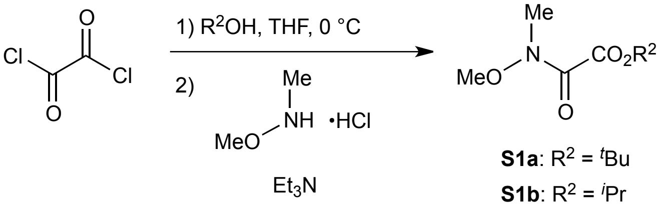

S1a: R^2 = ^{t}Bu
S1b: R^2 = ^{i}Pr

To a solution of oxalyl chloride (distilled, 22 mL, 257 mmol) in THF (400 mL) was added ^{t}BuOH (24 mL, 251 mmol) at 0 °C under an N_2 atmosphere. The mixture was stirred for 1 h at 0 °C, then *N*,*O*-dimethylhydroxylamine hydrochloride (25.0 g, 256 mmol) and triethylamine (107 mL, 769 mmol) were added, and the solution was further stirred for 2 h. The reaction was quenched with saturated NH_4Claq, and THF in the resulting mixture was evaporated under reduced pressure. The aqueous layer was extracted with ethyl acetate (100 mL x 3). The combined organic layers were washed with water (100 mL) and brine (100 mL), dried over Na_2SO_4, and concentrated under reduced pressure. The residue was purified by column chromatography (SiO_2, *n*-hexane/ethyl acetate = 5/1) to give **S1a** in 71% yield as pale yellow oil (33.7 g).

***tert*-Butyloxalic acid-*N*-methoxy-*N*-methylamide**[15]

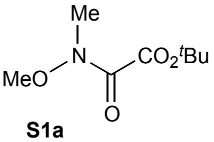

S1a

S1a: 66% yield; Pale yellow oil; IR (neat) 2982, 2941, 1736, 1674, 1260, 1151, 1090, 993, 844 cm^{-1}; ^{1}H NMR (400 MHz, $CDCl_3$) δ 3.75 (s, 3H), 3.20 (s, 3H), 1.56 (s, 9H); ^{13}C NMR (100 MHz, $CDCl_3$) δ 162.2, 161.7, 84.3, 62.1, 31.3, 28.0; HRMS-CI [M+H]$^+$ calcd for $C_8H_{16}NO_4$ 190.1079, found 190.1066.

***iso*-Propyloxalic acid-*N*-methoxy-*N*-methylamide**

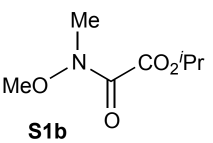

S1b

S1b: 85% yield; Pale yellow oil; IR (neat) 2980, 2943, 1737, 1674, 1459, 1390, 1344, 1251, 1178, 1148, 1085, 994, 932, 900, 840, 821, 763, 727, 655 cm^{-1}; ^{1}H NMR (400 MHz, $CDCl_3$) δ 5.21 (m, 1H), 3.75

(s, 3H), 3.22 (s, 3H), 1.35 (d, J = 6.4 Hz, 6H); ^{13}C NMR (400 MHz, $CDCl_3$) δ 162.1, 162.0, 70.2, 62.1, 31.2, 21.5; HRMS-ESI $[M+Na]^+$ calcd for $C_7H_{13}NNaO_4$ 198.0737, found 198.0713.

General procedure for preparation of α-ketoesters 1

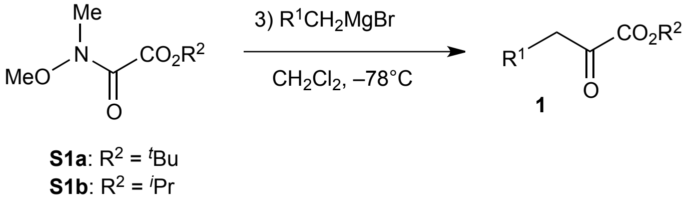

To a solution of magnesium turnings (972 mg, 40.0 mmol) in diethyl ether (5 mL) were added pieces of iodine. A solution of alkyl bromide (30.0 mmol) in diethyl ether (5 mL) was added dropwise over 30 min at 0 °C. The reaction mixture was stirred for 1 h to give the Grignard solution. The Grignard solution was added dropwise to a solution of **S1** (20.0 mmol) in dichloromethane (75 mL) over 30 min at –78 °C. The mixture was stirred at –78 °C for 1.5 h and then the reaction was quenched with saturated aqueous NH_4Claq (75 mL). The aqueous layer was extracted with dichloromethane (50 mL x 3). The combined organic layer was washed with brine (100 mL), dried over Na_2SO_4, and concentrated under reduced pressure. The residue was purified by column chromatography, and distilled or recrystallized to give the α-ketoester **1**.

***tert*-Butyl 4-(adamantan-1-yl)-2-oxo-butanoate**

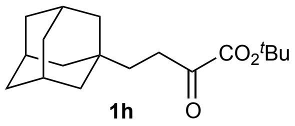

1h: 35% yield; Colorless oil; IR (neat) 2980, 2900, 2846, 1719, 1450, 1314, 1275, 1254, 1159, 1074, 1023, 839 cm^{-1}; ^{1}H NMR (400 MHz, $CDCl_3$) δ 2.74-2.70 (m, 2H), 1.96 (bs, 3H), 1.61-1.72 (m, 6H), 1.55 (s, 9H), 1.46-1.43 (br, 6H), 1.40-1.36 (m, 2H); ^{13}C NMR (100 MHz, $CDCl_3$) δ 196.5, 160.9, 83.8, 42.1, 37.0, 33.0, 31.8, 28.5, 27.8; HRMS-ESI $[M+Na]^+$ calcd for $C_{18}H_{28}NaO_3$ 315.1931, found 315.1924.

Isopropyl 2-oxo-4-phenylbutanoate

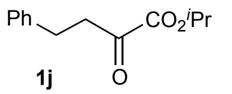

1j: 67% yield; Colorless oil; IR (neat) 2983, 1720, 1604, 1497, 1454, 1375, 1251, 1182, 1146, 1105, 1064, 1016, 911, 837, 748, 698 cm^{-1}; ^{1}H NMR (400 MHz, $CDCl_3$) δ 7.31-7.27 (m, 2H), 7.22-7.19 (m, 3H), 5.13 (septet, J = 6.4 Hz, 1H), 3.16 (t, J = 7.5 Hz, 2H), 2.95 (t, J = 7.5 Hz, 2H) 1.33 (d, J = 6.4 Hz, 6H); ^{13}C NMR (100 MHz, $CDCl_3$) δ 193.9, 160.5, 140.1, 128.5, 128.3, 126.3, 70.6, 40.9, 29.0, 21.5; HRMS-ESI $[M+Na]^+$ calcd for $C_{13}H_{16}NaO_3$ 243.0992, found 243.1004.

Preparation of nitrones[16]

The procedure was not optimized.

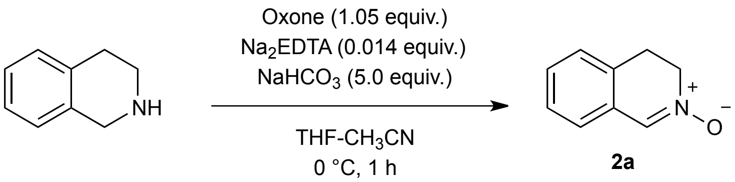

To a stirred solution of 1,2,3,4-tetrahydroisoquinoline (6.66 g, 50.0 mmol) in a mixture of acetonitrile/THF = 4/1 (87.5 mL) and 0.01 M aqueous EDTA solution (70 mL, 0.700 mmol) at 0 °C was added $NaHCO_3$ (21.0 g, 250 mmol). Under cooling to maintain the temperature at 0 °C, Oxone® monopersulfate compound (32.3 g, 52.5 mmol) was slowly added over 30 min under vigorous stirring. The mixture was stirred for 30 min at 0 °C and then ethyl acetate was added. The organic layer was separated and the aqueous layer was extracted with ethyl acetate. The combined organic extracts were dried over anhydrous $MgSO_4$ and concentrated under reduced pressure. The residue was purified by column chromatography (SiO_2, ethyl acetate then $CHCl_3$/MeOH = 20/1), and recrystallized (Et_2O at –25 °C) to give the corresponding nitrone **2a** in 44% yield as a pale yellow solid (3.20 g). In order to obtain pure nitrone, the recrystallization is important.

3,4-Dihydroisoquinoline 2-oxide[16]

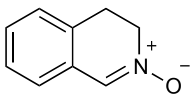

2a

2a: 44% yield; Pale yellow solid; Mp 56-57 °C; IR (neat) 3500, 2974, 2862, 1592, 1488, 1458, 1365, 1307, 1286, 1263, 1201, 1177, 1065, 906, 783, 761, 659 cm^{-1}; ^{1}H NMR (400 MHz, $CDCl_3$) δ 7.74 (s, 1H), 7.29-7.25 (m, 2H), 7.23-7.20 (m, 1H), 7.11-7.14 (m, 1H), 4.12 (t, J = 7.8 Hz, 2H), 3.19 (t, J = 7.8 Hz, 2H); ^{13}C NMR (100 MHz, $CDCl_3$) δ 134.0, 130.0, 129.4, 128.4, 127.6, 127.2, 125.4, 58.0, 27.8; HRMS-ESI $[M+Na]^+$ calcd for C_9H_9NNaO 170.0576, found 170.0562.

6-Methyl-3,4-dihydroisoquinoline 2-oxide[17]

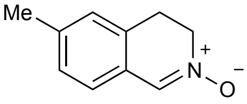

2b

2b: 12% yield; Pale yellow solid; Mp 86-88 °C; IR (neat) 3423, 3022, 2921, 1611, 1589, 1543, 1443, 1367, 1334, 1304, 1207, 1172, 1126, 1047, 948, 856, 817, 736, 619 cm^{-1}; ^{1}H NMR (400 MHz, $CDCl_3$) δ 7.72 (s, 1H), 7.08 (d, J = 7.8 Hz, 1H), 7.03-7.01 (m, 2H), 4.09 (t, J = 7.8 Hz, 2H), 3.14 (t, J = 7.8 Hz, 2H), 2.35 (s, 3H); ^{13}C NMR (100 MHz, $CDCl_3$) δ 139.8, 134.1, 130.0, 128.2, 128.1, 125.7, 125.3, 57.8, 27.8, 21.4; HRMS-ESI $[M+Na]^+$ calcd for $C_{10}H_{11}NaNO$ 184.0733, found 184.0746.

6-Methoxy-3,4-dihydroisoquinoline 2-oxide[18]

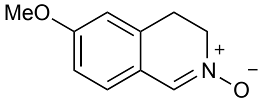

2d

2d: 28% yield; Pale yellow solid; Mp 53-54 °C; IR (neat) 3418, 1606, 1561, 1501, 1315, 1270, 1175, 1122, 1107, 1033, 853, 732, 637 cm^{-1}; ^{1}H NMR (400 MHz, $CDCl_3$) δ 7.71 (s, 1H), 7.07 (d, J = 8.3 Hz, 1H), 6.81-6.76 (m, 2H), 4.07 (t, J = 7.6 Hz, 9H), 3.83 (s, 3H), 3.15 (t, J = 7.6 Hz, 2H); ^{13}C NMR (100 MHz, $CDCl_3$) δ 160.6, 134.0, 132.1, 127.0, 121.3, 113.6, 112.6, 57.3, 55.5, 28.1; HRMS-ESI $[M+Na]^+$ calcd for $C_{10}H_{11}NaNO_2$ 200.0682, found 200.0693.

7-methyl-3,4-dihydroisoquinoline 2-oxide[17]

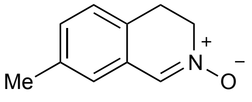

2e

2e: 7% yield; Pale yellow solid; Mp 98-101 °C; IR (neat) 3430, 1611, 1592, 1569, 1501, 1444, 1431, 1286, 1213, 1198, 898, 834, 810, 778, 668 cm^{-1}; ^{1}H NMR (400 MHz, $CDCl_3$) δ 7.71 (s, 1H), 7.10 (bs, 2H), 6.94 (s, 1H), 4.09 (t, J = 7.8 Hz, 2H), 3.14 (t, J = 7.8 Hz, 2H), 2.34 (s, 3H); ^{13}C NMR (100 MHz, $CDCl_3$) δ 137.4, 134.2, 130.1, 128.3, 127.1, 126.0, 58.2, 27.4, 20.9; HRMS-ESI $[M+Na]^+$ calcd for $C_{10}H_{11}NaNO$ 184.0733, found 184.0751.

The known 1,2,3,4-tetrahydroisoquinolines **S3b**[19], **S3d**[20] and **S3e**[19] were synthesized based on the Schmidt rearrangement[21]/reduction sequences starting from commercially available indanones (Fig. S2).

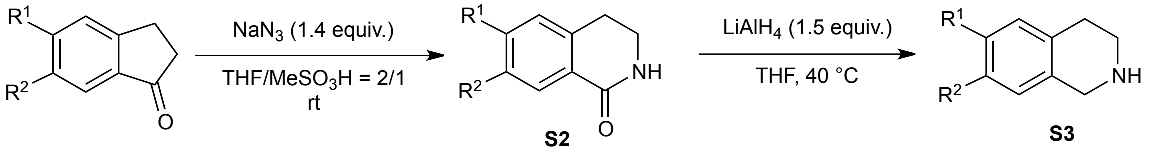

S2 **S3**

S2b: R^1 = Me, R^2 = H
S2d: R^1 = MeO, R^2 = H
S2e: R^1 = H, R^2 = Me

S3b: R^1 = Me, R^2 = H
S3d: R^1 = MeO, R^2 = H
S3e: R^1 = H, R^2 = Me

Preparation of Ni(II) complexes

Synthesis of (*R,R*)-4e[22,23]

(*R,R*)-**4e**[22] was synthesized based on the reported procedure[23].

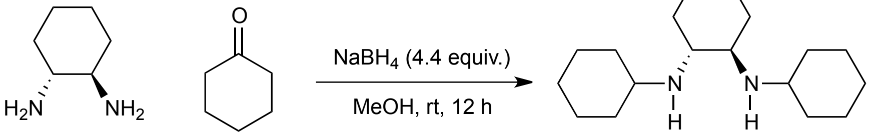

(4.0 equiv.) (*R,R*)-**4e**

To a solution of (*R,R*)-diaminocyclohexane (500.0 mg, 4.38 mmol) in MeOH (8.8 mL) was added cyclohexanone (1.81 mL, 17.5 mmol) at room temperature for 30 min. To the resulting mixture was added $NaBH_4$ (729.4 g, 19.3 mmol) at 0 °C. The reaction mixture was warmed to room temperature, stirred for 12 h, diluted with an excess of EtOAc, and then poured into water. The resulting mixture was partitioned between EtOAc and the aqueous layer. The aqueous layer was extracted with EtOAc (x 3) and the combined organic layers were dried over Na_2SO_4, and concentrated *in vacuo*. The residue was purified by column chromatography [SiO_2: *n*-hexane/EtOAc = 1/1, 0/1 then EtOAc/MeOH = 20/1, 5/1] to give (*R,R*)-**4e** (1.00 g, 82% yield).

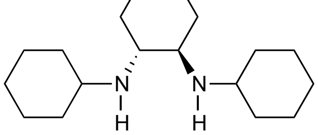

(*R,R*)-**4e**

(*R,R*)-**4e**: Pale yellow oil; IR (neat) 2924, 2852, 1448, 1115, 1113, 732 cm^{-1}; ^{1}H NMR (400 MHz, $CDCl_3$) δ 2.50-2.44 (m, 2H), 2.20-2.12 (m, 2H), 2.02 (dd, J = 10.6, 2.8 Hz, 2H), 1.87 (br d, J = 12.9 Hz, 2H), 1.77-1.63 (m, 10H), 1.58-1.52 (m,

2H), 1.33-1.07 (m, 10H), 1.02-0.91 (m, 4H); ^{13}C NMR (400 MHz, $CDCl_3$) δ 58.7, 53.4, 35.4, 33.2, 32.8, 26.2, 25.2, 24.8; HRMS-ESI $[M+H]^+$ calcd for $C_{18}H_{35}N_2$ 279.2795, found 279.2761; $[\alpha]_D^{23}$ –95.2 (*c* 1.31, $CHCl_3$).

(*R,R*)-**4e** was stocked as its HCl salt, because (*R,R*)-**4e** was sensitive to air. To a solution of the residue in MeOH was added 5~10% HCl/MeOH at 0 °C. A single recrystallization by vapor diffusion of EtOAc into MeOH solution at room temperature provided (*R,R*)-**4e**·HCl.

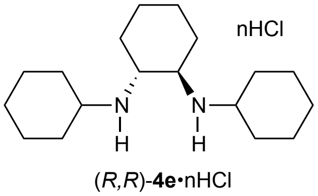

(*R,R*)-**4e**•nHCl

(*R,R*)-**4e**·nHCl: Colorless solid; Mp 250-255 °C; IR (neat) 2937, 2859, 2681, 2505, 2396, 2216, 1588, 1569, 1452, 1379, 1353, 1316, 1031, 919, 726, 669, 644 cm^{-1}; ^{1}H NMR (400 MHz, $CDCl_3$) δ 10.02 (bs, 2H), 9.63 (dd, *J* = 8.7 Hz, 2H), 3.94 (bs, 2H), 3.30 (dd, *J* = 4.1 Hz, 2H), 2.33 (d, *J* = 12.4 Hz, 2H), 2.19 (br t, *J* = 14.7 Hz, 4H), 2.04-1.64 (m, 16H), 1.45-1.18 (m, 8H); ^{13}C NMR (400 MHz, $CDCl_3$) δ 55.6, 55.0, 30.3, 28.0, 27.3, 24.8, 24.7, 24.6, 23.3; HRMS-ESI $[M–H–nCl]^+$ calcd for $C_{18}H_{35}N_2O_3$ 279.2795, found 279.2761; $[\alpha]_D^{25}$ –50.4 (*c* 1.00, $CHCl_3$).

Preparation of THF solution of the Ni(II)–complex

To a solution of (*R,R*)-**4e** (266.6 mg, 0.958 mol) in EtOH was added nickel acetate tetrahydrate (237.6 mg, 0.958 mmol) at room temperature. The mixture was stirred for 1.5 h at room temperature, and then filtered through a membrane filter (Chromatodisc 13N, KURABO). The filtrate was concentrated under reduced pressure to give the $Ni(OAc)_2·4H_2O$/(*R,R*)-**4e** = 1/1 complex as a green oil (428.8 mg, 0.813 mmol; calculated on the basis of the formula $Ni[(OAc)_2(H_2O)_4$(*R,R*)-**4e**]). Then, THF (4.07 mL) was added at room temperature to prepare the stock solution of $Ni(OAc)_2·4H_2O$/(*R,R*)-**4e** = 1/1 (0.2 M in THF). In Fig. 6 in the main text, this stock solution was used for the catalytic asymmetric formal [3+2] cycloadditions of α-ketoesters **1** and nitrones **2**.

Preparation of mononuclear Ni(II) complex I

After removal of THF (6.75 mL, 1.35 mmol; calculated on the basis of the formula $Ni[(OAc)_2(H_2O)_4$(*R,R*)-**4e**]) from the stock solution [$Ni(OAc)_2·4H_2O$/(*R,R*)-**4e** = 1/1 (0.2 M in THF)], the residue (692.5 mg) was dissolved in CH_2Cl_2 (~3 mL) at room temperature. Slow evaporation at –25 °C afforded rich green-colored crystals, which were collected by filtration to give mononuclear Ni(II)–diamine–acetates **I** in 86% yield (586.1 mg, 1.11 mmol; calculated on the basis of the formula $Ni[(OAc)_2$**4e**(THF)] (Supplementary Fig. 1)

Preparation of trinuclear Ni(II) complex II

After removal of THF (2.07 mL, 0.414 mmol; calculated on the basis of the formula $Ni[(OAc)_2(H_2O)_4$(*R,R*)-**4e**]) from the stock solution [$Ni(OAc)_2·4H_2O$/(*R,R*)-**4e** = 1/1 (0.2 M in THF)], the residue (300.1 mg) was dissolved in *n*-hexane (~5 mL) at room temperature. Slow evaporation at room temperature afforded rich green-colored crystals,

which were collected by filtration to give trinuclear Ni(II)–diamine–acetates **II** in 85% yield (Ni-based, 182.7 mg, 0.163 mmol; calculated on the basis of the formula $Ni_3[(OAc)_6$**4e**$_2(H_2O)_2]$) (Supplementary Fig. 2).

Catalytic formal [3+2] cycloaddition of 1a with 2a

MS 4A (100 mg, powder purchased from Nacalai Tesque, Inc.) in a Schlenk flask equipped with a magnetic stirring bar was flame-dried under reduced pressure for 5 min. Upon cooling to room temperature, the flask was refilled with N_2, and α-ketoester **1a** (23.4 mg, 0.10 mmol) and (*E*)-nitrone **2a** (17.7 mg, 0.12 mmol) were added and dried under vacuum. The flask was backfilled with N_2, THF (250 μL) was added at room temperature, and the flask was cooled to –30 °C. To the resulting solution was added the catalyst solution prepared (5 mol%: 25 μL, 0.2 M in THF) and $^iPr_2NH$ (10 mol%: 25 μL, 0.4 M in THF). The reaction mixture was stirred for 24 h at –30 °C. Aluminium oxide 60 (~20 mg, Merck) was added, and the mixture was diluted with EtOAc cooled at –30 °C. The solution was passed through a pad of Aluminium oxide 60, in order to remove the nickel catalyst, and then eluted with EtOAc and concentrated under reduced pressure. The diastereomeric ratio (>50/1) was determined from the ^{1}H NMR spectrum of the crude sample. The residue was purified by column chromatography [CHROMATOREX® NH (NH–DM1020, 100–200 mesh, Fuji Silysia Chemical, Ltd.) to give **3aa** in 74% yield (28.2 mg, 0.0740 mmol). The enantiomeric excess of (–)-(1*R*,2*R*,10b*S*)-**3aa** (91% e.e.) was determined by means of chiral HPLC analysis (CHIRALCEL OD-H, 0.46 cm (*ϕ*) × 25 cm (L), *n*-hexane/2-propanol = 95/5, 1.0 mL/min, major; 9.8 min, minor; 17.7 min).

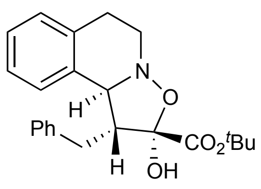

(1*R*,2*R*,10b*S*)-*anti-anti* **3aa**

(1*R*,2*R*,10b*S*)-*anti-anti* **3aa**: 71% yield (91% ee); Colorless solid; Mp 85-86 °C; IR (neat) 3479, 3027, 2977, 2932, 1603, 1455, 1394, 1369, 1233, 1215, 1143, 1077, 1063, 951, 910, 834, 734, 700, 673, 632, 605 cm^{-1}; ^{1}H NMR (400 MHz, $CDCl_3$) δ 7.25-7.11 (m, 9H), 4.55 (d, *J* = 10.1 Hz, 1H), 4.52 (bs, 1H), 3.43 (ddd, *J* = 11.1, 11.1, 4.1 Hz, 1H), 3.33-3.17 (m, 2H), 3.12-2.96 (m, 3H), 2.90 (ddd, *J* = 16.6, 4.1, 4.1 Hz, 1H), 1.13 (s, 9H); ^{13}C NMR (400 MHz, $CDCl_3$) δ 168.7, 139.0, 134.2, 133.7, 129.5, 128.4, 128.2, 127.9, 127.1, 126.2, 126.2, 101.3, 83.3, 66.1, 55.6, 50.2, 32.3, 28.1, 27.3; HRMS-ESI $[M+Na]^+$ calcd for $C_{23}H_{27}NNaO_4$ 404.1832, found 404.1832; $[\alpha]_D^{25}$ –58.6 (*c* 1.01, $CHCl_3$) (91% ee); HPLC (DAICEL CHIRALCEL OD-H, *n*-hexane/2-propanol = 95/5, 1.0 mL/min, 254 nm, t_{minor} 17.3 min, t_{major} 9.8 min).

The absolute stereochemistry of the major product *anti-anti* **3aa** was determined by X-ray analysis using optically pure **3aa** obtained by chiral HPLC separation using CHIRALCEL OD [2.0 cm (*ϕ*) × 25 cm (L)]. Thus, the absolute stereochemistry of the major product **3aa** was unequivocally elucidated as (1*R*,2*R*,10b*S*), in which the protons at C1, C10b and the hydroxyl group at C1 are pseudoaxial (Supplementary Fig. 15). Under optimal conditions, the 2-hydroxyisoxazolidinyl core in (1*R*,2*R*,10b*S*)-**3aa** is stable. No

epimerization occurred at the C2 position. Equilibrium of (1*R*,2*R*,10b*S*)-**3aa** with the corresponding hydroxyamine/α-ketoester was also not observed.

Spectral data for *anti-anti* 3 and *anti-anti*-8

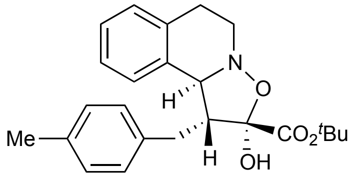

(1*R*,2*R*,10b*S*)-*anti-anti* **3ba**

(1*R*,2*R*,10b*S*)-**3ba**: 60% yield (95% e.e.); Colorless oil; IR (neat) δ 3494, 2976, 2931, 1728, 1514, 1493, 1475, 1456, 1393, 1369, 1456, 1291, 1232, 1216, 1143, 1078, 1058, 1036, 952, 835, 812, 756, 717, 669, 631 cm^{-1}; ^{1}H NMR (400 MHz, $CDCl_3$) δ 7.25-7.13 (m, 4H), 7.05-6.98 (m, 4H), 4.53 (bs, 1H), 3.42 (ddd, J = 11.0, 11.0, 4.1 Hz, 1H), 3.29-3.13 (m, 2H), 3.07-2.95 (m, 3H), 2.99 (ddd, J = 17.0, 4.1, 4.1 Hz, 1H), 2.25 (s, 3H), 1.14 (s, 9H); ^{13}C NMR (400 MHz, $CDCl_3$) 168.8, 135.8, 135.6, 134.3, 138.8, 129.3, 128.9, 128.4, 127.9, 127.1, 126.2, 101.3, 83.3, 66.1, 55.8, 50.2, 31.9, 28.1, 27.2, 20.9; HRMS-ESI $[M+Na]^+$ calcd for $C_{24}H_{29}NNaO_4$ 418.1989, found 418.1989; $[\alpha]_D^{25}$ –21.9 (*c* 0.79, $CHCl_3$) (95% e.e.); HPLC (DAICEL CHIRALCEL OD-H, *n*-hexane/2-propanol = 95/5, 1.0 mL/min, 254 nm, t_{minor} 15.6 min, t_{major} 9.2 min). The absolute stereochemistry was assigned by analogy.

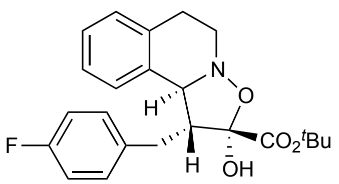

(1*R*,2*R*,10b*S*)-*anti-anti* **3ca**

(1*R*,2*R*,10b*S*)-**3ca**: 75% yield (96% e.e.); Colorless solid; Mp 108-111 °C; IR (neat) 3467, 2976, 2934, 2251, 1728, 1602, 1509, 1476, 1590, 1569, 1394, 1369, 1290, 1219, 1142, 1075, 1035, 1016, 951, 909, 835, 733, 672, 647, 630 cm^{-1}; ^{1}H NMR (400 MHz, $CDCl_3$) δ 7.26-7.10 (m, 6H), 6.91-6.87 (m, 2H), 4.59 (bs, 1H), 4.52 (d, J = 10.1 Hz, 1H), 3.41 (ddd, J = 10.6, 10.6, 4.1 Hz, 1H), 3.27 (ddd, J = 11.0, 4.6, 4.6 Hz, 1H), 3.18 (ddd, J = 10.6, 10.6, 4.6 Hz, 1H), 3.11 (m, 3H), 2.89 (ddd, J = 16.1, 4.1, 4.1 Hz, 1H), 1.16 (s, 9H); ^{13}C NMR (400 MHz, $CDCl_3$) δ 168.6, 161.5 (J_{CF} = 244.7 Hz), 134.6 (J_{CF} = 2.9 Hz), 134.3, 133.6, 130.9 (J_{CF} = 7.7 Hz), 128.4, 127.8, 127.2, 126.2, 114.9 (J_{CF} = 21.2 Hz), 101.3, 83.5, 66.1, 55.6, 50.3, 31.5, 28.1, 27.3; HRMS-ESI $[M+H]^+$ calcd for $C_{23}H_{26}FNNaO_4$ 422.1738, found 422.1751; $[\alpha]_D^{25}$ –12.6 (*c* 0.70, $CHCl_3$) (96% e.e.); HPLC (DAICEL CHIRALCEL OD-H, *n*-hexane/2-propanol = 95/5, 1.0 mL/min, 254 nm, t_{minor} 18.6 min, t_{major} 10.5 min). The absolute stereochemistry was assigned by analogy.

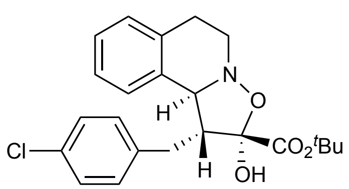

(1*R*,2*R*,10b*S*)-*anti-anti* **3da**

(1*R*,2*R*,10b*S*)-**3da**: 83% yield (95% e.e.); Colorless oil; IR (neat) 3474, 2976, 2935, 1728, 1491, 1456, 1410, 1394, 1369, 1233, 1215, 1142, 1291, 1076, 1035, 1016, 953, 909, 834, 807, 777, 760, 730, 660, 647, 29, 605 cm^{-1}; ^{1}H NMR (400 MHz, $CDCl_3$) δ 7.27-7.11 (m, 6H), 7.09-7.05 (m, 2H), 4.54 (bs, 1H), 4.51 (d, J = 10.6 Hz, 1H), 3.39 (ddd, J = 10.8, 10.8, 4.1 Hz, 1H), 3.27 (ddd, J = 11.0, 4.6, 4.6 Hz1H), 3.17 (ddd, J = 10.6, 10.6, 5.1 Hz, 1H), 3.09-2.97 (m, 3H), 2.89 (ddd, J = 16.6, 4.1, 4.1 Hz, 1H),

1.16 (s, 9H); ^{13}C NMR (400 MHz, $CDCl_3$) δ 168.6, 137.4, 134.3, 133.5, 132.1, 130.9, 128.5, 128.3, 127.8, 127.3, 126.2, 101.1, 83.6, 66.1, 55.4, 50.2, 31.7, 28.2, 27.3; HRMS-ESI $[M+Na]^+$ calcd for $C_{23}H_{26}ClNNaO_4$ 438.1443, found 438.1441; $[\alpha]_D^{25}$ –73.2 (*c* 0.95, $CHCl_3$) (95% e.e.); HPLC (DAICEL CHIRALCEL OD-H, *n*-hexane/2-propanol = 95/5, 1.0 mL/min, 254 nm, t_{minor} 20.2 min, t_{major} 11.1 min). The absolute stereochemistry was assigned by analogy.

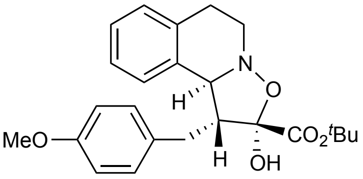

(1*R*,2*R*,10b*S*)-*anti-anti* **3ea**

(1*R*,2*R*,10b*S*)-**3ea**: 70% yield (93% e.e.); Colorless oil; IR (neat) 3490, 2977, 2933, 2835, 1611, 1584, 1511, 1492, 1456, 1297, 1176, 1142, 1076, 1035, 951, 835, 777, 759, 735, 701, 672, 630 cm^{-1}; ^{1}H NMR (400 MHz, $CDCl_3$) δ 7.29-7.12 (m, 4H), 7.06 (d, *J* = 8.73, 2H), 6.74 (d, *J* = 8.73, 2H), 4.54 (bs, 1H), 4.52 (d, *J* = 10.6 Hz, 1H), 3.73 (s, 3H), 3.42 (ddd, *J* = 10.6, 10.6, 4.1 Hz, 1H), 3.26 (ddd, *J* = 11.5, 4.6, 4.6 Hz, 1H), 3.18 (ddd, 15.6, 15.6, 6.0 Hz, 1H), 3.06-2.95 (m, 3H), 2.90 (ddd, *J* = 16.1, 4.1, 4.1 Hz, 1H), 1.16 (s, 9H); ^{13}C NMR (400 MHz, $CDCl_3$) δ 168.8, 158.1, 134.3, 133.8, 131.0, 130.4, 128.4, 127.9, 127.1, 126.2, 113.7, 101.4, 83.3, 66.1, 55.9, 55.2, 50.2, 31.4, 28.1, 27.3; HRMS-ESI $[M+Na]^+$ calcd for $C_{24}H_{29}NNaO_5$ 434.1938, found 434.1938; $[\alpha]_D^{25}$ –67.4 (*c* 1.38, $CHCl_3$) (93% e.e.); HPLC (DAICEL CHIRALCEL OD-H, *n*-hexane/2-propanol = 95/5, 1.0 mL/min, 254 nm, t_{minor} 18.6 min, t_{major} 11.0 min). The absolute stereochemistry was assigned by analogy.

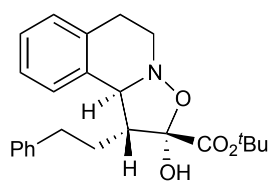

(1*R*,2*R*,10b*S*)-*anti-anti* **3fa**

(1*R*,2*R*,10b*S*)-**3fa**: 75% yield (92% e.e.); Colorless oil; IR (neat) 3446, 3065, 3025, 2977, 2934, 2865, 1731, 1603, 1496, 1477, 1456, 1494, 1370, 1295, 1276, 1258, 1215, 1159, 1085, 1030, 1000, 968, 911, 842, 803, 746, 701, 669, 644, 610 cm^{-1}; ^{1}H NMR (400 MHz, $CDCl_3$) δ 7.29-7.07 (m, 9H), 4.63 (bs, 1H), 4.43 (d, *J* = 11.0 Hz, 1H), 3.43 (ddd, *J* = 11.0, 11.0, 4.1 Hz, 1H), 3.31 (ddd, *J* = 11.0, 4.6, 4.6 Hz, 1H), 3.07-2.99 (m, 1H), 2.89 (ddd, *J* = 16.3, 4.1, 4.1 Hz, 1H), 2.63 (ddd, *J* = 12.6, 12.6, 4.8 Hz, 1H), 2.38 (ddd, *J* = 12.4, 12.4, 4.6 Hz, 1H), 2.19-2.06 (m, 1H), 2.00-1.92 (m, 1H), 1.48 (s, 9H); ^{13}C NMR (400 MHz, $CDCl_3$) δ 169.9, 141.9, 134.1, 133.6, 128.4, 128.3, 128.2, 127.9, 127.1, 126.1, 125.9, 101.7, 83.4, 66.5, 53.3, 50.3, 33.7, 28.9, 28.3, 27.6 ; HRMS-ESI $[M+Na]^+$ calcd for $C_{24}H_{29}NNaO_4$ 418.1989, found 418.1984; $[\alpha]_D^{26}$ +13.1 (*c* 1.31, $CHCl_3$) (92% e.e.); HPLC (DAICEL CHIRALCEL OD-H, *n*-hexane/2-propanol = 95/5, 1.0 mL/min, 254 nm, t_{minor} 15.0 min, t_{major} 9.8 min). The absolute stereochemistry was assigned by analogy.

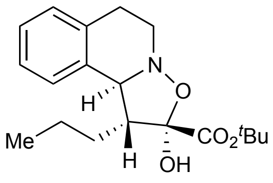

(1*R*,2*R*,10b*S*)-*anti-anti* **3ga**

(1*R*,2*R*,10b*S*)-**3ga**: 61% yield (94% e.e.); Colorless oil; IR (neat) 2963, 1739, 1457, 1393, 1370, 1297, 1248, 1157, 1071, 1053, 909, 849, 762, 742, 668, 607 cm^{-1}; ^{1}H NMR (400 MHz, $CDCl_3$) δ 7.24-7.19 (m, 2H), 7.18-7.13 (m, 2H), 4.45 (bs, 1H), 4.38 (d, *J* = 11.0 Hz, 1H), 3.41 (ddd, *J* = 11.0, 11.0, 4.1 Hz, 1H), 3.28 (ddd, *J* = 11.0, 4.6, 4.6 Hz, 1H), 3.02 (ddd, *J* = 16.1, 10.6, 5.1 Hz, 1H), 2.96-2.90 (m, 1H), 2.87 (ddd, *J* = 16.1, 4.1,

4.1 Hz), 1.86-1.72 (m, 1H), 1.48 (s, 9H), 1.41-1.22 (m, 1H), 1.12-1.02 (m,1H), 0.89 (t, *J* = 7.4 Hz, 3H); ^{13}C NMR (400 MHz, $CDCl_3$) δ 170.0, 134.2, 133.9, 128.3, 128.0, 127.0, 126.0, 101.7, 83.7, 66.5, 53.2, 50.3, 28.7, 28.4, 27.7, 20.7, 14.5; HRMS-ESI $[M+Na]^+$ calcd for $C_{19}H_{27}NNaO_4$ 356.1832, found 356.1835; $[\alpha]_D^{25}$ –9.1 (*c* 0.68, $CHCl_3$) (94% e.e.); HPLC (DAICEL CHIRALCEL OD-H, *n*-hexane/2-propanol = 95/5, 1.0 mL/min, 254 nm, t_{minor} 18.7 min, t_{major} 12.0 min). The absolute stereochemistry was assigned by analogy.

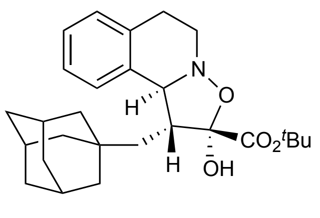

(1*R*,2*R*,10b*S*)-*anti-anti* **3ha**

(1*R*,2*R*,10b*S*)-**3ha**: 74% yield (94% e.e.); Colorless oil; IR (neat) 2904, 1731, 1453, 1370, 1291, 1254, 1221, 1162, 1103, 1073, 911, 835, 736, 669, 648 cm^{-1}; ^{1}H NMR (400 MHz, $CDCl_3$) δ 7.28-7.13 (m, 4H), 4.50 (br s, 1H), 4.28 (d, *J* = 10.6 Hz, 1H), 3.51 (ddd, *J* = 13.8, 8.3, 5.3 Hz, 1H), 3.22 (ddd, *J* = 11.5, 5.1, 5.1 Hz, 1H), 3.00-2.92 (m, 3H), 1.90 (dd, *J* = 15.2, 8.7 Hz, 1H,), 1.85 (br s, 3H), 1.64-1.50 (m, 6H), 1.49 (s, 9H), 1.34-1.22 (m, 6H); ^{13}C NMR (400 MHz, $CDCl_3$) δ 170.0, 134.3, 133.8, 128.2, 127.1, 125.8, 128.2, 128.1, 127.1, 125.8, 102.3, 83.8, 68.1, 50.3, 48.0, 42.4, 42.1, 39.7, 36.9, 32.1, 28.4, 28.1, 27.7; HRMS-ESI $[M+Na]^+$ calcd for $C_{27}H_{37}NNaO_4$ 462.2615, found 462.2607; $[\alpha]_D^{25}$ –22.7 (*c* 0.99, $CHCl_3$) (94% e.e.); HPLC (DAICEL CHIRALCEL OD-H, *n*-hexane/2-propanol = 95/5, 1.0 mL/min, 254 nm, t_{minor} 25.0 min, t_{major} 15.7 min). The absolute stereochemistry was assigned by analogy.

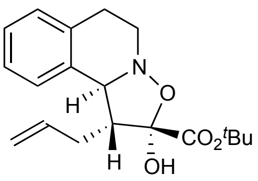

(1*R*,2*R*,10b*S*)-*anti-anti* **3ia**

(1*R*,2*R*,10b*S*)-**3ia**: 77% yield (88% e.e.); Colorless oil; IR (neat) 2978, 1736, 1642, 1493, 1456, 1394, 1369, 1293, 1246, 1153, 1082, 1058, 914, 836, 740 cm^{-1}; ^{1}H NMR (400 MHz, $CDCl_3$) δ 7.25-7.20 (m, 2H), 7.18-7.11 (m, 2H), 5.71-5.61 (m, 1H), 5.10 (ddd, *J* = 17.1, 3.2, 1.4 Hz, 1H), 4.98-4.93 (ddd, *J* = 10.1, 1.0, 1.0 Hz, 1H), 4.54 (bs, 1H), 4.43 (d, *J* = 10.6 Hz, 1H), 3.41 (ddd, *J* = 11.0, 11.0, 4.1 Hz, 1H), 3.28 (ddd, *J* = 11.0, 4.6, 4.6 Hz, 1H), 3.04-2.95 (m, 2H), 2.88 (ddd, *J* = 16.2, 4.1, 4.1 Hz, 1H), 2.59-2.51 (m, 1H), 2.47-2.40 (m, 1H), 1.44 (s, 9H); ^{13}C NMR (400 MHz, $CDCl_3$) δ 169.5, 135.0, 134.2, 133.7, 128.3, 127.8, 127.1, 126.1, 117.5, 101.5, 83.7, 65.9, 53.4, 50.2, 30.9, 28.1, 27.6; HRMS-ESI $[M+Na]^+$ calcd for $C_{19}H_{25}NNaO_4$ 354.1676, found 354.1687; $[\alpha]_D^{25}$ –11.7 (*c* 1.03, $CHCl_3$) (88% e.e.); HPLC (DAICEL CHIRALCEL OD-H, *n*-hexane/2-propanol = 95/5, 1.0 mL/min, 254 nm, t_{minor} 18.8 min, t_{major} 11.7 min). The absolute stereochemistry was assigned by analogy.

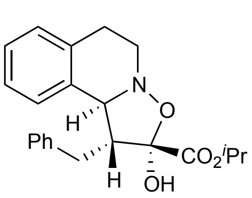

(1*R*,2*R*,10b*S*)-*anti-anti* **3ja**

(1*R*,2*R*,10b*S*)-**3ja**: 77% yield (88% e.e.); Colorless oil; IR (neat) δ 3466, 3065, 3030, 2979, 2935, 1737, 1603, 1495, 1455, 1282, 1230, 1191, 1147, 1107, 1077, 953, 911, 835, 741, 700, 669, 634, 604 cm^{-1}; ^{1}H NMR (400 MHz, $CDCl_3$) δ 7.31-7.09 (m, 9H), 4.64-4.60 (m, 1H), 4.56 (d, *J* = 10.1 Hz, 1H), 4.47 (br s, 1H), 3.44 (ddd, *J* = 10.1, 10.1, 4.1 Hz, 1H), 3.30-3.23 (m, 2H), 3.08-3.05 (m, 3H), 2.99 (dd, *J* = 10.1, 4.6 Hz, 1H), 2.91 (ddd, *J* = 16.4, 4.4, 4.4 Hz), 1.05 (d, *J* = 6.4 Hz, 3H), 0.85 (d, *J* = 6.0 Hz, 3H); ^{13}C NMR (400 MHz, $CDCl_3$) δ 169.2, 138.7, 134.3, 133.7, 129.4, 128.4, 128.1, 127.8, 127.2, 126.2, 126.2, 101.1, 70.7, 66.0, 55.9, 50.3,

32.3, 28.0, 21.2, 21.1; HRMS-ESI [M+Na]$^+$ calcd for $C_{22}H_{25}NNaO_4$ 390.1676, found 390.1689; $[\alpha]_D^{25}$ –50.5 (*c* 1.16, $CHCl_3$) (88% e.e.); HPLC (DAICEL CHIRALCEL OD-H, *n*-hexane/2-propanol = 95/5, 1.0 mL/min, 254 nm, t_{minor} 16.3 min, t_{major} 12.5 min). The absolute stereochemistry was assigned by analogy.

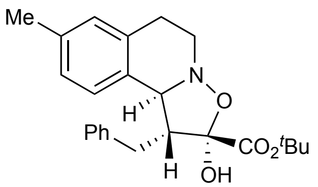

(1*R*,2*R*,10b*S*)-*anti-anti* **3ab**

(1*R*,2*R*,10b*S*)-**3ab**: 75% yield (95% e.e.); Colorless oil; IR (neat) 3479, 3016, 2977,1727, 1496, 1456, 1395, 1370, 1290, 1216, 1142, 1062, 949, 908, 835, 815, 699, 667, 623 cm^{-1}; ^{1}H NMR (400 MHz, $CDCl_3$) δ 7.20-7.12 (m, 5H), 7.08-7.01 (m, 3H), 4,59 (bs, 1H), 4.50 (d, *J* = 10.6 Hz, 1H), 3.39 (ddd, *J* = 10.6, 10.6, 3.7 Hz, 1H), 3.30-3.18 (m, 2H), 3.05 (d, *J* = 7.8 Hz, 2H), 2.99 (dd, *J* = 10.6, 5.1 Hz, 1H), 2.85 (ddd, *J* = 16.3, 4.1, 4.1 Hz, 1H), 2.35 (s, 3H), 1.13 (s, 9H); ^{13}C NMR (400 MHz, $CDCl_3$) δ 168.8, 139.0, 136.8, 134.0, 130.6, 129.5, 129.0, 128.2, 127.8, 127.0, 126.1, 101.4, 83.3, 65.9, 55.4, 50.2, 32.2, 28.3, 27.2, 21.0; HRMS-ESI [M+Na]$^+$ calcd for $C_{24}H_{29}NNaO_4$ 418.1989, found 418.1978; $[\alpha]_D^{26}$ –59.2 (*c* 0.91, $CHCl_3$) (95% e.e.); HPLC (DAICEL CHIRALCEL OD-H, *n*-hexane/2-propanol = 95/5, 1.0 mL/min, 254 nm, t_{minor} 15.4 min, t_{major} 9.1 min). The absolute stereochemistry was assigned by analogy.

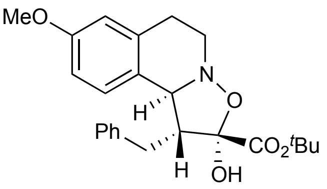

(1*R*,2*R*,10b*S*)-*anti-anti* **3ac**

(1*R*,2*R*,10b*S*)-**3ac**: 78% yield (89% e.e.); Colorless oil; IR (neat) 3452, 2977, 2931, 1730, 1612, 1504, 1456, 1394, 1369, 1279, 1253, 1231, 1162, 1077, 1039, 951, 910, 846, 752, 701, 632 cm^{-1}; ^{1}H NMR (400 MHz, $CDCl_3$) δ 7.22-7.12 (m, 5H), 7.09 (d, *J* = 8.5 Hz, 1H), 6.80 (d, *J* = 8.5, 2.8 Hz, 1H), 6.73 (d, *J* = 2.8 Hz, 1H), 4.54 (bs, 1H), 4.48 (d, *J* = 10.6 Hz, 1H), 3.82 (s, 3H), 3.59 (ddd, *J* = 11.0, 11.0, 4.1 Hz, 1H), 3.28-3.15 (m, 2H), 3.07-2.96 (m, 3H), 2.86 (ddd, *J* = 16.6, 4.1, 4.1 Hz, 1H), 1.14 (s, 9H); ^{13}C NMR (400 MHz, $CDCl_3$) δ 168.8, 158.7, 139.0, 135.5, 129.5, 129.0, 128.2, 126.2, 125.7, 113.2, 112.3, 101.5, 83.5, 65.8, 55.5, 55.3, 50.1, 32.2, 28.6, 27.3; HRMS-ESI [M+Na]$^+$ calcd for $C_{24}H_{29}NNaO_5$ 434.1938, found 434.1936; $[\alpha]_D^{25}$ –35.1 (*c* 1.01, $CHCl_3$) (89% e.e.); HPLC (DAICEL CHIRALCEL OD-H, *n*-hexane/2-propanol = 95/5, 1.0 mL/min, 254 nm, t_{minor} 24.8 min, t_{major} 15.5 min). The absolute stereochemistry was assigned by analogy.

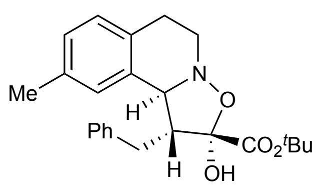

(1*R*,2*R*,10b*S*)-*anti-anti* **3ad**

(1*R*,2*R*,10b*S*)-**3ad**: 73% yield (90% e.e.); Colorless oil; IR (neat) 3482, 2976, 2931, 1728, 1603, 1496, 1475, 1455, 1394, 1369, 1291, 1252, 1231, 1143, 1078, 1062, 1031, 960, 835, 819, 801, 731, 699, 645, 613 cm^{-1}; ^{1}H NMR (400 MHz, $CDCl_3$) δ 7.21-7.05 (m, 7H), 6.95 (s, 1H), 4.59 (bs, 1H), 4.50 (d, *J* = 10.6 Hz, 1H), 3.39 (ddd, *J* = 3.70 Hz, 10.6 Hz, 1H), 3.29-3.16 (m, 2H), 3.09-2.95 (m, 3H), 2.90-2.81 (m, 1H), 2.35 (s, 3H), 1.13 (s, 9H); ^{13}C NMR (400 MHz, $CDCl_3$) δ 168.7, 138.9, 135.7, 133.5, 131.1, 129.5, 128.4, 128.2, 128.0, 126.2, 101.4, 83.3, 66.1, 55.4, 50.4, 32.4, 27.7, 27.3, 21.1; HRMS-ESI [M+Na]$^+$ calcd for $C_{24}H_{29}NNaO_4$ 418.1989, found 418.1973; $[\alpha]_D^{26}$ –39.9 (*c*

1.09, $CHCl_3$) (90% e.e.); HPLC (DAICEL CHIRALCEL OD-H, *n*-hexane/2-propanol = 95/5, 1.0 mL/min, 254 nm, t_{minor} 13.4 min, t_{major} 7.8 min).

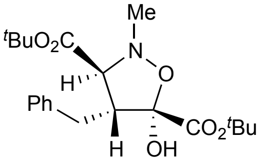

(3*S*,4*R*,5*R*)-*anti-anti*-**8**

(3*S*,4*R*,5*R*)-**8**: 90% yield (64% e.e.); Colorless oil; IR (neat) 2982, 1736, 1497, 1457, 1395, 1370, 1295, 1253, 1063, 1012, 910, 844, 735, 700, 667 cm^{-1}; ^1H NMR (400 MHz, $CDCl_3$) δ 7.26-7.14 (m, 5H), 4.60 (d, J = 1.4 Hz, 1H), 3.61-3.53 (m, 1H), 2.98 (s, 3H), 2.93-2.78 (m, 2H), 1.49 (s, 9H), 1.14 (s, 9H); ^{13}C NMR (400 MHz, $CDCl_3$) δ 168.4, 167.4, 138.6, 129.3, 128.4, 126.5, 100.5, 84.0, 82.3, 74.4, 54.2, 32.8, 28.0, 27.2; HRMS-ESI $[M+Na]^+$ calcd for $C_{21}H_{31}NNaO_6$ 416.2044, found 416.2044; $[\alpha]_D^{24}$ –4.49 (*c* 1.03, $CHCl_3$) (64% e.e.); HPLC (DAICEL CHIRALCEL AD-H, *n*-hexane/EtOH = 98/2, 1.0 mL/min, 254 nm, t_{minor} 10.7 min, t_{major} 12.8 min).

The absolute stereochemistry of *anti-anti*-**8** was also determined by X-ray analysis of optically pure *anti-anti*-**8** (Supplementary Fig. 16) obtained by chiral HPLC separation using CHIRALCEL AD [2.0 cm (ϕ) × 25 cm (L)].

Electron density distribution analysis of I·THF

A block-shaped, pale-green single crystal of **I·THF**, 0.19 × 0.13 × 0.10 mm, was selected for measurements. The diffraction data were collected using a RIGAKU AFC-8 diffractometer equipped with a Saturn70 CCD detector with Mo$K\alpha$ radiation by an oscillation method at 90 K. X-rays were monochromated and focused by a confocal mirror. Sixteen data sets were measured with different crystal orientations and detector positions; (i) $\chi = 40$, $\phi = 270$ and $2\theta = 40°$, (ii) $\chi = 50$, $\phi = 135$ and $2\theta = 40°$, (iii) $\chi = 50$, $\phi = 180$ and $2\theta = 40°$, (iv) $\chi = 50$, $\phi = 90$ and $2\theta = 40°$, (v) $\chi = 50$, $\phi = 45$ and $2\theta = 40°$, (vi) $\chi = 0$, $\phi = 0$ and $2\theta = 40°$, (vii) $\chi = 50$, $\phi = 0$ and $2\theta = 20°$, (viii) $\chi = 50$, $\phi = 90$ and $2\theta = 20°$, (ix) $\chi = 50$, $\phi = 180$ and $2\theta = 20°$, (x) $\chi = 50$, $\phi = 270$ and $2\theta = 20°$, (xi) $\chi = 0$, $\phi = 0$ and $2\theta = 20°$, (xii) $\chi = 50$, $\phi = 180$ and $2\theta = 83°$, (xiii) $\chi = 40$, $\phi = 270$ and $2\theta = 83°$, (xiv) $\chi = 50$, $\phi = 135$ and $2\theta = 83°$, (xv) $\chi = 50$, $\phi = 90$ and $2\theta = 83°$, and (xvi) $\chi = 0$, $\phi = 0$ and $2\theta = 83°$. Exposure time and oscillation angle for each frame were 2 sec and 0.3°, 5 sec and 0.5°, and 32 sec and 0.5° for the data sets with $2\theta = 20$, 40 and 83°, respectively. For all data sets, camera distance was 40 mm. Bragg spots were integrated, scaled and averaged up to $\sin\theta / \lambda = 1.22$ Å^{-1} by the program HKL2000[24] Lorentz and polarization corrections were applied during the scaling processes. Analytical absorption corrections[25] were applied. The numbers of measured and independent reflections, completeness, and R_{int} was 296729, 41303, 0.996 and 0.0401, respectively, up to $\sin\theta / \lambda = 1.22$ Å^{-1}.

The initial structure of **I·THF** was solved by a direct method using the programs SIR2004[26], and refined by a full matrix least-squares method on F^2 using the program SHELXL2014[27]. All hydrogen atoms were located on difference Fourier maps. All the hydrogen atoms were refined isotropically. High-order refinements were carried out in order to determine the position of non-hydrogen atoms against 36296 independent reflections with $0.60 \leq \sin\theta / \lambda \leq 1.22$ Å^{-1}. In the refinements, the positions of the hydrogen atoms bonding to carbon atoms were constrained and C−H and N−H distances of 1.099, 1.092, 1.059 and 1.099 Å were adopted for methyne, methylene, methyl and amino groups, respectively. Refinements with a multipole expansion method using the Hansen-Coppens multipole formalism[28] and topological analyses based on the resulted parameters were performed with the XD2006 package[29]. The refinements were carried out against 20771 independent reflections of $\sin\theta / \lambda \leq 1.22$ Å^{-1} with $I > 3\sigma(I)$ based on F^2. Electronic neutralization constraints were imposed for **I·THF** and heptane (crystal solvent) molecules. At the first stage of the refinements, the atomic coordinates and temperature factors of the atoms were fixed on those obtained from the high-order refinements. The population parameters, P_v, $P_{lm\pm}$ of the non-hydrogen atoms, and scales were refined. The levels of the multipoles were raised stepwise to hexadecapole and octupole for Ni, and O, N and C atoms, respectively, and dipole along the bonds for H atoms. Chemical equivalent constraints were applied for multipole parameters of all atoms. The radial screening parameters, κ and κ', were refined after the refinements of multipole parameters at hexadecapole level for non-hydrogen atoms. The refinement cycles were repeated twice. At the second stage, the temperature factors were refined by applying harmonic anisotropic models for non-H atoms and isotropic models for H atoms, following the refinements of the radial screening parameters, κ and κ'. The chemical

equivalent constraints on the multipole parameters were relaxed gradually, and finally all chemical constraints were removed. At the final stage, the coordinates (only structurally ordered parts), multipoles and temperature factors were refined. The C−H and N−H distances were constrained to be 1.099, 1.092, 1.059 and 1.099 Å for methyne, methylene, methyl and amino groups, respectively. The number of parameters in the final cycle of the refinements was 912.

Crystal data of **I** for electron density distribution analysis: $C_{22}H_{40}N_2NiO_5 \cdot C_4H_8O$, FW = 527.37, T = 90 K, orthorhombic, $P2_12_12_1$, a = 11.1679(1) Å, b = 13.7608(1) Å, c = 17.8036(2) Å, V = 2736.04(4) Å^3; D_X = 1.280 Mg m^{-3}; Z = 4; μ(Mo $K\alpha$) = 0.746 mm^{-1}, $R(F)$ = 0.0155, $wR(F^2)$ = 0.0305, and S = 0.9884 for 20771 reflections of $(\sin\theta / \lambda)_{max}$ = 1.22 Å^{-1} with $I > 3\sigma(I)$. $\Delta\rho_{min,\ max}$ = −0.234, 0.368 e Å^{-3}. CCDC deposition code: CCDC1482741.

On the basis of the final population parameters of multipoles, the numbers of occupied electrons in the 3d-orbital of the Ni atom were estimated as follows[30]: d_{xz}; 2.124(16), d_{yz}; 2.010(15), d_{xy}; 1.870(15), d_{z^2}; 1.598(16), and $d_{x^2-y^2}$; 1.355(15).

Computational details

All density functional theory (DFT) calculations were performed for the triplet configurations by means of unrestricted M06 functional (UM06)[31] using the Gaussian 09 program package[32]. Geometry optimizations, vibrational analyses and time-dependent density functional theory (TD-DFT) calculations[33] were carried out using the SDD basis set for nickel, and 6-311G(d,p) basis set for all other atoms (C, H, N and O). The solvent effect of THF was taken into account using the self-consistent reaction field (SCRF) method.

Cartesian coordinates (in Å) of DFT-I

SCF Done: E(UM06) = -1675.82260091 A.U.

Center number	Atomic number	Atomic type	Coordinates (Angstroms) X	Y	Z
1	28	0	0.727931	0.715631	0.562553
2	8	0	2.380227	1.781120	1.407312
3	8	0	1.194258	2.595324	-0.250677
4	8	0	0.526343	-0.853357	1.858261
5	8	0	-0.382275	1.023094	2.555162
6	7	0	-0.998294	0.265814	-0.528450
7	1	0	-1.666129	-0.142233	0.139644
8	7	0	1.692702	-0.385875	-0.955045
9	1	0	1.656060	0.260857	-1.748958
10	6	0	-0.647800	-0.762621	-1.535865
11	6	0	0.681271	-1.444108	-1.198162
12	6	0	1.043572	-2.445263	-2.291854
13	6	0	-0.055478	-3.480122	-2.483570
14	6	0	-1.389156	-2.820406	-2.784759
15	6	0	-1.739698	-1.814404	-1.701258
16	6	0	-1.610998	1.505724	-1.075678
17	6	0	-2.071484	2.380142	0.082789
18	6	0	-2.623358	3.708647	-0.410673
19	6	0	-3.758881	3.502588	-1.400641
20	6	0	-3.306134	2.621682	-2.554908
21	6	0	-2.765244	1.288250	-2.051830
22	6	0	3.096651	-0.850238	-0.857074
23	6	0	3.342659	-1.684521	0.388898
24	6	0	4.783046	-2.179634	0.415985
25	6	0	5.770492	-1.025191	0.323259
26	6	0	5.501473	-0.176048	-0.912340
27	6	0	4.062775	0.323656	-0.918469
28	6	0	2.140502	2.707482	0.586088
29	6	0	3.016947	3.921129	0.555304
30	6	0	-0.066616	-0.170988	2.754728
31	6	0	-0.346931	-0.817748	4.080709
32	8	0	-3.193383	-0.834177	1.205768
33	6	0	-2.865608	-2.017495	1.938871
34	6	0	-3.883057	-3.055114	1.510435
35	6	0	-5.118622	-2.185531	1.313914
36	6	0	-4.516433	-0.947013	0.676580
37	1	0	5.670736	-0.394267	1.219395
38	1	0	6.800414	-1.400425	0.315268

39	1	0	4.947338	-2.864892	-0.430427
40	1	0	4.958954	-2.764389	1.325759
41	1	0	2.652457	-2.535520	0.430936
42	1	0	3.135537	-1.068740	1.274286
43	1	0	3.317176	-1.478865	-1.736653
44	1	0	3.863191	0.925593	-1.816526
45	1	0	3.896684	0.964489	-0.044812
46	1	0	5.688385	-0.777820	-1.814998
47	1	0	6.192956	0.672966	-0.954746
48	1	0	0.550581	-1.979584	-0.246368
49	1	0	1.215889	-1.900136	-3.234401
50	1	0	1.979775	-2.958032	-2.048146
51	1	0	0.224581	-4.177498	-3.280414
52	1	0	-0.147088	-4.077001	-1.563906
53	1	0	-2.182198	-3.570572	-2.877006
54	1	0	-1.332333	-2.302532	-3.754400
55	1	0	-2.697774	-1.331883	-1.916801
56	1	0	-1.862214	-2.332029	-0.736298
57	1	0	-0.500533	-0.241373	-2.498025
58	1	0	-0.804871	2.033389	-1.610678
59	1	0	-2.847062	1.833392	0.643867
60	1	0	-1.243259	2.541609	0.777645
61	1	0	-2.955007	4.314454	0.440102
62	1	0	-1.811913	4.271570	-0.897725
63	1	0	-4.601355	3.014718	-0.885367
64	1	0	-4.131611	4.464039	-1.772560
65	1	0	-4.127744	2.448151	-3.259165
66	1	0	-2.516393	3.141783	-3.118236
67	1	0	-3.574557	0.743032	-1.540924
68	1	0	-2.450804	0.675246	-2.905107
69	1	0	-0.476596	-1.896710	3.973818
70	1	0	0.512152	-0.645311	4.737180
71	1	0	-1.225271	-0.372845	4.552350
72	1	0	-2.951150	-1.812553	3.016189
73	1	0	-1.821289	-2.275972	1.720447
74	1	0	-4.016313	-3.847854	2.249762
75	1	0	-3.586583	-3.515097	0.559220
76	1	0	-5.887954	-2.646518	0.690772
77	1	0	-5.563638	-1.940158	2.285228
78	1	0	-4.455129	-1.062424	-0.416843
79	1	0	-5.065267	-0.023796	0.892296
80	1	0	2.459809	4.798073	0.221633
81	1	0	3.467948	4.102716	1.531719
82	1	0	3.823766	3.740542	-0.164475

Cartesian coordinates (in Å) of DFT-II

SCF Done: E(UM06) = -1443.45892515

Center number	Atomic number	Atomic type	Coordinates (Angstroms) X	Y	Z
1	28	0	-0.045366	-0.780280	0.423644
2	8	0	-1.281818	-2.494180	0.087937
3	8	0	0.370155	-1.967891	-1.258310
4	8	0	-0.660824	-0.167785	2.274937

5	8	0	0.768512	-1.837738	2.299002
6	7	0	1.530692	0.588414	0.300381
7	1	0	1.859944	0.712071	1.260724
8	7	0	-1.102567	0.695342	-0.649700
9	1	0	-0.700197	0.615278	-1.588834
10	6	0	0.980662	1.877426	-0.180581
11	6	0	-0.542829	1.923544	-0.033121
12	6	0	-1.079652	3.234372	-0.601701
13	6	0	-0.439288	4.437073	0.075825
14	6	0	1.074384	4.389869	-0.030032
15	6	0	1.603932	3.077809	0.524016
16	6	0	2.645668	0.032793	-0.512242
17	6	0	3.213762	-1.187242	0.200134
18	6	0	4.298340	-1.858003	-0.628579
19	6	0	5.412159	-0.882834	-0.975512
20	6	0	4.848000	0.342026	-1.678894
21	6	0	3.770050	1.014207	-0.836345
22	6	0	-2.574177	0.678042	-0.823966
23	6	0	-3.311538	0.617384	0.503293
24	6	0	-4.816821	0.654406	0.272342
25	6	0	-5.265152	-0.463960	-0.657520
26	6	0	-4.502818	-0.415336	-1.975322
27	6	0	-3.000648	-0.467797	-1.729705
28	6	0	-0.582433	-2.738903	-0.932354
29	6	0	-0.916908	-3.911706	-1.801091
30	6	0	-0.003289	-1.059438	2.902219
31	6	0	-0.202322	-1.187562	4.385145
32	1	0	-5.077575	-1.432690	-0.170084
33	1	0	-6.345111	-0.405068	-0.835803
34	1	0	-5.091776	1.624836	-0.169942
35	1	0	-5.342037	0.591992	1.231926
36	1	0	-3.012034	1.448209	1.153474
37	1	0	-3.024677	-0.306556	1.024608
38	1	0	-2.873245	1.607555	-1.337984
39	1	0	-2.449728	-0.420560	-2.679858
40	1	0	-2.746381	-1.415783	-1.243199
41	1	0	-4.759032	0.512069	-2.510281
42	1	0	-4.804574	-1.243923	-2.625756
43	1	0	-0.783878	1.875285	1.038142
44	1	0	-0.882616	3.263266	-1.685914
45	1	0	-2.165848	3.287177	-0.476574
46	1	0	-0.832562	5.362479	-0.358450
47	1	0	-0.726557	4.443510	1.137776
48	1	0	1.528578	5.234204	0.499230
49	1	0	1.372874	4.482635	-1.085300
50	1	0	2.693947	3.035215	0.445765
51	1	0	1.361707	3.004017	1.596548
52	1	0	1.199611	1.934651	-1.260454
53	1	0	2.189042	-0.297883	-1.457875
54	1	0	3.633157	-0.862792	1.167276
55	1	0	2.410391	-1.892304	0.427319
56	1	0	4.694635	-2.726152	-0.090540
57	1	0	3.848640	-2.243851	-1.556390
58	1	0	5.917006	-0.566010	-0.050149
59	1	0	6.172397	-1.367882	-1.598375
60	1	0	5.642214	1.061657	-1.906625

61	1	0	4.414239	0.039139	-2.643924
62	1	0	4.216130	1.370296	0.106487
63	1	0	3.388071	1.895794	-1.364568
64	1	0	-0.330455	-0.205806	4.845851
65	1	0	-1.119435	-1.757693	4.563782
66	1	0	0.630085	-1.718858	4.848057
67	1	0	-0.035938	-4.269598	-2.335764
68	1	0	-1.361997	-4.716103	-1.213663
69	1	0	-1.656353	-3.586561	-2.542032

Cartesian coordinates (in Å) of DFT-III

SCF Done: E(UM06) = -1443.46067270 A.U.

Center number	Atomic number	Atomic type	Coordinates (Angstroms) X	Y	Z
1	28	0	-0.114910	-0.740916	0.487179
2	8	0	-1.427338	-2.424876	0.321827
3	8	0	0.217070	-2.105008	-1.085116
4	8	0	-0.599438	-0.014455	2.392781
5	8	0	0.767146	-1.712733	2.216438
6	7	0	1.531387	0.563520	0.278422
7	1	0	1.907598	0.655667	1.224560
8	7	0	-1.053598	0.747566	-0.695679
9	1	0	-0.622556	0.640174	-1.618564
10	6	0	1.031724	1.884155	-0.169042
11	6	0	-0.492642	1.967982	-0.071099
12	6	0	-0.968356	3.290024	-0.662393
13	6	0	-0.322531	4.467932	0.056634
14	6	0	1.194590	4.378868	0.030415
15	6	0	1.666984	3.044057	0.585348
16	6	0	2.587829	-0.027507	-0.585949
17	6	0	3.109319	-1.308801	0.045980
18	6	0	4.117272	-1.997378	-0.862599
19	6	0	5.276302	-1.075501	-1.208086
20	6	0	4.765429	0.223870	-1.811269
21	6	0	3.757392	0.905070	-0.892313
22	6	0	-2.520034	0.743656	-0.898498
23	6	0	-3.268214	0.679710	0.423456
24	6	0	-4.773237	0.672625	0.187240
25	6	0	-5.185580	-0.464415	-0.737116
26	6	0	-4.426523	-0.392216	-2.055074
27	6	0	-2.924137	-0.405268	-1.810060
28	6	0	-0.748405	-2.805913	-0.666950
29	6	0	-1.120312	-4.060547	-1.395882
30	6	0	0.108496	-0.917486	2.929480
31	6	0	0.126054	-1.036326	4.424207
32	1	0	-4.962920	-1.424489	-0.246786
33	1	0	-6.267114	-0.442877	-0.913816
34	1	0	-5.075026	1.631084	-0.263399
35	1	0	-5.299194	0.601983	1.145766
36	1	0	-2.991787	1.525931	1.064411
37	1	0	-2.958896	-0.230464	0.956241
38	1	0	-2.810713	1.674677	-1.415273

39	1	0	-2.373639	-0.341043	-2.758835
40	1	0	-2.648128	-1.349481	-1.328071
41	1	0	-4.707524	0.529678	-2.587152
42	1	0	-4.705884	-1.226374	-2.708332
43	1	0	-0.769721	1.932832	0.991776
44	1	0	-0.720954	3.316632	-1.736079
45	1	0	-2.057055	3.374807	-0.585707
46	1	0	-0.663014	5.409209	-0.387800
47	1	0	-0.664114	4.474957	1.102274
48	1	0	1.639896	5.203769	0.596587
49	1	0	1.550795	4.481418	-1.005915
50	1	0	2.758243	2.970025	0.541340
51	1	0	1.385204	2.956971	1.647099
52	1	0	1.281981	1.974100	-1.239673
53	1	0	2.085529	-0.286413	-1.532596
54	1	0	3.589287	-1.054249	1.005799
55	1	0	2.278484	-1.977327	0.274161
56	1	0	4.480129	-2.915628	-0.387602
57	1	0	3.605399	-2.305847	-1.787259
58	1	0	5.843032	-0.849517	-0.291884
59	1	0	5.973679	-1.569226	-1.894488
60	1	0	5.594802	0.907184	-2.025209
61	1	0	4.281086	0.008919	-2.776060
62	1	0	4.250884	1.176014	0.055121
63	1	0	3.413789	1.838624	-1.353202
64	1	0	0.159729	-0.047065	4.885570
65	1	0	-0.802454	-1.517127	4.747168
66	1	0	0.968064	-1.641115	4.762473
67	1	0	-0.242059	-4.522921	-1.849160
68	1	0	-1.625051	-4.764555	-0.733006
69	1	0	-1.814908	-3.793828	-2.200698

Cartesian coordinates (in Å) of DFT-IV

SCF Done: E(UM06) = -1675.82109362

Center number	Atomic number	Atomic type	Coordinates (Angstroms) X	Y	Z
1	8	0	1.559041	-1.976528	0.988977
2	6	0	2.051714	-3.203678	0.427834
3	1	0	2.580311	-2.971739	-0.509486
4	1	0	1.199090	-3.847784	0.197517
5	6	0	2.992684	-3.781444	1.467253
6	1	0	2.439193	-4.401914	2.181540
7	1	0	3.778633	-4.393790	1.019823
8	6	0	3.507583	-2.526004	2.157188
9	1	0	3.932793	-2.710827	3.146164
10	1	0	4.271323	-2.036844	1.538803
11	6	0	2.252373	-1.685016	2.211206
12	1	0	1.613707	-1.971612	3.059116
13	1	0	2.414989	-0.606244	2.265285
14	8	0	-0.857622	-2.562159	-0.434653
15	8	0	0.427007	-1.419638	-1.788821
16	6	0	-0.252359	-2.454833	-1.531232

17	6	0	-0.355789	-3.544262	-2.555207
18	1	0	-1.193664	-3.312805	-3.222792
19	1	0	-0.557175	-4.506384	-2.080442
20	1	0	0.550923	-3.598933	-3.160838
21	28	0	-0.033628	-0.631178	0.112668
22	8	0	-0.668983	-0.420876	2.000582
23	8	0	0.788007	1.066495	2.835203
24	7	0	1.021883	1.212042	-0.015345
25	1	0	1.394686	1.273814	0.941513
26	7	0	-1.604436	0.600833	-0.697781
27	1	0	-1.271862	0.747007	-1.655432
28	6	0	0.046171	2.313529	-0.192682
29	1	0	0.091734	2.603671	-1.256958
30	6	0	-1.395727	1.863623	0.047113
31	1	0	-1.526099	1.626235	1.111708
32	6	0	-2.343667	3.007400	-0.305225
33	1	0	-2.262439	3.227732	-1.382247
34	1	0	-3.382188	2.716268	-0.117308
35	6	0	-2.025944	4.253108	0.511396
36	1	0	-2.711501	5.062523	0.237459
37	1	0	-2.209284	4.035152	1.574576
38	6	0	-0.579708	4.684476	0.337600
39	1	0	-0.356530	5.550558	0.970212
40	1	0	-0.414060	5.004846	-0.702966
41	6	0	0.361290	3.536406	0.663711
42	1	0	0.263452	3.245715	1.719192
43	1	0	1.402763	3.838742	0.519162
44	6	0	2.105280	1.210248	-1.029203
45	1	0	1.633838	0.873229	-1.967983
46	6	0	3.170170	0.197527	-0.641429
47	1	0	2.698196	-0.761843	-0.431744
48	1	0	3.645025	0.535288	0.295977
49	6	0	4.227231	0.053210	-1.724876
50	1	0	4.979194	-0.683585	-1.417080
51	1	0	3.751175	-0.347720	-2.633349
52	6	0	4.878633	1.391010	-2.040559
53	1	0	5.442596	1.732791	-1.159197
54	1	0	5.603389	1.288665	-2.856502
55	6	0	3.824079	2.431016	-2.389849
56	1	0	3.325075	2.139685	-3.326797
57	1	0	4.288888	3.405837	-2.575980
58	6	0	2.775762	2.559220	-1.289757
59	1	0	3.259498	2.902667	-0.361538
60	1	0	2.044544	3.325186	-1.573014
61	6	0	-3.014219	0.156472	-0.797810
62	1	0	-3.631187	1.021510	-1.093085
63	6	0	-3.546282	-0.368926	0.526348
64	1	0	-3.422635	0.378899	1.319842
65	1	0	-2.945006	-1.238212	0.824042
66	6	0	-5.013178	-0.757924	0.388457
67	1	0	-5.384523	-1.156565	1.339283
68	1	0	-5.610771	0.142055	0.174442
69	6	0	-5.217990	-1.769231	-0.731107
70	1	0	-6.280378	-2.019468	-0.833544
71	1	0	-4.696056	-2.702648	-0.471088
72	6	0	-4.662512	-1.243284	-2.048347

73	1	0	-4.785414	-1.987567	-2.843281
74	1	0	-5.236242	-0.356082	-2.357395
75	6	0	-3.191389	-0.878674	-1.898313
76	1	0	-2.629498	-1.778765	-1.631208
77	1	0	-2.783379	-0.494922	-2.844274
78	6	0	-0.225162	0.356233	2.907659
79	6	0	-1.087388	0.422441	4.149917
80	1	0	-0.534757	0.835865	4.995199
81	1	0	-1.491500	-0.560270	4.402643
82	1	0	-1.938962	1.081214	3.938414

Cartesian coordinates (in Å) of DFT-V

SCF Done: E(UM06) = -1443.44757604

Center number	Atomic number	Atomic type	Coordinates (Angstroms) X	Y	Z
1	6	0	3.389849	-0.129556	2.944008
2	6	0	2.091833	-0.122645	2.194419
3	8	0	1.957910	0.648668	1.197396
4	8	0	1.158998	-0.891646	2.529151
5	1	0	3.797668	0.880609	3.013626
6	1	0	4.110812	-0.740776	2.391474
7	1	0	3.264740	-0.558483	3.938712
8	6	0	-3.389849	0.129556	2.944008
9	6	0	-2.091833	0.122645	2.194419
10	8	0	-1.957910	-0.648668	1.197396
11	8	0	-1.158998	0.891646	2.529151
12	1	0	-3.264740	0.558483	3.938712
13	1	0	-3.797668	-0.880609	3.013626
14	1	0	-4.110812	0.740776	2.391474
15	7	0	0.493894	-1.359971	-0.627169
16	1	0	1.513720	-1.396899	-0.691858
17	7	0	-0.493894	1.359971	-0.627169
18	1	0	-1.513720	1.396899	-0.691858
19	6	0	-0.035850	-0.767635	-1.870463
20	6	0	0.035850	0.767635	-1.870463
21	6	0	-0.693424	1.259624	-3.124384
22	6	0	-0.041059	0.757776	-4.402315
23	6	0	0.041059	-0.757776	-4.402315
24	6	0	0.693424	-1.259624	-3.124384
25	6	0	0.019619	2.719245	-0.255253
26	6	0	-0.994039	3.400270	0.655400
27	6	0	-0.442365	4.703980	1.216759
28	6	0	0.000000	5.648596	0.110405
29	6	0	0.977305	4.956161	-0.828138
30	6	0	0.377736	3.673182	-1.393900
31	1	0	-0.881901	5.970201	-0.464729
32	1	0	0.446914	6.556235	0.532289
33	1	0	0.416430	4.474828	1.865018
34	1	0	-1.193726	5.181462	1.855715
35	1	0	-1.277641	2.726542	1.467458
36	1	0	-1.901304	3.610323	0.063050
37	1	0	0.941129	2.534635	0.316675
38	1	0	1.081745	3.216129	-2.099337

39	1	0	-0.529427	3.935164	-1.958744
40	1	0	1.902013	4.716344	-0.282164
41	1	0	1.264123	5.622717	-1.649280
42	1	0	1.101622	1.044568	-1.911658
43	1	0	-1.731050	0.892070	-3.070933
44	1	0	-0.754272	2.347779	-3.135964
45	1	0	-0.594577	1.120073	-5.275276
46	1	0	0.972384	1.180873	-4.475684
47	1	0	0.594577	-1.120073	-5.275276
48	1	0	-0.972384	-1.180873	-4.475684
49	1	0	0.754272	-2.347779	-3.135964
50	1	0	1.731050	-0.892070	-3.070933
51	1	0	-1.101622	-1.044568	-1.911658
52	6	0	-0.019619	-2.719245	-0.255253
53	6	0	0.994039	-3.400270	0.655400
54	6	0	0.442365	-4.703980	1.216759
55	6	0	0.000000	-5.648596	0.110405
56	6	0	-0.977305	-4.956161	-0.828138
57	6	0	-0.377736	-3.673182	-1.393900
58	1	0	0.881901	-5.970201	-0.464729
59	1	0	-0.446914	-6.556235	0.532289
60	1	0	-0.416430	-4.474828	1.865018
61	1	0	1.193726	-5.181462	1.855715
62	1	0	1.277641	-2.726542	1.467458
63	1	0	1.901304	-3.610323	0.063050
64	1	0	-0.941129	-2.534635	0.316675
65	1	0	-1.081745	-3.216129	-2.099337
66	1	0	0.529427	-3.935164	-1.958744
67	1	0	-1.902013	-4.716344	-0.282164
68	1	0	-1.264123	-5.622717	-1.649280
69	28	0	0.000000	0.000000	0.905014

Supplementary References

1. Evans, D. A. & Seidel, D. Ni(II)-Bis[(*R*,*R*)-*N,N*'-dibenzylcyclohexane-1,2-diamine]-Br_2 catalyzed enantioselective Michael additions of 1,3-dicarbonyl compounds to conjugated nitroalkenes. *J. Am. Chem. Soc.* **127**, 9958–9959 (2005).
2. Evans, D. A., Mito, S. & Seidel, D. Scope and mechanism of enantioselective Michael additions of 1,3-dicarbonyl compounds to nitroalkenes catalyzed by nickel(II)–diamine complexes. *J. Am. Chem. Soc.* **129**, 11583–11592 (2007).
3. Wilckens, K., Duhs, M.-A., Lentz, D. & Czekelius, C. Chiral 1,1'-bi(tetrahydroisoquinoline)-type diamines as efficient ligands for nickel-catalysed enantioselective Michael addition to nitroalkenes. *Eur. J. Org. Chem.* 5441–5446 (2011).
4. Fossey, J. S., Matsubara, R., Paulo, V. & Kobayashi, S. A C_2-symmetric nickel diamine complex as an asymmetric catalyst for enecarbamate additions to butane-2,3-dione. *Org. Biomol. Chem.* **3**, 2910–2913 (2005).
5. Evans, D. A., Downey, C. W. & Hubbs, J. L. Ni(II) bis(oxazoline)-catalyzed enantioselective *syn* aldol reactions of *N*-propionylthiazolidinethiones in the presence of silyl triflates. *J. Am. Chem. Soc.* **125**, 8706–8707 (2003).
6. Spielvogel, D. J., Davis, W. M. & Buchwald, S. L. Application of [(S)-BINAP]Ni(COD) and [(S)-BINAP]NiBr_2. *Organometallics* **21**, 3833–3836 (2002).

7. Kanemasa, S., Oderatoshi, Y., Yamamoto, H., Tanaka, J. & Wada, E. Cationic aqua complexes of the C_2-symmetric *trans*-chelating ligand (*R*,*R*)-4,6-dibenzofurandiyl-2,2'-bis(4-phenyloxazoline). Absolute chiral induction in Diels-Alder reactions catalyzed by water-tolerant enantiopure Lewis acids. *J. Org. Chem.* **62**, 6454–6455 (1997).
8. Zhou, Y.-Y., Wang, L.-J., Li, J., Sun, X.-L. & Tang, Y. Side-arm-promoted highly enantioselective ring-opening reactions and kinetic resolution of donor − acceptor cyclopropanes with amines. *J. Am. Chem. Soc.* **134**, 9066–9069 (2012).
9. Deng, Q.-H., Wadepohl, H. & Gade, L. H. The synthesis of a new class of chiral pincer ligands and their applications in enantioselective catalytic fluorinations and the Nozaki–Hiyama–Kishi Reaction. *Chem. Eur. J.* **17**, 14922–14928 (2011).
10. Schley, N. D. & Fu, G. C. Nickel-catalyzed Negishi arylations of propargylic bromides: a mechanistic investigation. *J. Am. Chem. Soc.* **136**, 16588–16593 (2014).
11. Rettenmeier, C., Wadepohl, H. & Gade, L. H. Stereoselective hydrodehalogenation via a radical-based mechanism involving T-shaped chiral nickel(I) pincer complexes. *Chem. Eur. J.* **20**, 9657–9665 (2014).
12. Alecu, I. M., Zheng, J., Zhao, Y. & Truhlar, D. G. *J. Chem. Theory Comput.* Computational Thermochemistry: Scale Factor Databases and Scale Factors for Vibrational Frequencies Obtained from Electronic Model Chemistries **6**, 2872-2887 (2010).
13. Lady, J. H. & Whetsel, K. B. Infrared studies of amine complexes. IV. The N-H---O hydrogen bond in aromatic amine complexes of ethers, ketones, esters, and amides. *J. Phys. Chem.* **71**, 1421-1429 (1967).
14. Arnold, M., Brown, D. A., Deeg, O., Errington, W., Haase, W., Herlihy, K., Kemp, T. J., Nimir, H. & Werner, R. Hydroxamate-bridged dinuclear nickel complexes as models for urease inhibition. *Inorg. Chem.* **37**, 2920–2925 (1998).
15. Nakamura, A., Lectard, S., Hashizume, D., Hamashima, Y. & Sodeoka, M. Diastereo- and enantioselective conjugate addition of α-ketoesters to nitroalkenes catalyzed by chiral $Ni(OAc)_2$ complex under mild conditions. *J. Am. Chem. Soc.* **132**, 4036–4037 (2010).
16. Gella, C., Ferrer, È., Alibés, R., Busqué, F., de March, P., Figueredo, M. & Font, J. A metal-free general procedure for oxidation of secondary amines to nitrones. *J. Org. Chem.* **74**, 6365–6367 (2009).
17. Soeta, T., Fujinami, S. & Ukaji, Y. Chlorosilane-promoted addition reaction of isocyanides to 3,4-dihydroisoquinoline *N*-oxides. *J. Org. Chem.* **77**, 9878–9883 (2012).
18. Kano, S., Yokomatsu, T., Yuasa, Y. & Shibuya, S. A formation of pyrrolo[2,1-a] isoquinoline derivatives by the reaction of isoquinoline *N*-oxides with ethyl propiolate. *Heterocycles*, **19**, 2143–2145 (1982).
19. Wu, J. Wang, C. Tang, W. Pettman, A. & Xiao, J. The remarkable effect of a simple ion: iodide-promoted transfer hydrogenation of heteroaromatics *Chem. Eur. J.* **18**, 9525–9529 (2012).
20. Sall, D. J. & Grunewald, G. L. Inhibition of phenylethanolamine *N*-methyltransferase (PNMT) by aromatic hydroxy-substituted 1,2,3,4-tetrahydroisoquinolines: further studies on the hydrophilic pocket of the aromatic ring binding region of the active site *J. Med. Chem.* **30**, 2208–2216 (1987).
21. Zhou, D., Gross, J. L., Adedoyin, A. B. Aschmies, S. B. Brennan, J., Bowlby, M., Di, L., Kubek, K., Wang, B.J. Z., Zhang, G., Brandon, N., Comery, T. A. & Robichaud, A. J. *J. Med. Chem.* **55**, 2452–2468 (2012).

22. Mimoun, H., de Saing Laumer, J. Y., Giannini, L., Scopelliti, R & Floriani, C. Enantioselective reduction of ketones by polymethylhydrosiloxane in the presence of chiral zinc catalysts. *J. Am. Chem. Soc.* **121**, 6158–6166 (1999).
23. Chen, Z., Lin, L., Wang, M., Liu, X. & Feng, X. Asymmetric synthesis of *trans*-β-lactams by a Kinugasa reaction on water. *Chem. Eur. J.* **19**, 7561–7567 (2013).
24. Otwinoski, Z. & Minor, W. Processing of X-ray diffraction data collected in oscillation mode. *Methods in Enzymol.* **276**, 307–326 (1997).
25. Alcock, N. W. *Cryst. Computing*, Analytical method for absorption correction 271 (1970).
26. Burla, M. C., Caliandro, R., Camalli, M., Carrozzini, B., Cascarano, G. L., Caro, L., De Giacovazzo, C., Polidori, G. & Spagna, R. *SIR2004*: an improved tool for crystal structure determination and refinement. *J. Appl. Cryst.* **38**, 381–388 (2005).
27. Sheldrick, G. M. Crystal structure refinement with SHELXL. *Acta Crystallogr. Sect. C*, *C71*, 3–8 (2015).
28. Hansen, N. K. & Coppens, P. Testing aspherical atom refinements on small-molecule data sets. *Acta Crystallogr. Sect. A A34*, 909–921 (1978).
29. Volkov, A., Macchi, P., Farrugia, L. J., Gatti, C., Mallinson, P. R., Richter, T., Koritsanszky, T. XD2006 – a computer program for multipole refinement, topological analysis of charge densities and evaluation of intermolecular energies from experimental or theoretical structure factors (2006).
30. Holladay, A., Leung, P. & P. Coppens, Generalized relations between *d*-orbital occupancies of transition-metal atoms and electron-density multipole population parameters from X-ray diffraction data. *Acta Crystallogr. Sect. A.* **A39**, 377-387 (1983).
31. Zhao, Y. & Truhlar, D. G. Density functionals with broad applicability in chemistry. *Acc. Chem. Res.* **41**, 157–167 (2008).
32. Frisch, M. J., Trucks, G. W., Schlegel, H. B., Scuseria, G. E., Robb, M. A.; Cheeseman, J. R., Scalmani, G.; Barone, V., Mennucci, B.; Petersson, G. A., Nakatsuji, H.; Caricato, M., Li, X.; Hratchian, H. P., Izmaylov, A. F.; Bloino, J., Zheng, G.; Sonnenberg, J. L., Hada, M.; Ehara, M., Toyota, K.; Fukuda, R., Hasegawa, J.; Ishida, M., Nakajima, T.; Honda, Y., Kitao, O.; Nakai, H., Vreven, T.; Montgomery, J. A., Jr., Peralta, J. E.; Ogliaro, F., Bearpark, M.; Heyd, J. J., Brothers, E.; Kudin, K. N., Staroverov, V. N.; Kobayashi, R., Normand, J.; Raghavachari, K., Rendell, A.; Burant, J. C., Iyengar, S. S.; Tomasi, J., Cossi, M.; Rega, N., Millam, J. M.; Klene, M., Knox, J. E.; Cross, J. B., Bakken, V.; Adamo, C., Jaramillo, J.; Gomperts, R., Stratmann, R. E.; Yazyev, O., Austin, A. J.; Cammi, R., Pomelli, C.; Ochterski, J. W., Martin, R. L.; Morokuma, K., Zakrzewski, V. G.; Voth, G. A., Salvador, P.; Dannenberg, J. J., Dapprich, S.; Daniels, A. D., Farkas, Ö.; Foresman, J. B., Ortiz, J. V.; Cioslowski, J., Fox, D. J., Gaussian 09, Revision E.01 (Gaussian, Inc.: Wallingford, CT, 2009).
33. Stratmann, R. E., Scuseria, G. E. & Frisch, M. J. *J. Chem. Phys.* An efficient implementation of time-dependent density-functional theory for the calculation of excitation energies of large molecules. **109**, 8218–8224 (1998).
